# Indole Peptidomimetics Show Potent and Selective Activity against Drug-Resistant *Plasmodium falciparum*

**DOI:** 10.1021/acsomega.5c12662

**Published:** 2026-03-21

**Authors:** Marcelo Augusto Pereira Januário, Talita Alvarenga Valdes, Sarah El Chamy Maluf, Giovana Rossi Mendes, Vinicius Bonatto, Igor M. R. Moura, Penina S. Mourão, Arlene G. Corrêa, Rafael Victorio Carvalho Guido

**Affiliations:** † Centre of Excellence for Research in Sustainable Chemistry, Department of Chemistry, Federal University of São Carlos, São Carlos 13565-905, Brazil; ‡ São Carlos Institute of Physics, University of São Paulo, São Carlos 13563-120, Brazil

## Abstract

Malaria remains a global health challenge exacerbated by emerging drug-resistant *Plasmodium falciparum* strains. Here, we report the design, synthesis, and biological evaluation of indole-based peptidomimetics against *P. falciparum* sensitive and multidrug-resistant strains. Structure–activity relationship analysis indicated that aromatic and halogen substituents, as well as modifications at the indole nitrogen and amide linkage, strongly influence potency and selectivity. **LSPN954** (**4i**) and **LSPN959** (**4k**) emerged as front-runner compounds, displaying low micromolar potency against the sensitive strain (IC_50_^3D7^ = 1.7 and 1.0 μM, respectively), low cytotoxic effects on HepG2 and HEK293 human cells (CC_50_ ≥ 100 μM), and high selectivity indices (SI = 59 and 95, respectively). In addition, these compounds demonstrated a slow-acting profile, additive effects with artesunate, and retained efficacy against multiple resistant strains (Dd2, K1, Dd2^R^_DSM265, and 3D7^R^_MMV848), exhibiting no cross-resistance. These findings highlight indole peptidomimetics as promising scaffolds for antimalarial drug development, providing a foundation for further optimization toward potent, selective agents capable of overcoming current resistance mechanisms.

## Graphical abstract

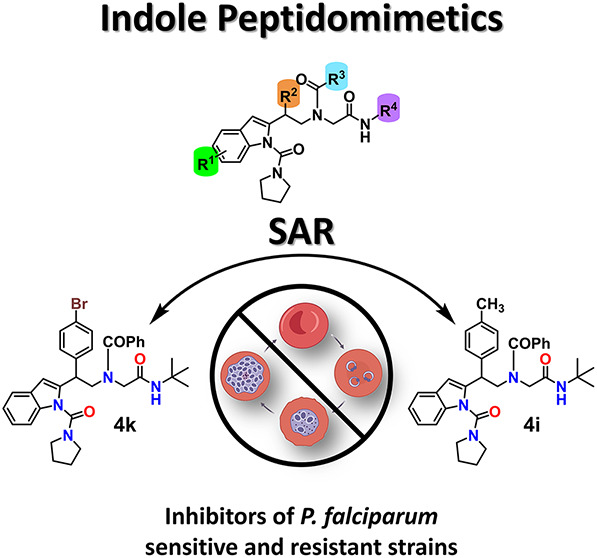

## Introduction

Malaria, a millennia-old parasitic disease caused by protozoa of the *Plasmodium* genus, remains one of the greatest challenges in global public health. It is recognized by the World Health Organization (WHO) as one of the leading infectious diseases with the potential to cause death.^1^ Historical evidence, including reports of febrile illnesses in China around 2700 BCE and in Greece around 800 BCE, attests to the long-standing coexistence of malaria with humanity.^2^

In 2024, malaria affected approximately 282 million people, resulting in 610,000 deaths across 83 countries on five continents. The African Region was disproportionately impacted, accounting for 94% of cases and 95% of deaths, with 52% of the global burden concentrated in five countries.^1^ Children under five years old represent 76% of all malaria-related deaths in the region, a figure that has remained unchanged since 2015.^3^ Despite progress in malaria control, the mortality rate is nearly three times higher than the 75% reduction target set by the Global Technical Strategy for Malaria 2016–2030, which was expected to be achieved by 2025.^1^

Although malaria is a treatable disease, its eradication is hindered by the emergence and spread of resistance in both parasites and mosquitoes to current interventions, including antimalarial drugs and insecticides.^4,5^ The first-line treatment for uncomplicated *P. falciparum* malaria is oral artemisinin-based combination therapy (ACT),^6^ which remains highly effective in most endemic regions. However, the emergence and spread of partial artemisinin resistance, as well as resistance to ACT partner drugs, is a growing concern. In Africa, partial artemisinin resistance has been reported in several countries, posing a serious threat to global malaria control and elimination efforts.^7^

An effective antimalarial drug should protect against infection, eliminate the parasite, be safe and well tolerated, including in pregnant women and children, offer a simple dosing regimen, be affordable and easy to administer, and retain activity against circulating drug-resistant strains.^8^ Consequently, the search for new bioactive compounds with antiplasmodial activity remains a priority in antimalarial drug discovery.

Nitrogen-containing heterocycles are prevalent in many pharmaceuticals and frequently explored in drug design.^9^ Among these, the indole scaffold is considered a privileged structural motif,^10^ due to its significant role in the development of novel antimalarial agents.^11–15^ Both natural and synthetic indole derivatives, including indole alkaloids,^16^ indolinones,^17^ aryl-indolinones,^18^ indole-carboxamides,^19^ and indolizinoindolones^20^ have demonstrated inhibitory activity against *P. falciparum*.

Herein, we synthesized and evaluated a series of indole-based peptidomimetics for their antiplasmodial and cytotoxic effects. Two hits were further investigated for their speed of action, efficacy in combination with artesunate, and inhibitory activity against a panel of drug-resistant *P. falciparum* strains.

## Results

### Chemistry and Structure–Activity Relationship Analysis

Continuing our efforts in the search for new antimalarial compounds,^21–23^ recently, we have synthesized highly functionalized indole derivatives **3** via Rh (III)-catalyzed C-2 alkylation with nitroolefins.^24^ Moreover, the nitro compounds were reduced and submitted to the Ugi four-component reaction, furnishing a series of new indole-peptidomimetics **4a**-**m** in moderate to good overall yields (Scheme 1).

**Scheme 1:**
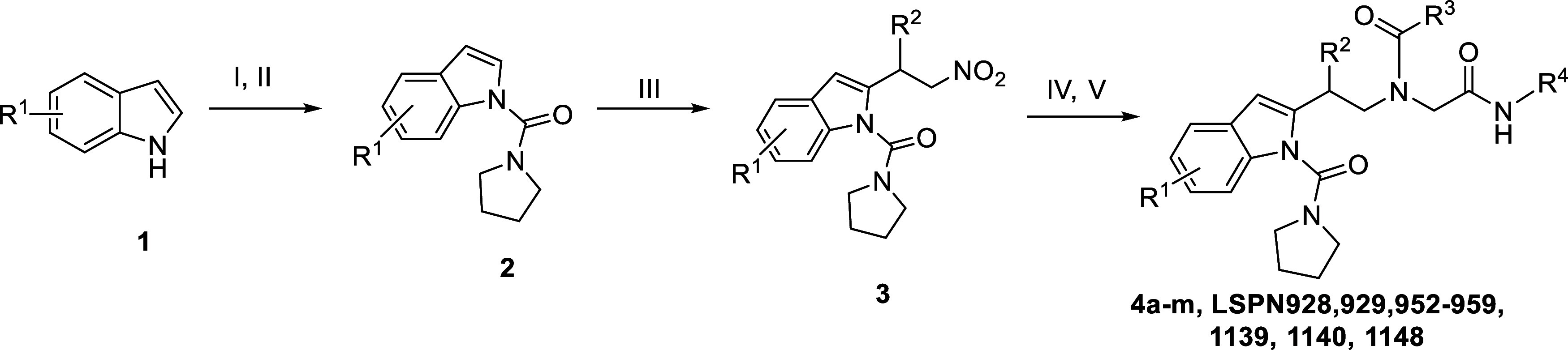
Synthesis of New Indole-Peptidomimetics **4a**–**m** ^a^

a (I) Pyrrolidine, anhydrous DCM, pyridine (10 mol %), triphosgene (0.5 equiv), 1 h; (II) NaH (1.5 equiv), anhydrous THF, 30 min; *N*-pyrrolidinylcarbamoyl chloride (1.5 equiv), 16 h, r.t.; (III) Ar­(CH)_2_NO_2_ (3 equiv), [Cp*RhCl_2_]_2_ (10 mol %), AgSbF_6_ (20 mol %), AgOAc (40 mol %), dry DCE, N_2_, 85 °C, 72 h; (IV) NiCl_2_.6H_2_0 (3 equiv), NaBH_4_ (1 equiv), MeOH, 0 °C, 0.5–1 h; (V) –(CH_2_O)­n–, R_3_CO_2_H, R_4_NC, MeOH, 55 °C, 72 h.

The antiplasmodial activity and cytotoxicity of these new indole derivatives **3a**,**b** and **4a**–**m** are shown in Table 1. Briefly, the peptidomimetics **4**, obtained via Ugi reaction, are more potent than the nitro compounds **3**. Substitution of an alkyl chain (e.g., heptyl: **LSPN928 (4d)**; IC_50_^3D7^ = 13 μM) with an aromatic substituent (e.g., phenyl: **LSPN952** (**4a**); IC_50_^3D7^ = 3.6 μM) at the R^3^ position of resulted in a 4-fold improvement in potency and a 2-fold reduction in cytotoxicity (Table 1). However, the substitution of a hydrogen atom of the indole moiety (e.g., **LSPN952** (**4a**)) with a methyl group (**LSPN953** (**4g**); IC_50_^3D7^ = 12 μM) was detrimental for the inhibitory activity, determining a 3-fold loss in potency against the parasite. In contrast, the substitution of a hydrogen atom with a methyl group to the phenyl ring at the R^2^ position of **LSPN952** (**4a**) increased the inhibitory potency by 2- and 3-fold for **LSPN954** (**4i**, IC_50_^3D7^ = 1.7 μM) and **LSPN1139** (**4m**, IC_50_^3D7^ = 1 μM), respectively. A similar trend was observed with the substitution with a methoxy group at the R^2^ phenyl position, as observed in **LSPN955** (**4j**, IC_50_^3D7^ = 1.8 μM).

**Table 1:**
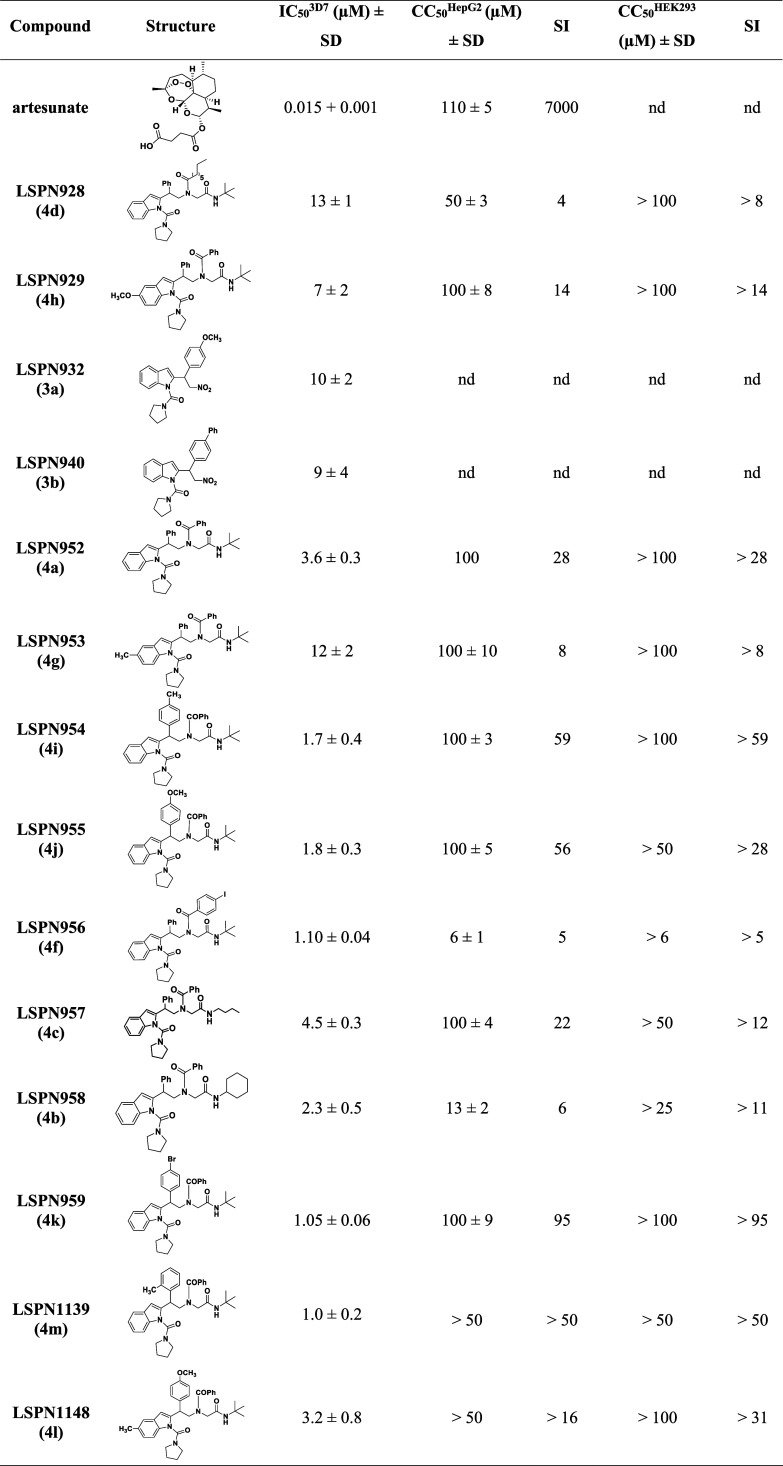
*In Vitro* Inhibitory Activity of Indole Derivatives against the Chloroquine-Sensitive *P. falciparum* 3D7 Strain, Human Hepatocarcinoma (HepG2) Cells, and Human Embryonic Kidney (HEK293) Cells^a^

a Data represent the mean and standard deviation (SD) values of at least two independent experiments. SI: selectivity index = CC_50_^HepG2 or HEK293^/IC_50_^3D7^; nd = not determined.

Interestingly, replacing a hydrogen atom with a halogen also improved potency against 3D7 strains. For example, the substitution with a *p*-bromine (**LSPN959** (**4k)**, IC_50_ = 1.05 μM) at the R^2^ group or a *p*-iodine (**LSPN956** (**4f**); IC_50_ = 1.1 μM) at the R^3^ phenyl position resulted in more potent compounds. However, the latter derivative exhibited increased cytotoxic effect on HepG2 and HEK293 cells.

Based on these results, and with the objective to contribute with the structure–activity relationship study, we have synthesized a new series of indole peptidomimetics. Thus, employing commercially available 1*H*-indole-2-carboxylic acid (**5a**) and 2-(1*H*-indol-2-yl) acetic acid (**5b**) as starting materials for the Ugi reaction, derivatives **LSPN1141–1143**, **LSPN1151** (**6a**-**d**) and **LSPN1149** (**6g**), respectively, were efficiently synthesized (Scheme 2). To evaluate the effect of the *N*-indole protecting group, we have prepared the corresponding benzyl (**LSPN1146, 6e**) and tosyl (**LSPN1150, 6f**) derivatives. Moreover, indole **5a** was also used to produce the corresponding amide **7**, which, after reduction, afforded amine **8**, that was submitted to the Ugi reaction, furnishing indole derivatives **LSPN1152** (**9a**) and **LSPN1156** (**9d**). The α-acylaminoamide **LSPN1152** (**9a**) was then protected affording derivatives **LSPN1153–1156** (**9b**–**e**) (Scheme 3).

**Scheme 2:**
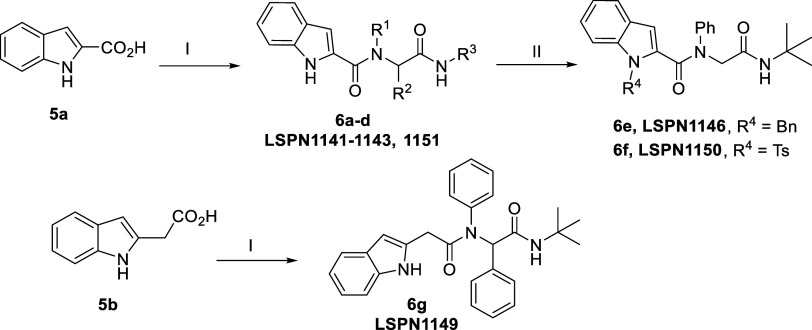
Synthesis of Indole Derivatives **6a**–**g** ^25^ ^a^

a (I) **5a** or **5b**, R^2^CHO, R^1^NH_2_, R^3^NC (1 equiv), MeOH, r.t., 24 h; (II) **6e**: **6a**, BnBr (1.5 equiv), Cs_2_CO_3_ (1.5 equiv), DMF, 155 °C, 24 h; **6f**: **6b**, NaH (1.5 equiv), 0 °C, 30 min; TsCl (1.5 equiv), r.t. 24 h.

**Scheme 3:**
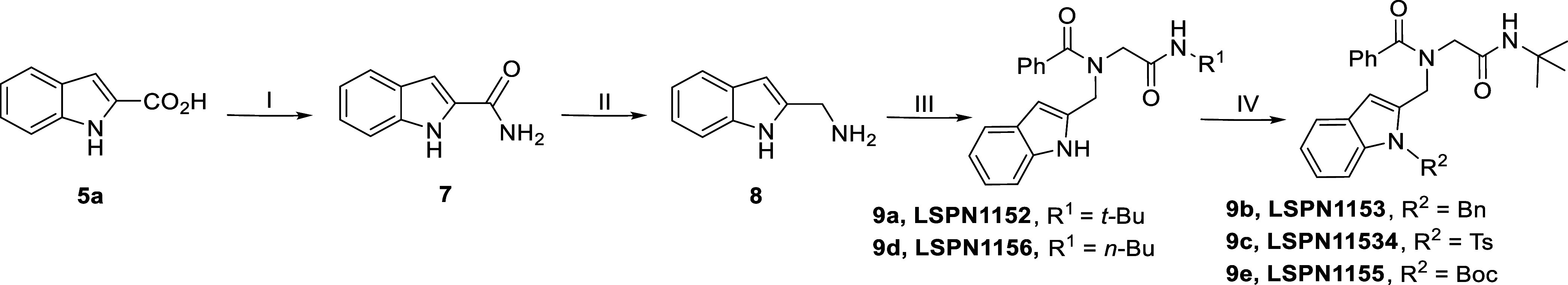
Synthesis of Indole Derivatives **9a**–**e^a^**

a (I) (a) SOCl_2_ (1.5 equiv), toluene, reflux, 2 h; (b) 1,4-dioxane, 0 °C, NH_3_ solution (1.5 equiv), 1 h; (II) LiAlH_4_ (2 equiv), dry THF, 0 °C, 10 min, reflux, 7h; (III) –(CH_2_O)­n–, PhCO_2_, R^1^NC (1 equiv), MeOH, 24 h; 0 °C; (IV) **9b**: Cs_2_CO_3_ (1.5 equiv), BnBr (1.5 equiv), DMF, 155 °C; **9c**: NaH (1.5 equiv), dry DMF, 0 °C, 30 min, then TsCl (1.5 equiv), r.t., 24 h; 9e: NaH (1.5 equiv), dry DMF, 0 °C, 30 min, then Boc_2_O (1.5 equiv), r.t., 24 h.

Finally, to evaluate the effect of the side chain position in the indole core, we have prepared the C3-alkylated derivatives **10**. To this end, the Michael addition reaction of indole on two different β-nitrostyrenes, using the solvent-free protocol described by Habib et al.,^25^ afforded the desired products **10a**,**b** (Scheme 4). Reduction of the nitro group followed by Ugi reaction furnished the desired indole peptidomimetics **LSPN1147** and **LSPN1157–1159** (**12a-d**).

**Scheme 4:**
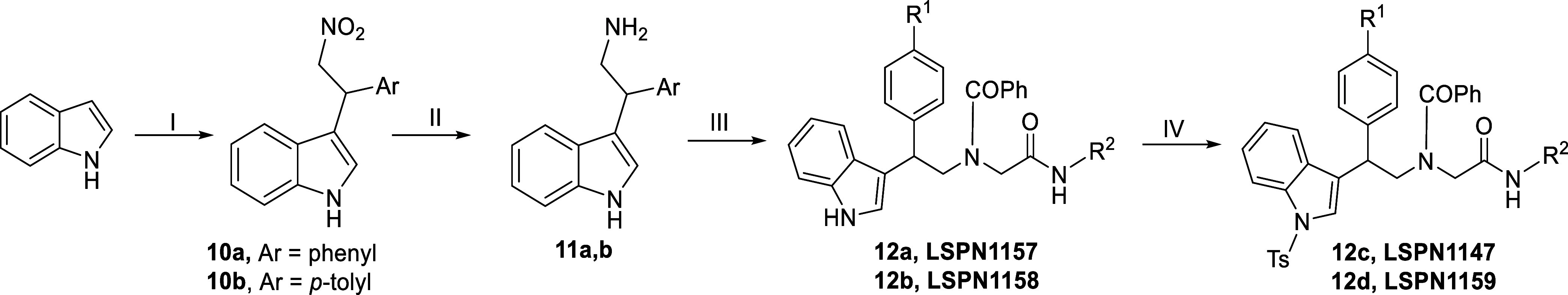
Synthesis of Indole Peptidomimetics **12a**-**d^a^**

a (I) Ar­(CH)_2_NO_2_ (1.2 equiv), 100 °C, 12 h; (II) NiCl_2_. 6H_2_0 (1 equiv), NaBH_4_ (3 equiv), 0 °C, MeOH, 30 min.; (III) PhCO_2_H, (CH_2_O)­n, R_2_NC (1 equiv), MeOH, r.t., 24 h. (IV) NaH (1.5 equiv), 0 °C, 30 min; then TsCl (1.5 equiv), r.t. 24 h.

Introducing an iodine in the phenyl ring at the R^1^ position of **LSPN1141** (**6b**, IC_50_^3D7^ = 30 μM) led to a 3-fold gain in the potency (**LSPN1142** (**6c**), IC_50_^3D7^ = 8 μM), and a moderate selectivity (SI = 12) (Table 2). The presence of the phenyl ring at the R^2^ position appears critical for potency, as its removal rendered inactive compounds (e.g., **LSPN1143** and **LSPN1151**; IC_50_^3D7^ > 50 μM). However, if the phenyl ring at R^2^ is removed and a bulkier group is attached to the nitrogen of the indole ring, the antiplasmodial activity is restored (e.g., **LSPN1146** (**6e**) and **LSPN1150** (**6f**); IC_50_s^3D7^ = 6 μM). Additionally, adding a methylene group as a spacer to connect the indole ring to the amide group resulted in a low micromolar inhibitor (**LSPN1149** (**6g**); IC_50_^3D7^ = 6 μM) (Table 2).

**Table 2:**
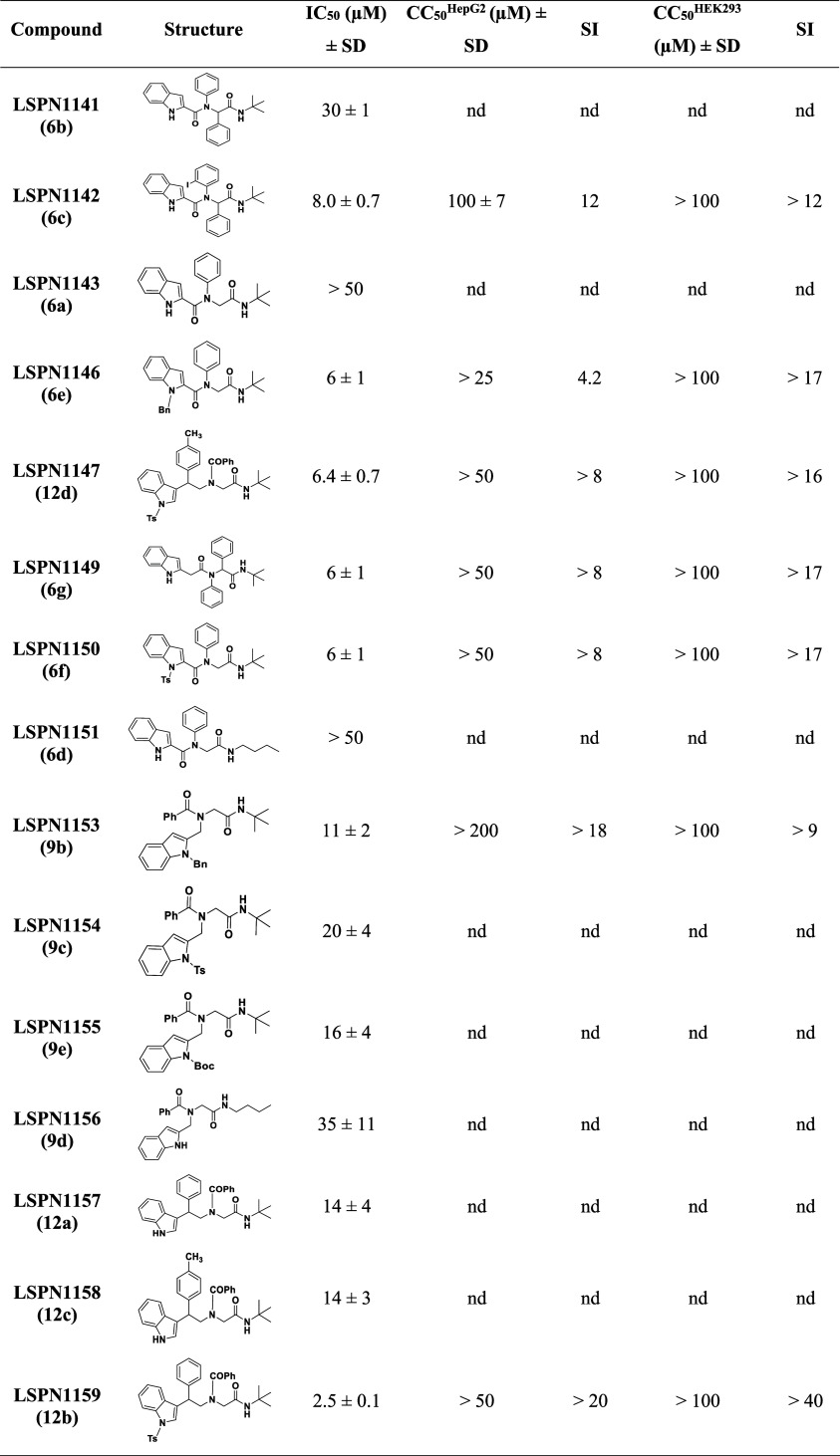
*In Vitro* Inhibitory Activity of Indole Derivatives against the Chloroquine-Sensitive *P. falciparum* 3D7 Strain and Human Hepatocarcinoma (HepG2) Cells, and Human Embryonic Kidney (HEK293) Cells^a^

a Data represent the mean and standard deviation (SD) values of at least two independent experiments. **LSPN1156** was tested in four independent experiments. SI: selectivity SI: selectivity index = CC_50_^HepG2orHEK293^/IC_50_^3D7^; nd = not determined.

Substitutions at the nitrogen of the indole ring had minimal impact on potency. Replacing the hydrogen atom with bulkier groups showed negligible effects (e.g., **LSPN1153** (**9b**), **LSPN1154** (**9c**), and **LSPN1155** (**9e**)). However, the substitution of a hydrogen atom with a tosyl group (**LSPN1159** (**12b**); IC_50_^3D7^ = 2.5 μM), led to a 6-fold improvement in potency, while maintaining low cytotoxicity (CC_50_^HepG2^ > 50 μM and CC_50_^HEK293^ > 100 μM) and moderate selectivity (SI^HepG2^ > 20 and SI^HEK293^ > 40) (Table 2).

Based on these results, we selected **LSPN954** (**4i**) and **LSPN959** (**4k**) for further investigations due to their superior antiplasmodial activity (IC_50_^3D7^ values of 1 μM) and promising selectivity indexes (SI values >50).

### Indole Derivatives Are Slow-Acting Compounds against *P. falciparum*

To further explore the antiplasmodial profile of the selected compounds, the speed of action was determined against the asexual intraerythrocytic stages of *P. falciparum*. Initially, we assessed whether the derivatives exhibited fast or slow-acting properties. In this assay, we used an adapted protocol from Le Manach et al.^26^ Briefly, the IC_50_ values of both **LSPN954** and **LSPN959** were determined at different exposure times (24, 48, and 72 h) and compared. Three 96-well plates, representing the analysis intervals, were prepared using synchronized ring-stage *P. falciparum* 3D7 cultures.^27^ Parasites treated with artesunate and chloroquine served as fast-acting controls, while pyrimethamine and atovaquone were used as slow-acting controls.^28,29^ Negative controls consisted of parasites cultured with blood and medium without the addition of compounds.

Incubation with fast-acting antimalarials, such as artesunate and chloroquine, for 24 h results in IC_50_ values comparable to those obtained in the standard 72 h assay, with IC_50_^24h^/IC_50_^72h^ ratios close to 1. In contrast, slow-acting inhibitors like atovaquone and pyrimethamine exhibit IC_50_^24h^/IC_50_^72h^ ratios greater than 1. The compounds **LSPN954** and **LSPN959**, showed IC_50_^24h^/IC_50_^72h^ ratios above 1, indicating a slow-acting profile similar to that of the pyrimethamine and atovaquone controls (Figure 1a).

**Figure 1:**
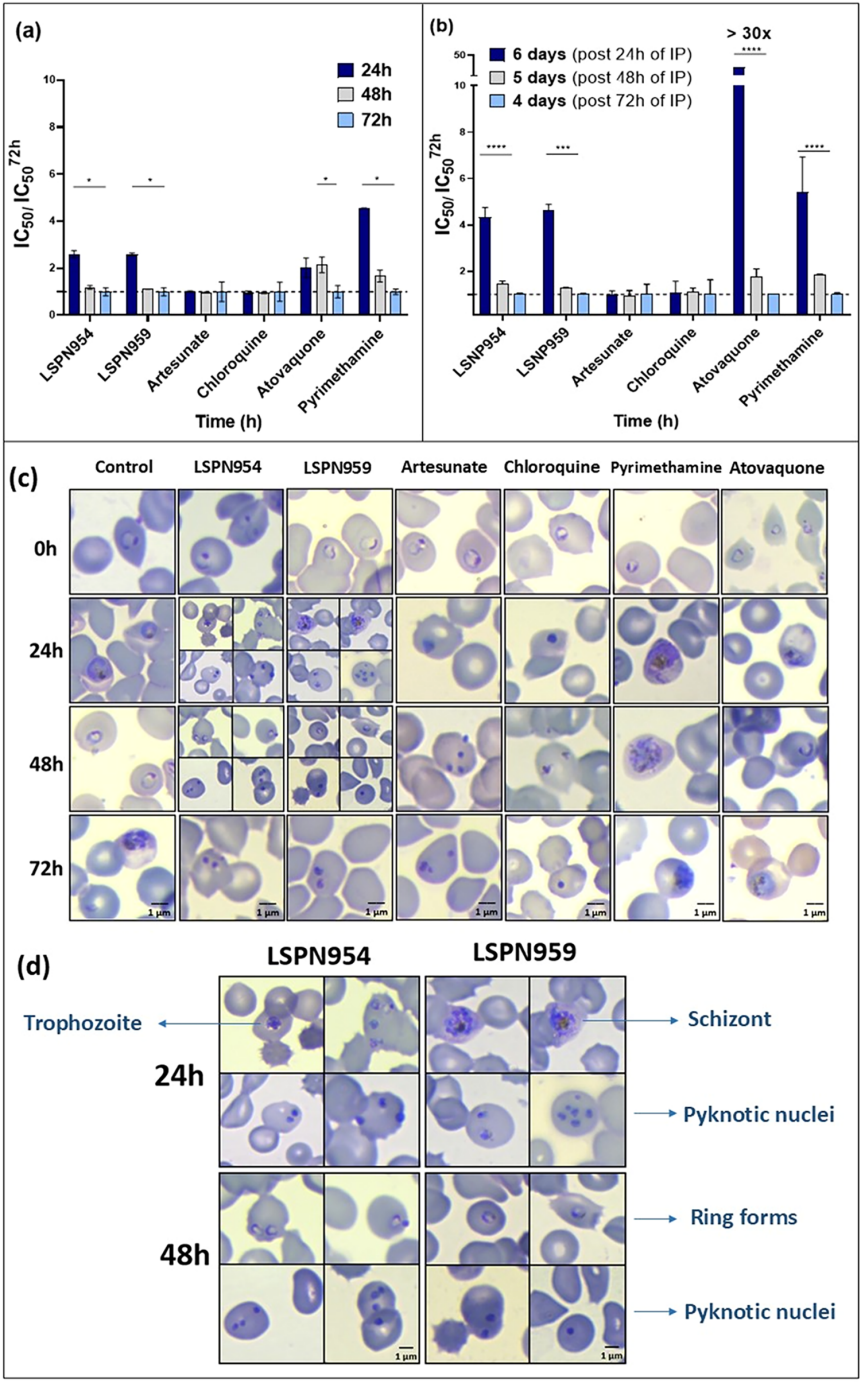
(a) Evaluation of the speed of action of the new indole derivatives **LSPN954** and **LSPN959**. The inhibitory activities of the compounds at each exposure time were determined and normalized relative to the IC_50_ value assessed at 72 h. (b) The inhibitory activities of the indole compounds at each exposure time after 4, 5, and 6 days of regrowth were determined and normalized relative to the IC_50_ value assessed on day six. Day 6 corresponds to the plate after 24 h of inhibitor pressure (IP), day 5 to the plate after 48 h of IP, and day 4 to the plate after 72 h of IP. Data are presented as mean values (±S.D.) from 3 independent experiments (*n* = 3). ****p < 0.001; *p < 0.05 (ANOVA). (c) Morphological evaluation of the parasite in the absence (control), in the presence of **LSPN954** and **LSPN959**, artesunate and chloroquine (fast-acting inhibitors), as well as atovaquone and pyrimethamine (slow-acting inhibitors). (d) Amplified images of parasite morphology at 24 and 48 h in the presence of **LSPN954** and **LSPN959**. Pyknotic nuclei and trophozoites and pyknotic nuclei and ring forms at 24 and 48 h are observed, respectively. (Scale bar = 1 μm).

In parallel with the quantitative evaluation of the speed of action, the morphological development profile of the parasites was observed at the three intervals of interest. In the negative control group (without inhibitor pressure), *P. falciparum* developed as expected, progressing from the ring stage at 0 h, to trophozoites at 24 h, and to ring and trophozoite forms at 48 and 72 h, respectively (Figure 1c). The fast-acting antimalarials artesunate and chloroquine inhibited parasite growth within the first hours of incubation, evidenced by the presence of pyknotic nuclei, indicating cell death (Figure 1c). In contrast, atovaquone and pyrimethamine, which exhibit a slow inhibition profile, allowed expected development during the initial hours, and morphological alterations became evident at 48 and 72 h. Regarding the compounds of interest, **LSPN954** and **LSPN959**, pyknotic nuclei and trophozoites were observed within the first 24 h, followed by ring forms at 48 h, indicating the parasites’ ability to recover (Figure 1d). These findings are consistent with quantitative data, suggesting that these antiplasmodial inhibitors exhibit a slow onset of action.

To confirm this observation, we conducted an extended protocol adapted from Huang et al.^30^ The protocol consists of evaluating the three aforementioned intervals (24, 48, and 72 h), followed by regrowth periods of 6, 5, and 4 days, respectively, after inhibitor removal. The fast-acting controls, artesunate and chloroquine, which eliminate the parasite within the first hours of exposure, exhibit consistent IC_50_ values over time, including the extended days after inhibitor withdrawal, with IC_50_^6d^/IC_50_^4d^ ratios closer to 1 (Figure 1b). In contrast, slow-acting controls, pyrimethamine and atovaquone exhibit greater IC_50_^6d^/IC_50_^4d^ values, of approximately 6- and >30-fold, respectively, followed by reductions in IC_50_^5d^/IC_50_^4d^ and IC_50_^4d^/IC_50_^4d^ (48 and 72 h of inhibitor pressure) (Figure 1b), thereby indicating efficacy at later stages following inhibitor exposure. Compounds **LSPN954** and **LSPN959** showed an increased IC_50_^6d^/IC_50_^4d^ ratios (4-fold), suggesting a slow-acting inhibitory activity (Figure 1b).

### Indole Derivatives Do Not Inhibit Apicoplast-Dependent Isoprenoid Biosynthesis

Given the slow-action profile of compounds **LSPN959** and **LSPN954**, we investigated whether their effects could be related to apicoplast-dependent processes, as inhibitors of this organelle often display a delayed death phenotype which can be reversed by isopentenyl pyrophosphate (IPP) supplementation.^31^ To explore this hypothesis, an IPP rescue assay was performed.^32^ Parasite growth in cultures treated with **LSPN959** or **LSPN954** was not restored in the presence of IPP (Figure 2), suggesting that their antiplasmodial activity is unlikely to result from inhibition of apicoplast-dependent isoprenoid biosynthesis (MEP pathway).

**Figure 2:**
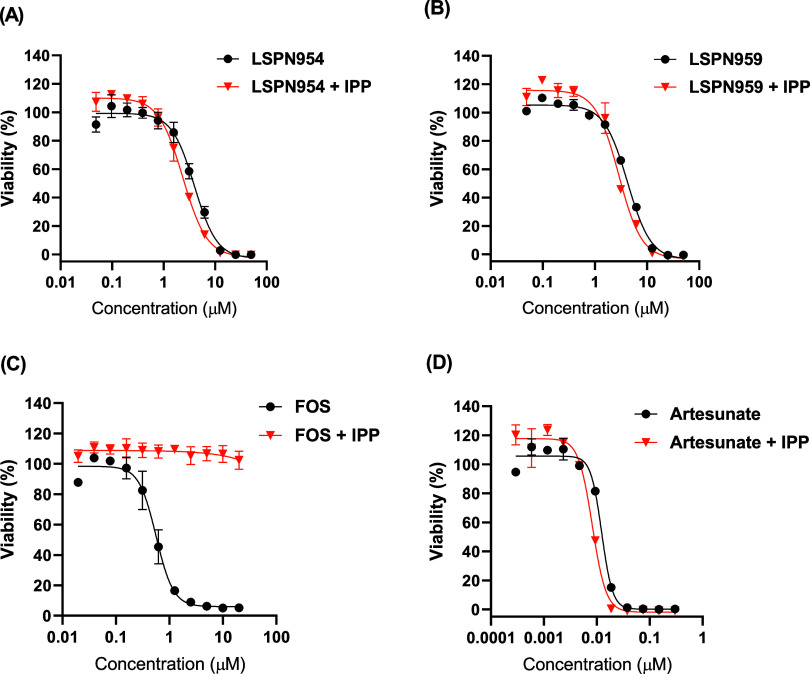
IPP chemical rescue assay in *P. falciparum* 3D7. Concentration–response curves for (A) **LSPN954**, (B) **LSPN959**, (C) fosmidomycin (FOS; positive rescue control), and (D) artesunate (negative rescue control), evaluated in the absence (black) and presence (orange) of IPP (200 μM). Cultures were incubated for 72 h, and IC_50_ values were determined using the SYBR Green I assay. IC_50_ + DP (μM), −IPP/+IPP: **LSPN954** = 3.1 ± 1.0/2.0 ± 0.5; **LSPN959** = 3.7 ± 0.7/2.4 ± 0.7; FOS = 1.4 ± 0.1/> 20; artesunate = 10.5 ± 2.1/7.0 ± 1.4. Data are presented as mean ± SD from at least two independent experiments.

### Indole Derivatives Exhibit an Additive Combination with Artesunate

Currently, malaria treatment primarily relies on artemisinin-based combination therapies (ACTs), which pair artemisinin derivatives with longer-acting antimalarials to enhance efficacy and minimize resistance risks.^6,7^ The slow-action profile of the new compounds prompted investigation into their potential use in combination with fast-acting antimalarials, such as artesunate.

The combination of **LSPN954** (Figure 3A) and **LSPN959** (Figure 3C) with artesunate exhibited an additive profile with the experimental data points (red dots and red region) close to the additivity curve (black line and gray region). The absence of a statistical difference between the assessed values and the additivity isobole supports the additive combination profiles (*p*-values of 0.0964 and 0.0968; Figure 3B,D, respectively). The additivity observed with artesunate suggests the absence of synergy/antagonism under the tested conditions and is compatible with predominantly independent effects; although it does not prove mechanistic independence, it also does not exclude partial pathway overlap, especially considering the kinetic difference between a fast-acting drug and slow-acting compounds.

**Figure 3:**
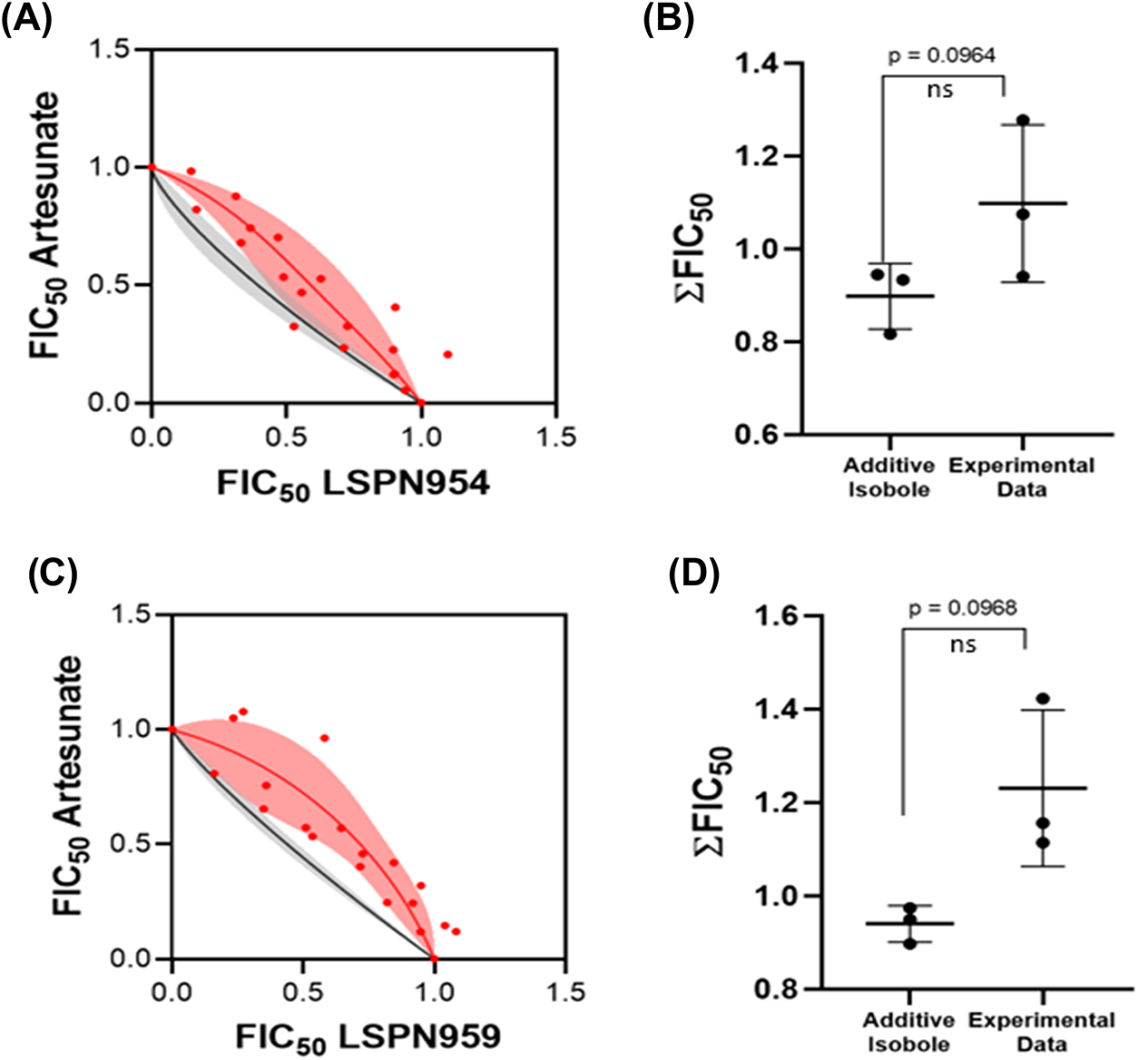
Evaluation of the combination of **LSPN954** and **LSPN959** with artesunate. The red region and red data points represent the experimental results, while the black line and gray region indicate the additivity curve. Isobolograms of **LSPN954** (A) and **LSPN959** (C) combined with artesunate. Statistical analysis for the combination of **LSPN954** (B) and **LSPN959** (D) with artesunate. The data represent the ∑FIC_50_ values of three independent experiments. Statistical analysis was carried out by using Student’s paired *t* test (*p*-value <0.05 indicates a statistical distinction between the experimental findings and the additivity isobole). ns: not significant.

### The New Indole Derivatives Exhibit Potency against Resistant Strains of *P. falciparum*

The next step in investigating the antiplasmodial properties of the indole derivatives was the assessment of their inhibitory activity against a representative panel of drug-resistant *P. falciparum* strains. The panel consisted of six *P. falciparum* strains known for their resistance to antimalarial inhibitors, including 3D7 (sensitive to all conventional antimalarials), the multidrug-resistant strains K1 (resistant to chloroquine, sulfadoxine, pyrimethamine, and cycloguanil) and Dd2 (resistant to chloroquine, sulfadoxine, pyrimethamine, mefloquine, and cycloguanil), TM90C6B (resistant to atovaquone), Dd2^R^_DSM265 (resistant to DSM265, a *Pf*DHODH inhibitor), and 3D7^R^_MMV848 (resistant to MMV692848, a phosphatidylinositol-4-kinase (PI4K) inhibitor). The resistance index (RI), defined as the ratio of the IC_50_ of the strain of interest to the IC_50_ of the 3D7 strain, was used to assess cross-resistance. A compound is considered cross-resistant to conventional antimalarials when the RI values are greater than 5.^33^ Both **LSPN954** and **LSPN959** inhibitors displayed RI values close to 1 against the resistant strains (Figure 4), indicating no cross-resistance with standard antimalarials.

**Figure 4:**
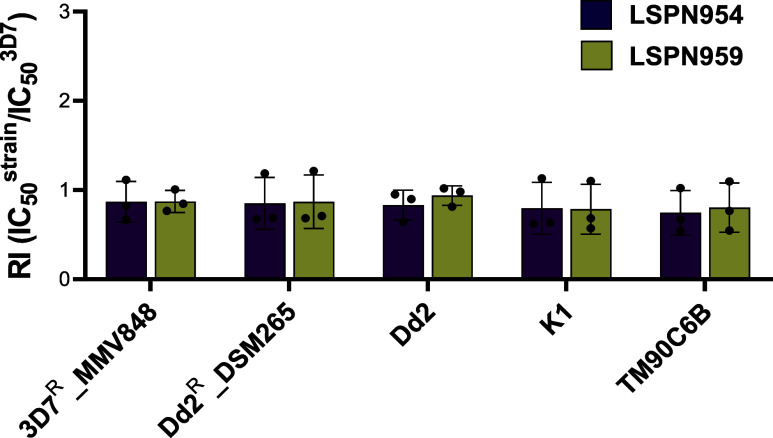
Resistance indexes of **LSPN954** and **LSPN959** against a representative panel of multidrug resistant *P. falciparum* strains (3D7^R^_MMV848, Dd2^R^_DSM265, Dd2, K1 and TM906CB) in relation to the sensitive 3D7 strain. IC_50_ values were calculated from three independent experiments and are presented as the mean ± standard deviation (SD). Resistance index (RI) values were calculated as the ratio of the IC_50_ between the resistant strain and the sensitive 3D7 strain (RI = IC_50_^Resistant strain^/IC_50_^3D7^). Dd2 has N86F mutation in *pfmdr1* gene; M74I, N75E, K76T mutations in *pfcrt* gene; and N51I, C59R, S108N mutations in *pfdhfr* gene; and S436F, A437G, A613S in *pfdhps* gene. K1 has N86Y mutation in *pfmdr1* gene; M74I, N75E, K76T mutations in *pfcrt* gene; C59R, S108N mutations in *pfdhfr* gene; and A437G, A581G in *pfdhps* gene. TM90C6B has the Y268S mutation in *pfcytb* gene; Y184F in *pfmdr1* gene; M74I, N75E, K76T mutations in *pfcrt* gene; N51I, C59R, S108N mutations in *pfdhfr* gene; and S436F, A437G, A581G in *pfdhps* gene. 3D7^R^_MMV848 has the V1357F mutation in *pfpi4k* gene. Dd2^R^_DSM265 has the F188L mutation in *pfdhodh* gene in addition to the mutations described for Dd2. The *pfmdr1* copy number is one for the wild-type strain 3D7 and K1, and three for Dd2.^34^

## Discussion

In this study, we synthesized and evaluated a new library of indole-based peptidomimetics with antiplasmodial activity against *P. falciparum*. Our findings highlight consistent structure–activity relationships and identify promising compounds as starting points for the development of antimalarial agents.^35,36^ The rational synthesis strategy of functionalized derivatives revealed diverse activity and toxicity profiles, underscoring the key role of structural modifications in determining the biological activity of this compound class. The SAR analysis revealed that aromatic and halogen substituents on the indole scaffold strongly influenced antiplasmodial activity. **LSPN954** and **LSPN959** were the most promising derivatives, with low micromolar potency against 3D7 strain (IC_50_ = 1.7 and 1.05 μM) and favorable selectivity indices (SI = 59 and 95), while maintaining low cytotoxicity against HepG2 and HEK293 cells (CC_50_ > 50 μM). A phenyl group at the R^2^ position proved essential for potency, although bulky substituents at the indole nitrogen could compensate for its absence. The introduction of a linker between the indole and the amide bond also enhanced the inhibitory activity. Additionally, halogenation with bromine substituent provided the best balance between potency and cytotoxicity. The most potent compounds (**LSPN954** and **LSPN959**) share physicochemical properties typical of the series (MW > 560 Da, logP > 6, TPSA ∼ 75 Å^2^, Table S1 and Figures S1–S6), suggesting that the inhibitory activity is not related with lipophilicity or size but rather with specific substituent patterns, such as halogenation and phenyl substitution at R_2_, which may influence their interaction with biological targets.

Biological assays suggested a slow-acting inhibition and additive interaction when combined with artesunate (Figures 1 and 3). This combination of different kinetic profiles is therapeutically advantageous,^37^ as artesunate rapidly targets early blood stages, while the slower-acting compounds may provide sustained activity or target later stages of the parasite life cycle.^33,38^

Given the slow-acting inhibition profile, we explored whether indole derivatives might affect the isoprenoid precursor biosynthesis pathway.^32^ Since supplementing with IPP did not reverse the growth inhibition caused by peptidomimetics (Figure 2), we determined that these compounds do not disrupt isoprenoid metabolism. The exact mechanism by which these peptidomimetics work remains undetermined. Notably, indole-based compounds are believed to be effective against various CQ-resistant strains due to their multiple mechanisms of action.^11^ Consistent with this, indole-based *P. falciparum* inhibitors have been reported to block PfATP4,^39^ hinder hemozoin formation,^40^ and modulate melatonin receptor activity.^41^

**LSPN954** and **LSPN959** demonstrated no cross-resistance across a representative panel of multidrug-resistant *P. falciparum* strains, including 3D7^R^_MMV848, Dd2^R^_DSM265, Dd2, K1, and TM90C6B, with resistance indices close to 1 (Figure 3). This observation suggests that these derivatives do not show cross-resistance with chloroquine, mefloquine, atovaquone and other standard antimalarials in the tested strains.

The combination of negligible cross-resistance and favorable selectivity highlights their promise as attractive scaffolds for further exploration and determination of the mechanism of action.^42^ Interestingly, a recent study on indole-2-carboxamides reported cross-resistance associated with efflux-mediated mechanisms, particularly involving *Pf*CRT.^19^ These findings highlight the particular interest of the LSPN series and their unique scaffold, as their lack of cross-resistance suggests a distinct resistance profile and supports their potential as promising candidates for antimalarial drug development.

Considering that *P. falciparum* has already developed resistance to all clinically used antimalarials,^43^ introducing new compounds with distinct mechanisms of action represents a crucial strategy to fight malaria and delay resistance development, particularly in Southeast Asia and parts of Africa.^44,45^

## Conclusions

We have synthesized and characterized a novel set of indole-based peptidomimetics with potent activity against *P. falciparum*. Systematic SAR analysis revealed that aromatic and halogen substituents, as well as modifications at the indole nitrogen and amide linkage, significantly influenced antiplasmodial potency and selectivity. **LSPN954** and **LSPN959** emerged as the most promising derivatives, combining low micromolar potency with high selectivity indices against human cells with low cytotoxic effect on liver cells. Importantly, both compounds retained activity across a representative panel of multidrug-resistant *P. falciparum* strains, suggesting they are not affected by resistance mechanisms present in these strains. In addition, their slow-acting profile may act by eliminating residual parasites from fast-acting compounds. Combination testing with artesunate yielded additive effects, indicating no enhancement over monotherapy under the conditions tested. Collectively, these findings establish indole peptidomimetics as an attractive and versatile candidates for the discovery of new leads compounds against malaria.

## Materials and Methods

### Chemistry

All commercially available reagents were purchased from Sigma-Aldrich. The synthesized products were purified by column chromatography or preparative thin layer chromatography using silica gel 60, 230–400 mesh. TLC was performed on silica gel 60 F254 supported in aluminum sheets. The ^1^H and ^13^C NMR spectra were recorded on a Bruker DRX 400 MHz spectrometer. The chemical shifts (δ) are given in ppm units and the coupling constants (*J*) in Hertz (Hz). The multiplicity of signs is expressed by the following abbreviations: s (singlet), brs (broad singlet), d (double), t (triplet), q (quartet), m (multiplet). Melting points were determined using a Büchi M-560 Basic Melting Point Apparatus. The HRMS data were acquired using a Shimadzu Nexera LC-30AD UHPLC equipped with QqTOF Bruker Daltonics Impact HD or in an Ultra-High Performance Liquid Chromatography-Electrospray Ionization Tandem Mass Spectrometry (UHPLC-ESI-MS/MS) using an Agilent 6545 LC/Q-TOF MS system.

Synthetic procedures and spectroscopic data for compounds **LSPN928–932**, **LSPN940–959**, **LSPN1139**, and **LSPN1148** (**4a**–**i**) are detailed in our previous work.^24^ Herein we report only spectroscopic data for **LSPN955** (**4j**), **LSPN959** (**4k**), **LSPN1148** (**4l**), and **LSPN1139** (**4m**), obtained following the experimental procedure described in the aforementioned publication. These compounds were purified by preparative chromatography (Hex/EtOAc 30–50%).

#### *N*-(2-(*tert*-Butylamino)-2-oxoethyl)-*N*-(2-(4-methoxyphenyl)-2-(1-(pyrrolidine-1-carbonyl)-1*H*-indole-2-yl)­ethyl)­benzamide (**4j**) (**LSPN955**)

The compund was obtained in 54% yield (0.03 g, 0.05 mmol) as a yellow solid, mp 117 °C–118 °C. ^1^H NMR (400 MHz, MeOD-*d*_4_, mixture of rotamers) δ 7.48 (d, *J* = 8.3 Hz, 1H), 7.30–7.23 (m, 5H), 7.12–6.94 (m, 9H), 6.77 (d, *J* = 8.3 Hz, 1H), 6.30 (s, 1H), 4.49 (dd, *J* = 14.3, 5.9 Hz, 1H), 4.36 (dd, *J* = 14.3, 5.9 Hz, 1H), 4.08–3.88 (m, 1H), 3.73 (d, *J* = 17.2 Hz, 1H), 3.41–3.26 (m, 1H), 3.24–3.11 (m, 1H), 2.86–2.6 (m, 1H), 1.96–1.87 (m, 1H), 1.86–1.75 (m, 1H), 1.74–1.52 (m, 1H), 1.43–1.33 (m, 1H), 1.14 (s, 9H), 1.01–0.69 (m, 1H). ^13^C NMR (100 MHz, MeOD-*d*_4_, mixture of rotamers) δ 175.1; 169.4; 160.8; 154.0; 141.3; 137.6; 136.8; 136.5; 136.3; 133.3; 132.6; 131.2; 130.9; 130.8; 129.7; 129.5; 128.1; 127.5; 124.3; 124.2; 122.3; 122.1; 115.4; 115.2; 111.5; 102.8; 55.9; 53.6; 50.9; 49.3; 47.9; 41.5; 29.0; 26.3; 25.5. DEPT135 (101 MHz, MeOD-*d*_4_, mixture of rotamers) δ 131.2; 130.8; 129.7; 129.5; 128.1; 127.5; 124.3; 124.2; 122.3; 122.1; 115.4; 115.2; 111.5; 102.8; 55.9; 53.6; 50.9; 49.3; 47.9; 41.5; 29.0; 26.2; 25.5. HRMS (ESI): *m*/*z* calculated for C_35_H_40_N_4_O_4_ [(M + H)]^+^: 581.3128, found: 581.3116.

#### *N*-(2-(4-Bromophenyl)-2-(1-(pyrrolidine-1-carbonyl)-1*H*-indole-2-yl)­ethyl)-*N*-(2-(*tert*-butylamino)-2-oxoethyl)­benzamide (**4k**) (**LSPN959**)

The compund was obtained in 42% yield (0.03 g, 0.05 mmol) as a yellow solid, mp 127 °C–128 °C. ^1^H NMR (400 MHz, MeOD-*d*_4_, mixture of rotamers) δ 7.53 (d, *J* = 7.8 Hz, 1H), 7.35 (d, *J* = 7.8 Hz, 1H), 7.31–7.20 (m, 8H), 7.04 (d, *J* = 6.7 Hz, 1H), 6.81 (d, *J* = 6.7 Hz, 1H), 6.72 (s, 1H), 6.34 (s, 1H, NH*)*, 4.86 (t, *J* = 6.5 Hz, 1H) 4.37 (dd, *J* = 13.2, 6.5 Hz, 1H), 4.08–4.04 (m, 1H), 3.76–3.67 (m, 1H), 3.45–3.28 (m, 1H), 3.16–3.08 (m, 1H), 2.91–2.77 (m, 2H), 1.88–1.83 (m, 1H), 1.67–1.61 (m, 1H), 1.57–1.53 (m, 1H), 1.45–1.40 (m, 2H), 1.13 (s, 9H), 0.95–0.74 (m, 1H). ^13^C NMR (100 MHz, MeOD-*d*_4_, mixture of rotamers) δ 173.7, 167.9, 152.6, 140.1, 139.4, 136.1, 135.0, 132.3, 129.3, 129.2, 128.7; 128.4; 128.2; 128.0; 127.9; 127.4; 126,.6; 125.9; 122.8; 120.8; 120.7; 113.1; 110.0; 101.6; 52.0; 50.8; 49.2; 48.2; 47.9; 40.8; 27.5; 27.4; 24.7. DEPT135 (100 MHz, MeOD-*d*_4_, mixture of rotamers) δ132.3 (CH); 129.3 (CH); 129.2; 128.7 (CH); 128.4 (CH); 128.2 (CH); 127.9 (CH); 127.4 (CH); 126.6 (CH); 125.9 (CH); 122.8 (CH); 120.8 (CH); 110.0 (CH); 101.6 (CH); 52.0 (CH_2_); 49.2 (CH_2_); 48.2 (CH_2_); 47.9 (CH_2_); 40.8 (CH); 27.5 (CH_2_); 27.4 (CH_2_); 24.7 (CH_2_). HRMS (ESI): *m*/*z* calculated for C_34_H_37_BrN_4_O_3_ [(M + H)]^+^: 628.2049; found: 629.2128.

#### *N*-(2-(*tert*-Butylamino)-2-oxoethyl)-*N*-(2-(1-(pyrrolidine-1-carbonyl)-1*H*-indole-2-yl)-2-(o-toyl)­ethyl)­benzamide (**4m**) (**LSPN1139**)

The compund was obtained in 39% yield (0.02 g, 0.02 mmol) as a yellow solid, mp 126–129 °C. ^1^H NMR (400 MHz, MeOD-*d*_4_, mixture of rotamers) δ 7.63 (d, *J* = 7.4 Hz, 1H), 7.41–7.36 (m, 6H), 7.25 (d, *J* = 7.5 Hz), 7.21–7.11 (m, 3H), 7.08 (d, *J* = 6.4 Hz, 1H), 6.93–6.85 (m, 1H), 6.59 (s, 1H), 5.29 (t, *J* = 7.5 Hz, 1H), 4.69 (dd, *J* = 13.2, 7.5 Hz, 1H), 4.18–4.09 (m, 1H), 3.87 (d, *J* = 17.2 Hz, 1H), 3.81–3.76 (m, 1H); 3.54 (d, *J* = 17.2 Hz, 1H), 3.45–3.37 (m, 1H), 3.27–3.15 (m, 1H), 3.01–2.91 (m, 1H), 2.52 (s, 3H), 2.40–2.23 (m, 1H), 2.08–2.04 (m, 1H), 1.77–1.73 (m, 1H), 1.53–1.50 (m, 1H), 1.25 (s, 9H), 0.92 (m, 1H). ^13^C NMR (100 MHz, MeOD-*d*_4_, mixture of rotamers) δ 173.9, 167.9, 152.6, 139.9, 138.5, 136.9, 136.1, 135.1, 130.5, 130.4, 129.4, 129.3, 128.6, 128.3, 128.0, 127.9, 127.7, 127.0, 126.7, 126.5, 126.2, 125.9, 122.8, 122.7, 120.8, 120.6, 109.9, 102.5, 51.8, 50.8, 49.3, 47.6; 46.5; 36.8; 27.4; 24.8; 23.9; 18.4. DEPT135 (100 MHz, MeOD-*d*_4_, mixture of rotamers) δ 130.4 (CH); 129.5 (CH); 129.3 (CH); 128.3 (CH); 128.0 (CH); 127.0 (CH); 126.5 (CH); 126.2 (CH); 125.9 (CH); 122.7 (CH); 120.8 (CH); 120.6 (CH); 109.9 (CH); 102.5 (CH); 51.8 (CH_2_); 49.3 (CH_2_); 47.6 (CH_2_); 46.5 (CH_2_); 36.8 (CH); 27.4 (CH); 24.8 (CH_2_); 23.9 (CH_2_); 18.4 (CH). HRMS (ESI): *m*/*z* calculated for C_35_H_40_N_4_O_3_ [(M + H)]^+^: 565.3173, found: 565.3176.

#### *N*-(2-(*tert*-Butylamino)-2-oxoethyl)-*N*-(2-(4-methoxyphenyl)-2-(5-methyl-1-(pyrrolidine-1-carbonyl)-1*H*-indole-2-yl)­ethyl)­benzamide (**4l**) (**LSPN1148**)

The compund was obtained in 43% yield (0.026 g, 0.03 mmol) as a yellow solid, mp 115 °C–117 °C. ^1^H NMR (400 MHz, MeOD-*d*_4_, mixture of rotamers) δ 7.45–7.29 (m, 6H), 7.25–7.08 (m, 2H), 7.05–6.96 (m, 1H), 6.95–6.84 (m, 1H), 6.88–6.71 (m, 1H), 6.69 (s, 1H), 6.32 (s, 1H), 4.60–4.56 (m, 1H), 4.22–3.90 (m, 1H), 3.77 (s, 3H), 3.55–3.36 (m, 1H), 3.19–2.88 (m, 1H), 2.41 (s, 3H), 2.10–1.9 (m, 2H), 2.02–1.85 (m, 2H), 1.76–1.72 (m, 2H), 1.60–1.56 (m, 2H), 1.25 (s, 9H), 1.10–0.79 (m, 1H). ^13^C NMR (100 MHz, MeOD-*d*_4_, mixture of rotamers) δ 175.0, 169.3, 160.7, 154.2, 141.2, 137.5, 134.8, 133.3, 131.6, 131.4, 131.0, 130.7, 129.6, 129.4; 128.3; 127.4; 126.6; 125.6; 125.4; 121.8; 115.2; 115.0; 111.1; 102.4; 55.8; 53.3; 50.7; 49.6; 48.8; 47.8; 41.4; 28.9; 26.1; 25.4; 21.4. DEPT135 (101 MHz, MeOD-*d*_4_, mixture of rotamers) δ 131.0 (CH); 129.6 (CH); 127.4 (CH); 125.4 (CH); 121.8 (CH); 115.0 (CH); 111.1 (CH); 102.4 (CH); 55.8 (CH); 53.3 (CH_2_); 49.6 (CH_2_); 48.8 (CH_2_); 47.8 (CH_2_); 41.4 (CH); 28.9 (CH); 26.1 (CH_2_); 25.4 (CH_2_); 21.4 (CH). HRMS (ESI): *m*/*z* calculated for C_36_H_42_N_4_O_4_[(M + H)]^+^: 595.3295, found: 595.3295.

### Synthesis of Compounds **6a**–**d**, **6g**, **9a**,**d**, and **12a**,**c** ^46^

In a sealed tube, a mixture of carboxylic acid (0.5 mmol), amine (0.5 mmol), paraformaldehyde (0.5 mmol, 0.015 g) or benzaldehyde (0.5 mmol, 51 μL) and *t*-butyl (0.5 mmol, 56.5 μL) or *n*-butyl isocyanide (0.5 mmol, 53 μL) in MeOH (1 mL) was stirred at r.t. for 24 h. Then, the reaction mixture was concentrated in vacuum and the residue purified by chromatographic column or preparative chromatography (Hex/EtOAc 40%).

#### *N*-(2-(*tert*-Butylamino)-2-oxoethyl)-*N*-phenyl-1*H*-indole-2-carboxamide (**6a**) (**LSPN1143**)^46^

Synthesized from aniline (45.6 μL), paraformaldehyde and *t*-butyl isocyande, obtained as a slightly yellowish solid in 87% yield (0.127 g). mp 206 °C–208 °C. ^1^H NMR (400 MHz, CDCl_3_) δ 9.44 (s, 1H, *NH*), 7.47–7.34 (m, 3H), 7.32–7.27 (m, 4H), 7.18–7.14 (m, 1H), 6.94 (t, *J* = 7.5 Hz, 1H), 6.26 (s, 1H), 5.24 (s, 1H, *NH*), 4.34 (s, 2H), 1.29 (s, 9H). ^13^C NMR (100 MHz, CDCl_3_): δ 167.8, 162.7, 142.8, 135.5, 130.1, 129.0, 128.6, 128.4, 127.6, 125.0, 122.4, 120.5, 111.6, 108.2, 56.5, 51.4; 28.8.

#### *N*-(2-(*tert*-Buthylamine)-2-oxo-1-phenylethyl)-*N*-phenyl-1*H*-indol-2-carboxamide (**6b**) (**LSPN1141**)^47^

Synthesized from aniline (45.6 μL), benzaldehyde and *t*-butyl isocyande, obtained as a white solid in 64% yield (0.136 g). mp 204.5 °C–206.8 °C. ^1^H NMR (400 MHz, CDCl_3_, presence of rotamers) δ 9.28 (s, 1H, *NH*), 7.27–7.10 (m, 13H), 6.92–6.88 (m, 1H), 6.04 (s, 1H), 5.72 (s, 1H), 1.27 (s, 9H). ^13^C NMR (100 MHz, CDCl_3_, presence of rotamers) δ 168.6, 162.3, 140.0, 135.3, 134.5, 131.2, 130.4, 130.2, 130.0, 129.3, 129.0, 128.7, 128.5, 128.3, 127.7, 127.0, 124.7, 122.4, 120.1, 111.5, 107.7; 67,2; 51,7; 28.7.

#### *N*-(2-(*tert*-Butylamino)-2-oxo-1-phenylethyl)-*N*-(2-iodophenyl)-1*H*-indole-2-carboxamide (**6c**) (**LSPN1142**)^47^

Synthesized from 2-iodoaniline (0.109 g), benzaldehyde, and *t*-butyl isocyanide, obtained as a yellow solid in 52% yield (0.143 g). ^1^H NMR (400 MHz, CDCl_3_, presence of rotamers): δ 9.65 (s, 1H), 8.13 (d, *J* = 7.8 Hz, 1H), 7.62 (d, *J* = 7.8 Hz, 1H), 7.43 (d, *J* = 6.8 Hz, 1H), 7.40–7.36 (m, 4H), 7.34 (d, *J* = 7.9 Hz, 1H), 7.22–7.12 (m, 5H), 7.3–6.97 (m, 2H), 6.21 (s, 1H), 5.89 (s, 1H, *NH*), 5.11 (s, 1H,), 1.32 (s, 9H). ^13^C NMR (100 MHz, CDCl_3_, presence of rotamers) δ 169.0; 162.4; 141.9; 140.3; 135.9; 133.4; 132.4; 131.5; 130.4; 129.9; 129.8; 129.6; 129.3; 128.9; 128.8; 127.5; 124.8; 122.5; 121.2; 120.2; 112.4; 111.8; 109.6; 107.5; 66.9; 51.7; 28.8.

#### *N*-(2-(Butylamino)-2-oxoethyl)-*N*-phenyl-1*H*-indol-2-carboxamide (**6d**) (**LSPN1151**)

Synthesized from aniline, paraformaldehyde and *n*-butyl isocyanide, obtained as a slightly yellowish solid in 94% yield (0.16 g). mp 159 °C–161 °C. ^1^H NMR (400 MHz, DMSO-*d*_6_) δ 11.59 (s, 1H, *NH*), 7.97 (t, J = 5.6 Hz, 1H), 7.50–7.42 (m, 5H), 7.39 (d, *J* = 8.0, Hz, 1H), 7.28 (d, *J* = 8.0 Hz, 1H), 7.12 (t, *J* = 7.9 Hz, 1H), 6.90 (t, *J* = 7.9 Hz, 1H), 5.21 (s, 1H, *NH*), 4.38 (s, 2H), 3.08 (dd, *J* = 12.7, 6, 8 Hz, 2H), 1.42–1.33 (m, 2H), 1.29–1.19 (m, 3H), 0.85 (t, *J* = 7.3 Hz, 4H). ^13^C NMR (100 MHz, DMSO-*d*_6_) δ 167.9; 161.9; 143.9, 136.0; 130.2; 130.0; 129.0; 128.7; 127.2; 124.0; 121.9; 120.1; 112.6; 106.3; 53.8; 38.7; 31.7; 19.9; 14.1. HRMS (ESI): *m*/*z* calculated for C_21_H_23_N_3_O_2_ [(M + H)]^+^: 350.1863, found: 350.1859.

#### *N*-(2-(Butylamino)-2-oxoethyl)-*N*-pheny-1*H*-indol-2-carboxamide (**6g**) (**LSPN1149**)

Synthesized from carboxylic acid **6**, benzaldehyde and *t*-butyl isocyanide, obtained as a yellowish solid in 82% yield (0.036 g). mp 139.1 °C–140.8 °C. ^1^H NMR (400 MHz, CDCl_3_): δ 9.27 (s, 1H), 7.39 (d, *J* = 7.7 Hz, 1H), 7.28 (d, *J* = 7.7 Hz, 1H), 7.18–7.05 (m, 8H), 7.01 (d, *J* = 7.6 Hz, 3H), 6.96 (t, *J* = 7.4 Hz, 1H), 5.88 (s, 1H, *NH*), 5.45 (s, 1H), 3.55 (s, 2H), 1.26 (s, 9H). ^13^C NMR (100 MHz, CDCl_3_): δ 170.7; 168.7; 139.8; 136.3; 134.4; 132.2; 130.3; 129.0; 128.5; 128.5; 128.4; 128.2; 121.2; 119.8; 119.4; 110.9; 100.8; 66.2; 51.8; 34.2; 28.7.

#### *N*-(1*H*-Indol-2-yl)­methyl-*N*-(2-(*tert*-butylamino)-2-oxoethyl)­benzamide (**9a**) (**LSPN1152**)

Synthesized from amine **8** (0.07 g, 0.5 mmol), paraformaldehyde, benzoic acid (0.06 g) and *t*-butyl isocyanide (56 μL, 0.5 mmol), obtained as a yellowish solid in 91% yield (0.17 g). ^1^H NMR (400 MHz, DMSO-*d*_6_, mixture of rotamers): δ 11.08 (s, 1H, *NH*), 7.61–7.34 (m, 7H), 7.07 (d, *J* = 8. Nine Hz, 2H), 6.32 (s, 1H), 4.79 (s, 2H), 3.79 (s, 2H), 1.24 (s, 9H). ^13^C NMR (101 MHz, DMSO-*d*_6_, mixture of rotamers). δ 171.8; 167.7; 136.9; 136.8; 135.2; 135.1; 133.3; 130.0; 129.9; 129.0; 128.9; 128.7; 127.0; 126.8; 121.4; 119.5; 111.7; 101.4; 51.3; 50.9; 43.3; 28.8. HRMS (ESI): *m*/*z* calculated for C_22_H_25_N_3_O_2_ [(M + H)]^+^: 364.2020, found: 364.2016.

#### *N*-(1*H*-Indol-2-yl)­methyl-*N*-(2-(butylamino)-2-oxoethyl)­benzamide (**9d**) (**LSPN1156**)

Synthesized from amine **8** (0.07 g, 0.5 mmol), paraformaldehyde, benzoic acid (0.06 g) and *n*-butyl isocyanide, obtained as a yellowish solid in 89% yield (0.17 g). ^1^H NMR (400 MHz, DMSO-*d*_6_, mixture of rotamers): δ 11.0 (s, 1H, *NH*), 7.85–7.83 (m, 1H), 7.54–7.49 (m, 1H), 7.46–7.43 (m, 4H), 7.40–7.30 (m, 2H), 7.05 (t, *J* = 8.1 Hz, 1H), 6.36 (s, 1H), 4.77 (s, 2H), 3.74 (s, 2H), 3.10–3.02 (m, 4H), 1.28–1.15 (m, 4H), 0. 85 (t, *J* = 7.1 Hz, 3H). ^13^C NMR (101 MHz, DMSO-*d*_6_, mixture of rotamers) δ 171.8; 168.0; 136.9; 136.6; 136.3; 135.3; 135.0; 130.1; 129.9; 128.8; 128.3; 127.1; 121.4; 120.1; 119.5; 111.8; 101.4; 51.0; 43.0; 31.7; 31.5; 19.9; 14.0. HRMS (ESI): *m*/*z* calculated for C_22_H_25_N_3_O_2_ [(M + H)]^+^: 364.2020, found: 364.2025.

#### *N*-(2-(1*H*-Indol-3-yl)-2-phenylethyl)-*N*-(2-(*tert*-butylamino)-2-oxoethyl)­benzamide (**12a**) (**LSPN1157**)

Synthesized from amine **11a** (0.12 g, 0.5 mmol), paraformaldehyde and *t*-butyl isocyanide, obtained as a white solid, p. f. 118.7 °C–119.8 °C, in 82% yield (0.19 g). ^1^H NMR (400 MHz, DMSO-*d*_6_, presence of rotamers) δ 11.0 (s, 1H, *NH*), 7.61–7.53 (m, 1H), 7.6 (d, *J* = 7.4 Hz, 1H), 7.40 (s, 1H), 7.31–7.28 (m, 6H), 7.26 – 7.16 (m, 2H), 7.12–6.96 (m, 3H), 6.92 (t, *J* = 7.4 Hz, 1H), 4.76 (t, *J* = 7.9 Hz, 1H), 4.14 (dd, *J* = 13.2, 6.9 Hz, 1H), 4.0–3.9 (m, 2H), 3.62 (d, *J* = 16.7 Hz, 1H), 1.27 (m, 9H). ^13^C NMR (101 MHz, DMSO-*d*_6_, presence of rotamers) δ 171.9; 167.7; 143.6; 137.5; 136.9; 136.8; 133.2; 130.0; 129.7; 129.4; 129.0; 128.7; 128.6; 127.2; 127.0; 126.9; 126.8; 126.5; 122.5; 121.6; 119.0; 118.8; 115.9; 115.3; 111.9; 52.5; 50.8; 50.7; 29.0. DEPT135 (101 MHz, DMSO-*d*_6_, presence of rotamers) δ 133.2 (CH); 130.0 (CH); 129.7 (CH); 129.4 (CH); 129.0 (CH); 128.7 (CH); 128.6 (CH); 127.0 (CH), 126.8 (CH); 126.5 (CH); 122.5 (CH); 121.6 (CH); 119.0 (CH); 118.9 (CH); 111.9 (CH); 52.5 (CH_2_); 48.0 (CH_2_); 41.4 (CH); 29.0 (CH). HRMS (ESI): *m*/*z* calculated for C_29_H_31_N_3_O_2_ [(M + H)]^+^: 454.2489, found: 454.2497.

#### *N*-(2-(*tert*-Butylamino)-2-oxoethyl)-*N*-(2-phenyl-2-(1-tosyl-1*H*-indol-3-yl)­ethyl)­benzamide (**12c**) (**LSPN1158**)

Synthesized from **12a** (0.068 g), obtained as a white solid in 71% yield (0.064 g). mp 106.3 °C–108.5 °C. ^1^H NMR (400 MHz, DMSO-*d*_6_, presence of rotamers) δ 7.91 (d, *J* = 6.4 Hz, 1H), 7.41 (d, *J* = 6.4 Hz, 1H), 7.38–7.34 (m, 4H), 7.32–7.27 (m, 6H), 7.22–7.14 (m, 3H), 7.06 (d, *J* = 7.1 Hz, 3H), 4.72 (t, *J* = 7.4 Hz, 1H), 4.36 (dd, *J* = 13.4, 7.4 Hz, 1H), 3.80 (dd, *J* = 13.4, 7.4 Hz, 1H), 3.60 (d, *J* = 17.1 Hz, 1H), 2.29 (s, 3H), 1.15 (s, 9H). ^13^C NMR (100 MHz, DMSO-*d*_6_, presence of rotamers) δ 172.0; 167.7; 145.7; 141.7; 137.2; 134.9; 134.5; 130.6; 129.6; 128.9; 128.7; 128.6; 127.3; 127.2; 126.6; 125.4; 124.0; 123.7; 120.5; 113.8; 52.8; 50.7; 40.2; 28.8; 21.5. DEPT135 (100 MHz, DMSO-*d*_6_, presence of rotamers) δ 130.6 (CH); 129.6 (CH); 128.9 (CH); 128.7 (CH); 128.6 (CH); 127.3 (CH); 127.2 (CH); 126.6 (CH); 125.4 (CH); 123.7 (CH); 120.5 (CH); 113.8 (CH); 52.8 (CH_2_); 50.7 (CH_2_); 40.2 (CH); 28.8 (CH); 21.5 (CH). HRMS (ESI): *m*/*z* calculated for C_36_H_37_N_3_O_4_ [(M + H)]^+^: 608.2578, found: 608.2595.

### Synthesis of Compounds **6e** and **9b**

A suspension of the appropriate compound (0.15 mmol), DMF and Cs_2_CO_3_ (0.08 g, 0.23 mmol) was heated under reflux for 24 h. After this period, the reaction crude was concentrated in vacuum and subsequently extracted with EtOAc (3 × 10 mL). The organic layer was dried in anhydrous Na_2_SO_4_, concentrated in vacuum and the residue purified by preparative chromatography (Hex/EtOAc 20%).

#### 1-Benzyl-*N*-(2-(*tert*-butylamino)-2-oxoethyl)-*N*-phenyl-1*H*-indole-2-carboxamide (**6e**) (**LSPN1146**)

Synthesized from **6a** (0.052 g) and obtained as a yellow solid in 68% yield (0.045 g). mp 138 °C–139.0 °C. ^1^H NMR (400 MHz, CDCl_3_, mixture of rotamers*)* δ 7.55–7.35 (m, 3H), 7.34–7.23 (m, 4H), 7.22–7.13 (m, 4H), 7.1 (d, *J* = 0.8 Hz, 1H), 7.05–7.02 (m, 5H); 6.87–6.84 (m, 2H), 6.18 (s, 1H, *NH*), 6.13 (s, 1H), 5.75 (s, 2H), 4.33 (s, 2H), 1.26 (s, 9H). ^13^C NMR (101 MHz, CDCl_3_, mixture of rotamers) δ 167.6; 164.3; 143.8; 138.5; 138.0; 134.9; 130.6; 129.7; 129.4; 128.7; 128.2; 127.9; 127.5; 127.4; 126.9, 126.8; 126.7; 125.9; 124.4; 122.2, 120.4; 116.6, 109.9; 55.7; 47.4; 29.7. DEPT135 (101 MHz, CDCl_3_, mixture of rotamers) δ 129.4 (CH); 128.7 (CH); 127.9 (CH); 127.5 (CH); 126.9 (CH); 126.7 (CH); 124.4 (CH); 122.2 (CH); 120.4 (CH); 109.9 (CH); 55.7 (CH_2_); 47.3 (CH_2_); 28.7 (CH). HRMS (ESI): *m*/*z* calculated for C_28_H_29_N_3_O_2_ [(M + H)]^+^: 440.2333, found: 440.2339.

#### *N*-(1-Benzyl-1*H*-indol-2-yl)­methyl-*N*-(2-(*tert*-butylamino)-2-oxoethyl)­benzamide (**9b**) (**LSPN1153**)

Synthesized from **9a** (0.054 g) and obtained as a yellow solid in 45% yield (0.03 g). mp 158 °C–160 °C. ^1^H NMR (400 MHz, DMSO-*d*_6_, presence of rotamers) δ 7.58 (d, *J* = 7.1 Hz, 1H), 7.41–7.30 (m, 2H), 7.27–7, 25 (bs, 5H), 7.14–7.01 (bs, 2H), 7.08–7.02 (m, 1H), 6.89 (d, *J* = 7.1 Hz, 3H), 6.54 (s, 1H), 5.53 (s, 2H), 4.87 (s, 2H), 3.55 (s, 2H), 1.23 (s, 9H). ^13^C NMR (101 MHz, DMSO-*d*_6_, presence of rotamers) δ 174.4; 169.2; 139.8; 139.5; 136.9; 135.3; 129.8; 128.9; 128.1; 127.9; 127.6; 126.7; 126.5; 126.0; 125.7; 125.4; 123.3, 121.9; 120.2; 119.6; 109.8; 105.9; 52.2; 50.9; 47.4; 42.2; 28.8. DEPT135 (101 MHz, MeOD-*d*_4_) δ 129.3 (CH), 128.9 (CH), 128.1 (CH), 127.8 (CH), 127.4 (CH), 126.7 (CH), 123.3 (CH), 121.5 (CH), 120.9 (CH), 111.1 (CH), 105.8 (CH), 47.5 (CH_2_), 46.0 (CH_2_), 42.2 (CH_2_), 28.8 (CH). HRMS (ESI): *m*/*z* calculated for C_29_H_31_N_3_O_2_ [(M + H)]^+^: 454.2489, found: 454.2495.

### Synthesis of Compounds **6f**, **9c**–**e**, **12b**, and **12d** ^48^

NaH (60% in mineral oil, 0.006 g, 0.23 mmol) was added, in portions, to a solution of **6a**, **9a**, or **12a** (0.15 mmol) in anhydrous DMF (0.5 mL) in a 10 mL flask under ice bath and purged with N_2_. The mixture was stirred at r.t. for 1 h. After cooling to 0 °C again, a solution of TsCl (0.04 g, 0.23 mmol) or Boc_2_O (0.05 g, 0.23 mmol) in DMF was added and the mixture was left stirring at room temperature for 24 h. After this period, saturated solution of NH_4_Cl (2 × 20 mL) was added and extracted with EtOAc (3 × 5 mL). The combined organic layer was washed with saturated solution of NaCl (5 mL), dried with anhydrous Na_2_SO_4_, concentrated in vacuum and purified by preparative chromatography (Hex/EtOAc 30–50%).

#### *N*-(2-(*tert*-Butylamino)-2-oxoethyl)-*N*-phenyl-1-tosyl-1*H*-indol-2-carboxamide (**6f**) (**LSPN1150**)

Synthesized from **6a** (0.052 g), obtained as a slightly yellowish solid in 55% yield (0.04 g). mp 153 °C–156 °C. ^1^H NMR (400 MHz, DMSO-*d*_6_, presence of rotamers) δ 8.2 (d, *J* = 7.8 Hz, 1H), 7.80 (d, *J* = 8.3 Hz, 1H), 7.55 (bs, 1H), 7.47–7.38 (m, 9H), 7.31–7.27 (m, 1H), 7.18–7.10 (m, 1H), 6.91 (t, *J* = 7.8 Hz, 1H), 6.60 (s, 1H), 4.42 (bs, 2H), 2.35 (s, 3H), 1.28 (s, 9H). ^13^C NMR (101 MHz, DMSO-*d*_6_, presence of rotamers) δ 167.3; 162.6; 146.1; 144.1; 143.4; 136.0; 134.9; 130.4; 130.3; 129.9; 129.6; 129.0; 128.6; 128.0; 127.8; 126.0; 124.2; 122.6; 121.9; 120.1; 114.1; 112.6; 106.1; 54.1; 50.9; 50.7, 28.9; 21.5. HRMS (ESI): *m*/*z* calculated for C_28_H_29_N_3_O_4_S [(M + H)]^+^: 504.1951, found: 504.1949.

#### *N*-(2-(*tert*-Butylamino)-2-oxoethyl)-*N*-((1-tosyl-1*H*-indol-2-yl)­methyl)­benzamide (**9c**) (**LSPN1154**)

Synthesized from **9a** (0.054 g) and obtained as a white solid in 41% yield (0.03 g). mp 176 °C–178 °C. ^1^H NMR (400 MHz, DMSO-*d*_6_, presence of rotamers) δ 7.96 (d, *J* = 7.8 Hz, 1H), 7.77–7.72 (m, 2H), 7.69–7.65 (m, 3H), 7.62–7.52 (m, 3H), 7.49 (t, *J* = 7.8 Hz, 2H), 7.44–7.42 (m, 2H), 6.91 (s, 1H), 5.19 (s, 2H), 4.03 (s, 1H), 2.54 (s, 3H), 1.39 (s, 9H). ^13^C NMR (101 MHz, DMSO-*d*_6_, presence of rotamers) δ 171.9; 167.5; 146.0; 137.3; 136.9; 136.4; 136.1; 135.0; 130.8; 130.3; 129.7; 129.5; 128.9; 128.2; 127.2; 126.7; 126.5; 126.3; 125.2; 124.3; 121.6; 114.4; 109.7; 52.5; 50.8; 45.1; 29.0; 21.5. HRMS (ESI): *m*/*z* calculated for C_29_H_31_N_3_O_4_S [(M + H)]^+^: 518.2108, found: 518.2109.

#### *tert*-Butyl-2-((*N*-(2-(*tert*-butylamino)-2-oxoethyl)­benzamido)­methyl)-1*H*-indol-1-carboxylate (**9e**) (**LSPN1155**)

Synthesized from **9a** (0.054 g) and Boc_2_O (0.07 g), obtained as a sticky light brown solid in 37% (0.02 g) yield. ^1^H NMR (400 MHz, DMSO-*d*_6_, presence of rotamers) δ 8.04 (d, *J* = 7.8 Hz, 1H), 7.61–7.66 (m, 1H), 7.51 (d, *J* = 7.8 Hz, 3H), 7.46–7.41 (m, 3H), 7.29–7.20 (m, 1H), 6.56 (s, 1H), 4.91 (s, 2H), 3.83 (s, 2H), 1.66 (s, 9H), 1.24 (s, 9H). ^13^C NMR (101 MHz, DMSO-*d*_6_, presence of rotamers) δ 171.9; 167.6; 150.2; 137.9; 137.0; 136.8; 136.7; 130.2, 129.9; 129.1;129.0; 128.8; 128.6; 127.2; 126.7; 124.4; 123.4; 123.3; 120.9; 120.7; 115.5; 107.3; 84.9; 52.5; 50.8; 46.0, 29.0. DEPT135 (100 MHz, DMSO-*d*_6_, presence of rotamers) δ 130.2 (CH); 128.8 (CH); 127.2 (CH); 126.7 (CH); 124.4 (CH); 123.4 (CH); 123.3 (CH); 120.9 (CH), 115.5 (CH); 107.3 (CH); 52.5 (CH_2_); 46.0 (CH_2_); 29.0 (CH). HRMS (ESI): *m*/*z* calculated for C_27_H_34_N_3_O_4_ [(M + H)]^+^: 464.2544, found: 464.2545.

#### *N*-(2-(1*H*-Indol-3-yl)-2-(*p*-tolyl)­ethyl)-*N*-(2-(*tert*-butylamino)-2-oxoethyl)­benzamide (**12b**) (**LSPN1159**)

Synthesized from the amine **11b** (0.13 g, 0.5 mmol), obtained as a white solid in 65% yield (0.14 g). mp 106.3–108.5 °C. ^1^H NMR (400 MHz, DMSO-*d*_6_, mixture of rotamers): δ 10.93 (s, 1H, *NH*), 7.46–7.38 (m, 4H), 7.38–7.30 (m, 2H), 7.28 (d, *J* = 7.3 Hz, 3H), 7.09–7.01 (m, 1H), 6.97 (d, *J* = 8.3 Hz, 2H), 6.91 (t, *J* = 8.2 Hz, 2H), 4.71 (t, *J* = 7.0 Hz, 1H), 4.42–4.35 m, 1H, 4.16 (dd, *J* = 13.3, 7.0 Hz, 1H), 4.00–3.85 (m, 1H), 3.59 (d, *J* = 17.0 Hz, 1H), 2.26 (s, 3H), 1.15 (s, 9H). ^13^C NMR (101 MHz, DMSO-*d*_6_, presence of rotamers) δ 171.8; 167.6; 140.6; 139.6; 137.4; 136.7; 135.6; 129.7; 129.4; 129.2; 128.6; 128.4; 127.2; 127.0; 126.5; 122.4; 121.5; 119.1; 118.7; 116.0; 115.5; 111.9; 52.5; 50.9; 41.0; 29.5; 29.00; 21.1. HRMS (ESI): *m*/*z* calculated for C_30_H_33_N_3_O_2_ [(M + H)]^+^: 468.2646, found: 468.2649.

#### *N*-(2-(*tert*-Butylamino)-2-oxoethyl)-*N*-(2-(*p*-tolyl)-2-(1-tosyl-1*H*-indol-3yl)­ethyl)­benzamide (**12d**) (**LSPN1147**)

Synthesized from of **12a** (0.068 g), obtained as a white solid in 69% yield (0.064 g). mp 109.4 °C–111.6 °C. ^1^H NMR (400 MHz, DMSO-*d*_6_, presence of rotamers): δ 7.90–7.85 (m, 1H), 7.46 (d, *J* = 7.9 Hz, 2H), 7.43–7.31 (m, 8H), 7.29–7.27 (m, 3H), 7.17–7.04 (m, 1H), 6.99–6.94 (m, 3H), 4.74–4.66 (m, 1H), 4.11 (dd, *J* = 13.4, 6.5 Hz, 1H), 4.03–3.87 (m, 2H), 3.78 (s, 1H), 3.60 (d, *J* = 17.0 Hz, 1H), 2.26 (s, 6H), 1.17 (s, 9H). ^13^C NMR (100 MHz, DMSO-*d*_6_, presence of rotamers) δ 171.8; 167.6; 141.9; 140.4; 139.4; 137.4; 135.7; 130.6; 129.4; 129.3; 128.7; 128.4; 127.4; 127.0; 126.9; 126.5; 123.7; 121.7; 119.3; 118.8; 115.5; 114.8; 113.7; 110.0; 52.6; 51.0; 50.7; 29.5; 21.1. DEPT135 (100 MHz, DMSO-*d*_6_, presence of rotamers) δ 130.6 (CH); 129.6 (CH); 129.5; 129.4 (CH); 129.3 (CH); 128.7 (CH); 128.4 (CH); 128.3 (CH); 127.2 (CH); 127.0 (CH); 126.9 (CH); 126.6 (CH); 126.5 (CH); 121.7 (CH); 119.3 (CH); 119.0 (CH); 118.8 (CH); 110.0 (CH); 52.6 (CH_2_); 51.0 (CH_2_); 40.2 (CH); 29.0 (CH); 21.1 (CH). HRMS (ESI): *m*/*z* calculated for C_37_H_39_N_3_O_4_S [(M + H)]^+^: 622.2734, found: 622.2741.

#### Synthesis of 1*H*-Indole-2-carboxamide (**7**)^49^

In a 50 mL flask, a solution containing the carboxylic acid **5** (0.8 g, 5 mmol), DMF (six drops) and SOCl_2_ (0.4 mL, 7.5 mmol) in toluene (20 mL) was left under reflux for 2 h. Then, the solvent was removed under vacuum and the residue was redissolved in 1,4-dioxane (20 mL) and placed in an ice bath, under stirring. After 2 min, a NH_3_ solution (28–30% in water, 1.2 mL, 7.5 mmol) was added and the mixture was left stirring for 1 h at r.t. The solid formed was filtered and recrystallized from MeOH, to lead to amide **7** as a white solid in 97% yield. ^1^H NMR (400 MHz, DMSO-*d*_6_) δ 11.5 (s, 1H), 7.9 (s, 1H), 7.59 (d, *J* = 7.5 Hz, 1H), 7.40 (d, *J* = 7.5 Hz, 1H), 7.15 (t, *J* = 8.0 Hz, 1H), 7.11 (s, 1H), 6.97 (t, *J* = 8.0 Hz, 1H). ^13^C NMR (101 MHz, DMSO-*d*_6_) δ 163.3; 136.9; 132.2; 127.6; 123.7; 121.9; 120.1; 112.7; 103.5.

#### Synthesis of (1*H*-Indol-2-l)­methanamine (**8**)

To a suspension of LiAlH_4_ (0.24 g, 6.3 mmol) in anhydrous THF (20 mL) in a 50 mL flask, previously cooled in a water bath ice, amide **7** (0.78 g, 4.9 mmol) was added in portions over 10 min. Then, the mixture was heated at reflux for 7 h. The reaction crude was then cooled in an ice bath and a THF/water solution (6:4) was added to the flask, followed by stirring for 1 h at r.t., filtration and extraction with EtOAc (3 × 15 mL). The organic layer was dried with Na_2_SO_4_ and concentration in vacuum. The remaining solid was applied in the next step without further purification.

#### Synthesis of Nitroindole Derivatives (**10**)^25^

A mixture of indole (0.11g, 1 mmol) and β-nitrostyrene or *p*-methyl-β-nitrostyrene (1.1 mmol) was suspended in an open glass tube and heated to 100 °C. After the end of reaction (TLC monitoring), the reaction crude was purified by column chromatography (Hex/EtOAc 10–30%) leading to the C-3 alkylated products.

#### 3-(2-Nitro-1-phenylethyl)-1*H*-indole (**10a**)

The compund was obtained as a yellowish-brown oil in 82% yield. ^1^H NMR (400 MHz, CDCl_3_) δ 8.03 (s, 1H, *NH*), 7.40 (d, *J* = 7.9 Hz, 1H), 7.26 (s, 4H), 7.24–7.18 (m, 2H), 7.16–7.11 (m, 1H), 7.06–7.01 (m, 1H), 6.85 (d, *J* = 2.3 Hz, 1H), 5.12 (t, *J* = 8.0 Hz, 1H), 4.96 (dd, *J* = 12.5, 8.0 Hz, 1H), 4.84 (dd, *J* = 12.5, 8.0 Hz, 1H). ^13^C NMR (101 MHz, CDCl_3_) δ 139.4; 136.6; 128.9; 127.9; 127.6; 126.2; 122.7; 121.8; 119.9; 118.9; 114.2; 111.6; 79.6; 41.6.

#### 3-(2-Nitro-1-(*p*-tolyl)­ethyl)-1*H*-indole (**10b**)

The compund was obtained as a yellowish-brown oil in 76% yield. ^1^H NMR (400 MHz, CDCl_3_) δ 7.94 (s, 1H, *NH*), 7.35 (d, *J* = 7.9 Hz, 1H), 7.22 (dd, *J* = 8.1, 5.3 Hz, 1H), 7.11 (d, *J* = 8.1 Hz, 3H), 7.09–7.06 (m, 1H), 7.04–6.94 (m, 2H), 6.87 (d, *J* = 2.4 Hz, 1H), 5.05 (t, *J* = 8.0 Hz, 1H), 4.93 (dd, *J* = 12.4, 8.0 Hz, 1H), 4.80 (dd, *J* = 12.4, 8.0 Hz, 1H), 2.21 (s, 3H). ^13^C NMR (101 MHz, CDCl_3_) δ 137.3; 136.5; 136.2; 129.6; 127.7; 126.2; 122.7; 121.6; 119.9; 118.9; 114.6; 111.4; 79.7; 41.2; 21.0.

### Biology

#### In Vitro Cultivation of *Plasmodium falciparum*

The *P. falciparum* chloroquine sensitive 3D7 and multidrug-resistant strains Dd2, K1, Dd2^R^_DSM265, and 3D7^R^_MMV848 were maintained in continuous culture using the protocols described elsewhere.^50^

#### SYBR Green Assay to Evaluate the Inhibitory Activity of the Compounds against *P. falciparum*

The parasites were synchronized at the ring stage by treatment with 5% D-sorbitol (m/v) as described by Lambros and Vanderberg.^51^ After synchronization, the culture with 0.5% parasitemia and 2% hematocrit was added to 96-well plates and incubated with the compounds at concentrations ranging from 50 to 0.049 μM, obtained by 11 serial 2-fold dilutions. In each plate, negative and positive control wells were added in parallel, with nonparasitized red blood cells and parasitized red blood cells without compound addition, respectively and artesunate was used as the positive control. DMSO concentrations were kept below 0.1%. The plates were incubated for 72 h at 37 °C in a humidified incubator with an atmosphere of 5% CO_2_, 5% O_2_, and 90% N_2_. The detailed protocol is described elsewhere.^52^

#### Cytotoxicity in Human Hepatocellular Carcinoma (HepG2) Cells

The cytotoxic effects of the indole derivatives were evaluated against the human hepatocellular carcinoma cell line (HepG2). HepG2 cells were cultured in RPMI 1640 medium supplemented with 10% fetal bovine serum (v/v), 24 mmol L^–1^ sodium bicarbonate, 40 mg/mL gentamicin, and 10 mmol L^–1^ HEPES, pH 7.4. Upon reaching 80% confluence in the flask, the cells were treated with a 0.25% trypsin solution to break the attachment between the cells and the extracellular matrix. For cytotoxicity analysis, the cells were seeded at 30,000 cells per well (180 μL) in 96-well microplates and incubated for 24 h to allow cell adhesion. After this period, the cells were incubated with the compounds prepared in 7 serial dilutions with a factor of 2 (100 to 1.56 μM) for 72 h at 37 °C in a 5% CO_2_ atmosphere. Final DMSO concentrations were kept below 0.5% (v/v). Untreated cells were maintained as controls under the same conditions and pyronaridine was used as the positive control (tested in serial dilutions starting at 50 μM). The detailed protocol is described elsewhere.^53^

#### Calculation of the Selectivity Index (SI)

Having obtained the IC_50_ *^P.falc^* and CC_50_^HepG2^ values for the compounds, the selectivity index was determined using the following ratio

$$\mathrm{SI}:{{\mathrm{CC}}_{50}}^{\mathrm{HepG}2\mathrm{orHEK}293}/{{\mathrm{IC}}_{50}}^{P.\mathrm{falc}}$$

IS values above 10 are indicative of compounds well tolerated by the cellular model used.^33^

#### Antiplasmodial Activity against *P. falciparum* Resistant Strains

The antiplasmodial activity of the indole derivatives was determined against a representative panel of *P. falciparum* resistant strains. The panel included 3D7 (chloroquine-sensitive), Dd2 (resistant to chloroquine, mefloquine, and pyrimethamine), K1 (resistant to chloroquine, mefloquine, pyrimethamine, and sulfadoxine), Dd2^R^_DSM265 (resistant to DSM265, a *Pf*DHODH inhibitor), 3D7^R^_MMV848 (resistant to MMV848, a *Pf*PI4K inhibitor) and TM90C6B (resistant to atovaquone). The compounds of interest were incubated in 11 serial dilutions with a 2-fold factor, starting at 50 μM, with cultures at 0.5% parasitemia and 2% hematocrit. DMSO concentrations were kept below 0.1% (v/v), and DMSO was used as the vehicle control. The plates were incubated for 72 h, followed by the SYBR Green I assay to determine the inhibitory activity of the compounds for each resistant strain. As resistance-validation controls, pyrimethamine (3D7, Dd2, and K1), atovaquone (3D7 and TM90C6B), DSM265 (3D7 and Dd2^R^_DSM265), and MMV692848 (3D7 and 3D7^R^_MMV848) were used, and artesunate was used for all strains. The 50% inhibitory concentration (IC_50_) was determined by nonlinear regression analysis of the concentration–response curve using GraphPad Prism software (version 8.0.1). The resistance index was calculated as the ratio between the IC_50_ of the resistant strain and the IC_50_ of 3D7. RI values above 5 indicate cross-resistance.^34^

#### Speed of Action Assay

The protocol adapted from Le Manash et al.^26^ was used to determine whether the indole derivatives exhibited fast- or slow-acting inhibitory activity. Ninety-six–well plates were prepared with 3D7 *P. falciparum* cultures synchronized at the ring stage, and active derivatives were added in 11 serial dilutions starting at 50 μM. DMSO concentrations were maintained below 0.1% (v/v). Fast-acting controls included artesunate and chloroquine, while slow-acting controls included pyrimethamine and atovaquone. Each plate was exposed to the compounds for different time intervals (24, 48, or 72 h). After 24 h, the first plate was washed with culture medium to remove the compounds and incubated for an additional 48 h. The second plate was washed after 48 h of drug exposure and then incubated for an additional 24 h. Following a total incubation period of 72 h at 37 °C, parasites from each condition (24, 48, and 72 h of compound exposure) were diluted 1:25 with fresh red blood cells in complete medium and cultured for an additional 4 days to assess parasite recovery after drug removal. IC_50_ values for each incubation period were determined at the end of both the 72 h assay (speed-of-action assay) and the 168 h assay (four-day recovery assay) using the SYBR Green I method. IC_50_ values were calculated by nonlinear regression analysis of concentration–response curves using GraphPad Prism 8 (GraphPad Software, San Diego, CA, USA). Statistical significance was evaluated using ANOVA. In addition to quantitative assessment of speed of action, parasite morphological development was examined. Parasites were exposed to **LSPN954** and **LSPN959** at 10× IC_50_ for 24 h, after which the compounds were washed out. Morphological progression was monitored at 24, 48, and 72 h using thin blood smear analysis (documented by optical microscopy imaging). Fast- and slow-acting controls were included for comparison.

#### Combination Assay with Artesunate

The combination assay was performed with an adaptation of the work by Fivelman et al.^54^ The compounds of interest and artesunate were diluted and combined in a 96-well plate in seven fixed ratio combinations (1:0, 6:1, 5:2, 4:3, 3:4, 2:5, 1:6, 0:1). The initial concentrations of all compounds were set as 10× IC_50_ and serial dilutions of these combinations were prepared and incubated with culture at 0.5% parasitemia and 2% hematocrit to assess their antiplasmodial activity against *P. falciparum*. DMSO concentrations were kept below 0.1% (v/v). After 72 h of incubation, the treatment with SYBR Green I was performed to determine the IC_50_ value for each combination. The additivity isobole was calculated based on the Hand model.^55^ The fractional inhibitory concentration (FIC_50_) values were expressed as IC_50_ equivalents and determined for the eight different proportions of the compounds with artesunate. The detailed protocol is described elsewhere.^53^

## Supplementary Material



## Data Availability

The data underlying this study are available in the published article and its online Supporting Information.

## Notes

Methods for cytotoxicity in human HEK293 cells, chemical rescue assay (IPP), and purification of peptidomimetics; physicochemical properties of peptidomimetics; ^1^H and ^13^C NMR spectra for target compounds **4j**–**4m**, **6a**–**6g**, **7**, **9a**–**9e**, **10a**, and **12a**–**12d** and DEPT135 NMR spectra for target compounds **4j**–**4m**, **9b**, **9e**, and **12a**–**12d**; UPCC analysis of peptidomimetics; and stability test of compounds **LSPN954** and **LSPN1157** (PDF)
