# Supplementary material for: Indole Peptidomimetics Show Potent and Selective Activity against Drug-Resistant Plasmodium falciparum
Source: ACS Omega. 2026 Mar 21;11(13):20722–37. doi: 10.1021/acsomega.5c12662 (PMC13063074; doi:10.1021/acsomega.5c12662)
Supplement: Supplementary file 1 [file ao5c12662_si_001.pdf]

Supporting Information
For

Indole Peptidomimetics Show Potent and Selective Activity Against Drug-Resistant *Plasmodium falciparum*

Marcelo Augusto Pereira Januário [a§], Talita Alvarenga Valdes [b§],
Sarah El Chamy Maluf [b], Giovana Rossi Mendes [b], Vinicius Bonatto [b], Igor M. R. Moura [b],
Penina S. Mourão [a], Arlene G. Corrêa [a*], Rafael Victorio Carvalho Guido [b*]

[a] *Centre of Excellence for Research in Sustainable Chemistry, Department of Chemistry, Federal University of São Carlos, São Carlos, 13565-905, Brazil*
[b] *São Carlos Institute of Physics, University of São Paulo, São Carlos, 13563-120, Brazil*

[§] *M.A.P.J and T.A.V. contributed equally to this work*

* Email: agcorrea@ufscar.br
* Email: rvcguido@usp.br

List of Supplementary Figures

Figure S1. Molecular Weight (MW) in Da of each compound. 8
Figure S2. Number of Hydrogen Bond Donors (HBD) for each compound. 8
Figure S3. Number of Hydrogen Bond Acceptors (HBA) for each compound. 8
Figure S4. Topological Polar Surface Area (TPSA) in Å² values for the compounds. 9
Figure S5. Distribution of logP values for the analyzed compounds. 9
Figure S6. Distribution of logS values for the analyzed compounds. 9
Figure S7. ^{1}H NMR of **4j** (MeOD-d_4,400 MHz, mixture of rotamers). 10
Figure S8. ^{13}C NMR of **4j** (MeOD-d_4,400 MHz, mixture of rotamers). 10
Figure S9. DEPT135 NMR of **4j** (MeOD-d_4,400 MHz, mixture of rotamers). 11
Figure S10. ^{1}H NMR of **4k** (MeOD-d_4,400 MHz, mixture of rotamers) with water signal suppression. 11
Figure S11. DEPT135 NMR of **4k** (MeOD-d_4.100 MHz, mixture of rotamers)............ 12
Figure S12. ^{13}C NMR of **4k** (MeOD-d_4.100 MHz, mixture of rotamers). 12
Figure S13· ^{1}H NMR of **4m** (MeOD-d_4, 400 MHz, mixture of rotamers)........................ 13
Figure S14. ^{13}C NMR of **4m** (MeOD-d_4, 400 MHz, mixture of rotamers). 13
Figure S15. DEPT135 NMR of **4m** (MeOD-d_4, 400 MHz, mixture of rotamers). 14
Figure S16. ^{1}H NMR of **4l** (MeOD-d_4,400 MHz, mixture of rotamers). 14
Figure S17. ^{13}C NMR of **4l** (MeOD-d_4, 100 MHz, mixture of rotamers). 15
Figure S18. DEPT135 NMR of **4l** (MeOD-d_4.100 MHz, mixture of rotamers). 15
Figure S19. ^{1}H NMR of **6a** ($CDCl_3$, 400 MHz). 16
Figure S20. ^{13}C NMR of **6a** ($CDCl_3$, 100 MHz). 16
Figure S21. [13]H NMR of **6b** ($CDCl_3$, 400 MHz, *presence of rotamers*). 17
Figure S22. ^{13}C NMR of **6b** ($CDCl_3$,100 MHz, *presence of rotamers*). 17
Figure S23. [13]H NMR of **6c** ($CDCl_3$, 400 MHz, mixture of rotamers). 18
Figure S24. ^{13}C NMR of **6c** ($CDCl_3$, 100 MHz, mixture of rotamers). 18
Figure S25. ^{1}H NMR of **6d** (DMSO-d_6, 400 MHz, *presence of rotamers*). 19
Figure S26. ^{13}C NMR of **6d** (DMSO-d_6,100 MHz, *presence of rotamers*). 19
Figure S27. ^{1}H NMR of **6e** ($CDCl_3$, 400 MHz, mixture of rotamers). 20
Figure S28. ^{13}C NMR of **6e** ($CDCl_3$, 100 MHz, mixture of rotamers). 20
Figure S29. ^{13}C NMR of **6e** ($CDCl_3$, 100 MHz, *presence of rotamers*). 21
Figure S30. ^{1}H NMR of **6f** (DMSO-d_6, 400 MHz, *presence of rotamers*). 21
Figure S31. ^{13}C NMR of **6f** (DMSO-d_6, 100 MHz, *presence of rotamers*). 22
Figure S32. ^{1}H NMR of **6g** ($CDCl_3$, at 400 MHz, *presence of rotamers*). 22
Figure S33. ^{1}H NMR of **6g** in $CDCl_3$ (400 MHz, *presence of rotamers*). 23
Figure S34. ^{1}H NMR of **9a** (DMSO-d_6, 400 MHz, *presence of rotamers*). 23
Figure S35. ^{13}C NMR of **9a** (DMSO-d_6, 100 MHz, mixture of rotamers). 24
Figure S36. ^{1}H NMR of **9d** in DMSO-d_6, 400 MHz, mixture of rotamers). 24
Figure S37. ^{13}C NMR of **9d** (DMSO-d_6, 100 MHz, mixture of rotamers). 25
Figure S38. ^{1}H NMR of **9b** (DMSO-d_6, 400 MHz, mixture of rotamers). 25
Figure S39. ^{1}H NMR of **9b** (DMSO-d_6, 100 MHz, mixture of rotamers). 26
Figure S40. DEPT135 NMR of **9b** (DMSO-d_6, 100 MHz, mixture of rotamers). 26
Figure S41. ^{1}H NMR of **9c** (DMSO-d_6, 400 MHz, *presence of rotamers*). 27
Figure S42. ^{13}C NMR of **9c** (DMSO-d_6, 100 MHz, mixture of rotamers). 27

Figure S43. ^{1}H NMR of **9e** (DMSO-d_6, 400 MHz, mixture of amide and Boc rotamers) with water signal suppression. 28
Figure S44. ^{1}H NMR of **9e** (DMSO-d_6, 400 MHz, mixture of amide and Boc rotamers).28
Figure S45. ^{13}C NMR of **9e** (DMSO-d_6, 400 MHz, mixture of amide and Boc rotamers). 29
Figure S46. DEPT135 NMR of **9e** in DMSO-d_6 (400 MHz, mixture of amide and Boc rotamers). 29
Figure S47. ^{1}H NMR of **10a** ($CDCl_3$, 400 MHz). 30
Figure S48. ^{13}C NMR of **10a** ($CDCl_3$, 100 MHz). 30
Figure S49. ^{1}H NMR of **12a** (DMSO-d_6, 400 MHz, presence of rotamers). 31
Figure S50. ^{13}C NMR of **12a** (DMSO-d_6, 400 MHz, presence of rotamers). 31
Figure S51. DEPT135 NMR of **12a** (DMSO-d_6, 400 MHz, mixture of rotamers). 32
Figure S52. ^{1}H NMR of **12b** (DMSO-d_6, 400 MHz, mixture of rotamers). 32
Figure S53. ^{13}C NMR of **12b** (DMSO-d_6, 400 MHz, mixture of rotamers). 33
Figure S54. ^{1}H NMR **12c** (DMSO-d_6, 400 MHz, mixture of rotamers). 33
Figure S55. ^{13}C NMR of **12c** (DMSO-d_6, 400 MHz, mixture of rotamers). 34
Figure S56. DEPT135 NMR of **12c** (DMSO-d_6, 400 MHz, mixture of rotamers). 34
Figure S57. ^{1}H NMR of **12d** (DMSO-d_6, 400 MHz, mixture of rotamers). 35
Figure S58. ^{13}C NMR of **12d** (DMSO-d_6,100 MHz, mixture of rotamers). 35
Figure S59. DEPT135 NMR of **12d** (DMSO-d_6,100 MHz, mixture of rotamers). 36
Figure S60. ^{1}H NMR **7** (DMSO-d_6, 400 MHz). 36
Figure S61. ^{13}C NMR of **7** (DMSO-d_6, 400 MHz). 37
Figure S62. Chromatogram of compound **LSPN929.** 38
Figure S63. Chromatogram of compound **LSPN932.** 38
Figure S64. Chromatogram of compound **LSPN952.** 39
Figure S65. Chromatogram of compound **LSPN954.** 39
Figure S66. Chromatogram of compound **LSPN956.** 40
Figure S67. Chromatogram of compound **LSPN958.** 40
Figure S68. Chromatogram of compound **LSPN959.** 41
Figure S69. Chromatogram of compound **LSPN1139.** 41
Figure S70. Chromatogram of compound **LSPN1141.** 42
Figure S71. Chromatogram of compound **LSPN1148.** 42
Figure S72. Chromatogram of compound **LSPN1149.** 43
Figure S73. Chromatogram of compound **LSPN1150.** 43
Figure S74. Chromatogram of compound **LSPN1151.** 44
Figure S75. Chromatogram of compound **LSPN1153.** 44
Figure S76. Chromatogram of compound **LSPN1154.** 45
Figure S77. Chromatogram of compound **LSPN1155.** 45
Figure S78. Chromatogram of compound **LSPN1157.** 46
Figure S79. Stability test of compound **LSPN954**: A) MeOH, B) 72 h, C) 48 h, D) 24 h, E) 0 h, and F) compound **LSPN954** standard**.** 47
Figure S80. Stability test of compound **LSPN1157**: A) MeOH, B) 72 h, C) 48 h, D) 24 h, E) 0 h, and F) compound **LSPN1157** standard. 48

METHODS

Cytotoxicity in human HEK293 cells

The cytotoxic effects of the indole derivatives were evaluated in the human HEK293 cell line (derived from human embryonic kidney). HEK293 cells were cultured in RPMI 1640 medium supplemented with 10% fetal bovine serum (v/v), 24 mmol·L^{-1} sodium bicarbonate, 40 mg/mL gentamicin, and 10 mmol·L^{-1} HEPES (pH 7.4). For cytotoxicity analysis, cells were seeded at 20.000 cells per well (180 μL) in 96-well microplates and incubated for 24 h to allow cell adhesion. After this period, compounds were prepared in 7 serial two-fold dilutions (100 to 1.56 μM) and added to the cells, which were then incubated for 72 h at 37 °C in a 5% CO_2 atmosphere. Final DMSO concentrations were kept below 0.5% (v/v). Untreated cells were maintained as controls under the same conditions, and pyronaridine was used as the positive control (tested in serial dilutions starting at 50 μM). At the end of the incubation, concentrations at which compounds were insoluble and precipitated were assessed by microscopy and excluded from the analysis. Cell viability was determined by adding 40 μL of resazurin (0.15 mg/mL) to each well, followed by incubation for 4 h at 37 °C. Fluorescence was measured using a Varioskan LUX multimode microplate reader (Thermo Fisher Scientific, Waltham, MA, USA) (λex = 560 nm; λem = 590 nm). The 50% cytotoxic concentration (CC_{50}^{HEK293}) was determined by non-linear regression analysis of the concentration–response curve using GraphPad Prism version 8.0.1 (GraphPad Software, San Diego, CA, USA).

Chemical rescue assay (IPP)

The chemical rescue assay was performed as described by Yeh and DeRisi[1] to compare the antiplasmodial activity of the compounds in the presence and absence of isopentenyl pyrophosphate (IPP), purchased from Isoprenoids, LC (Florida, USA). Briefly, *P. falciparum* 3D7 parasites synchronized at the ring stage were cultured in 96-well plates at 0.5% parasitemia and 2% hematocrit. Non-parasitized red blood cells and parasitized red blood cells without compound addition were used as negative and positive growth controls, respectively. **LSPN954** and **LSPN959** were tested in technical duplicate in 11 serial two-fold dilutions starting at 50 μM, and evaluated with and without IPP supplementation at a final concentration of 200 μM. Fosmidomycin (FOS) was used as the positive control for chemical rescue and was tested in serial dilutions starting at 20 μM. Artesunate was used as the negative control for chemical rescue and was tested in serial dilutions starting at 0.3 μM. Plates were incubated for 72 h, and parasite growth was quantified using the SYBR Green I assay. The assay was performed in two independent experiments. IC_{50} values were obtained by non-linear regression of concentration–response curves (GraphPad Prism).

Purification of peptidomimetics

All synthetic compounds were purified by flash column chromatography and characterized by NMR and HRMS analyses. The purity of the sixteen compounds currently available in our stock (Table S2) was determined using a Waters ACQUITY UltraPerformance Convergence Chromatography (UPCC) system, in a Viridis BEH Column (130 Å, 1.7 µm 3.0 mm x 100 mm, 1/pkg achiral column), CO_2:MeOH (90:10 and 95:5) as mobile phase, and wavelength range of 190–400 nm.

Stability test of peptidomimetics

The tested compounds (2 mg) were initially dissolved in DMSO (2 mL). An aliquot (56.5 µL for **LSPN954** and 45.5 µL for **LSPN1157**) was then transferred to a vial and diluted with RPMI 1640 culture medium to a final volume of 2 mL, affording a final concentration of 50 µM. Stability studies were conducted at 0, 24, 48, and 72 h. At the end of each time point, the compound was extracted with ethyl acetate (3 × 2 mL). The combined organic layers were dried over anhydrous Na_2SO_4 and concentrated under reduced pressure. The samples were subsequently analysed by UPCC, confirming that the compounds remained stable throughout the experimental period.

Table S1. Physicochemical properties of the indole derivatives, including predicted aqueous solubility (logS), partition coefficient (logP), molecular weight (MW), number of hydrogen bond acceptors (HBA), number of hydrogen bond donors (HBD), and topological polar surface area (TPSA, Å^2). MW, HBA, HBD, TPSA and LogP were all calculated using RDKit. LogS was calculated using SolTranNet (GitHub - gnina/SolTranNet: Implementation of the SolTranNet tool utilizing the molecular transformer to predict aqueous solubility).

LSPN	logS	LogP	HBA	HBD	TPSA (Å^2)	MW (Da)
**953 (4g)**	-6.31	6.20	4	1	74.65	564.73
**954 (4i)**	-6.31	6.20	4	1	74.65	564.73
**955 (4j)**	-6.00	5.90	5	1	83.88	580.73
**956 (4f)**	-6.31	6.50	4	1	74.65	676.60
**957 (4c)**	-5.90	5.90	4	1	74.65	550.70
**958 (4b)**	-6.56	6.43	4	1	74.65	576.74
**959 (4k)**	-6.16	6.66	4	1	74.65	629.60
**1139 (4m)**	-6.29	6.20	4	1	74.65	564.73
**1141 (6b)**	-6.86	5.47	2	2	65.20	425.53
**1142 (6c)**	-7.09	6.08	2	2	65.20	551.43
**1143 (6a)**	-5.26	3.73	2	2	65.20	349.43
**1146 (6e)**	-6.55	5.25	3	1	54.34	439.56
**1147 (12d)**	-7.08	6.68	5	1	88.48	621.80
**1148 (4l)**	-6.22	6.21	5	1	83.88	594.76
**1149 (6g)**	-6.89	5.40	2	2	65.20	439.56
**1150 (6f)**	-6.39	4.75	5	1	88.48	503.62
**1151 (6d)**	-5.05	3.73	2	2	65.20	349.43
**1153 (9b)**	-5.84	5.25	3	1	54.34	453.59
**1154 (9c)**	-6.24	4.74	5	1	88.48	517.65
**1155 (9e)**	-5.29	4.98	5	1	80.64	463.58
**1156 (9d)**	-4.61	3.73	2	2	65.20	363.46
**1157 (12a)**	-5.89	5.36	2	2	65.20	453.59
**1158 (12c)**	-6.17	5.67	2	2	65.20	467.61
**1159 (12b)**	-6.92	6.38	5	1	88.48	607.78

Table S2. Purity of the peptidomimetics.

Compound	Purity (%)
**LSPN929 (4h)**	>99
**LSPN932 (3a)**	>99
**LSPN952 (4a)**	>99
**LSPN954 (4i)**	>99
**LSPN956 (4f)**	93
**LSPN958 (4b)**	95
**LSPN959 (4k)**	94
**LSPN1139 (4m)**	93
**LSPN1141 (6b)**	95
**LSPN1149 (6g)**	94
**LSPN1150 (6f)**	>99
**LSPN1151 (6d)**	>99
**LSPN1153 (9b)**	95
**LSPN1154 (9c)**	95
**LSPN1155 (9e)**	>99
**LSPN1157 (12a)**	97

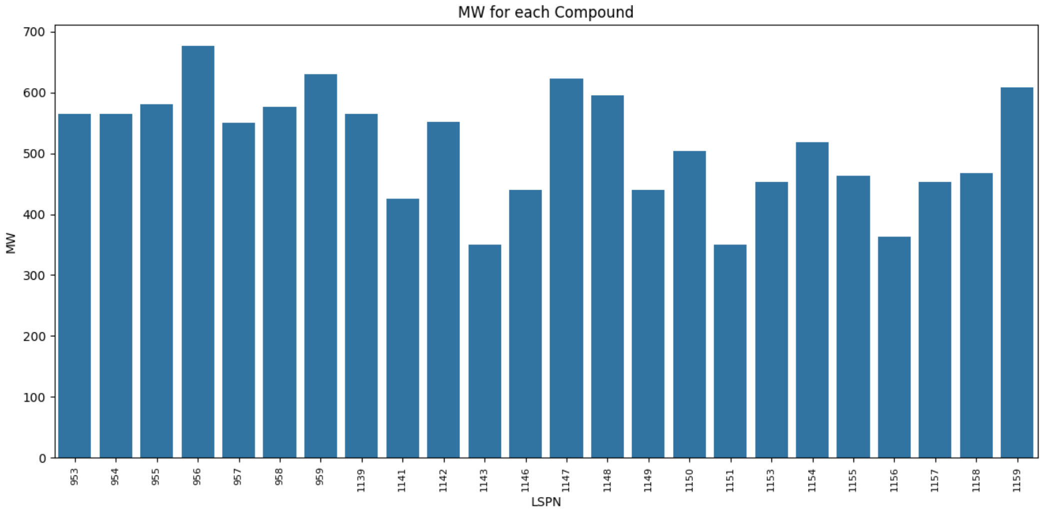

Figure S1. Molecular Weight (MW) in Da of each compound.

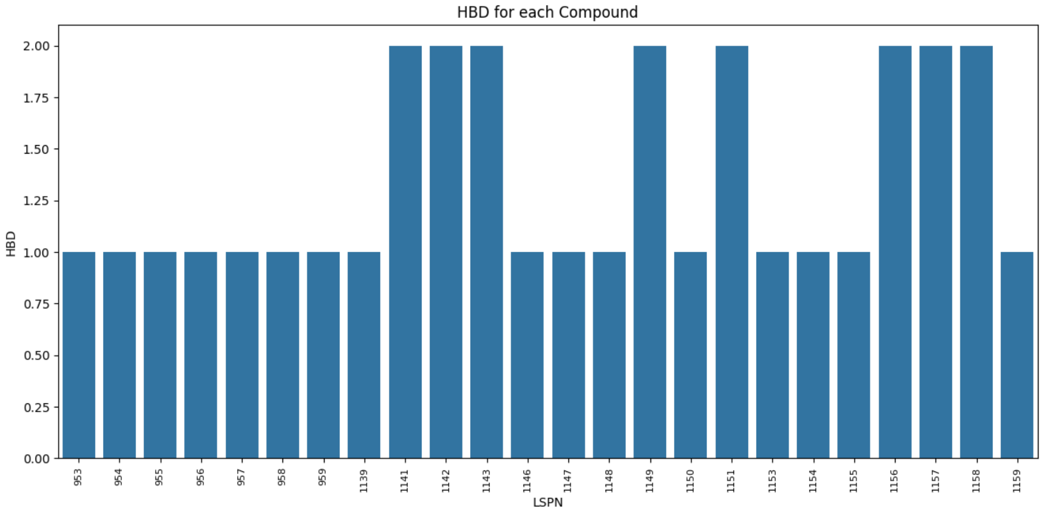

Figure S2. Number of Hydrogen Bond Donors (HBD) for each compound.

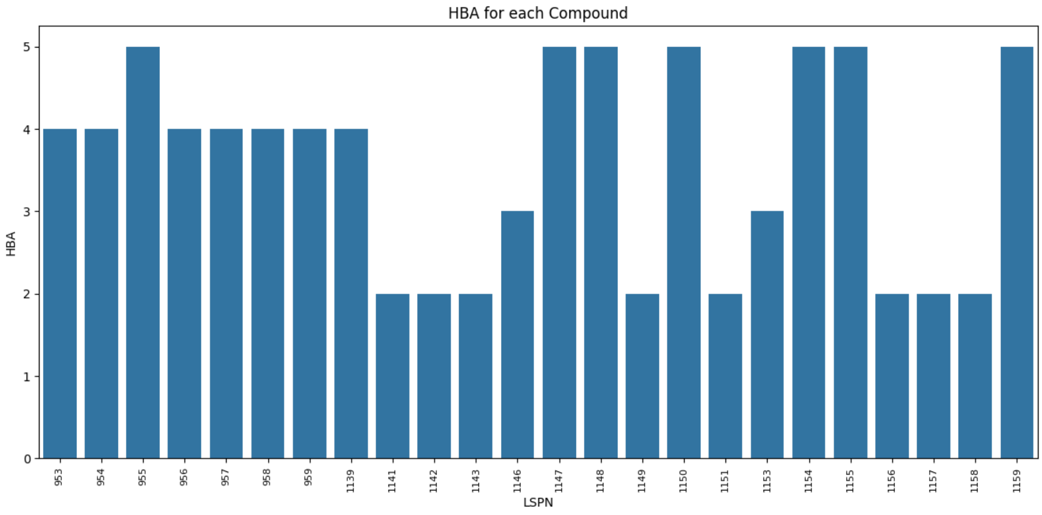

Figure S3. Number of Hydrogen Bond Acceptors (HBA) for each compound.

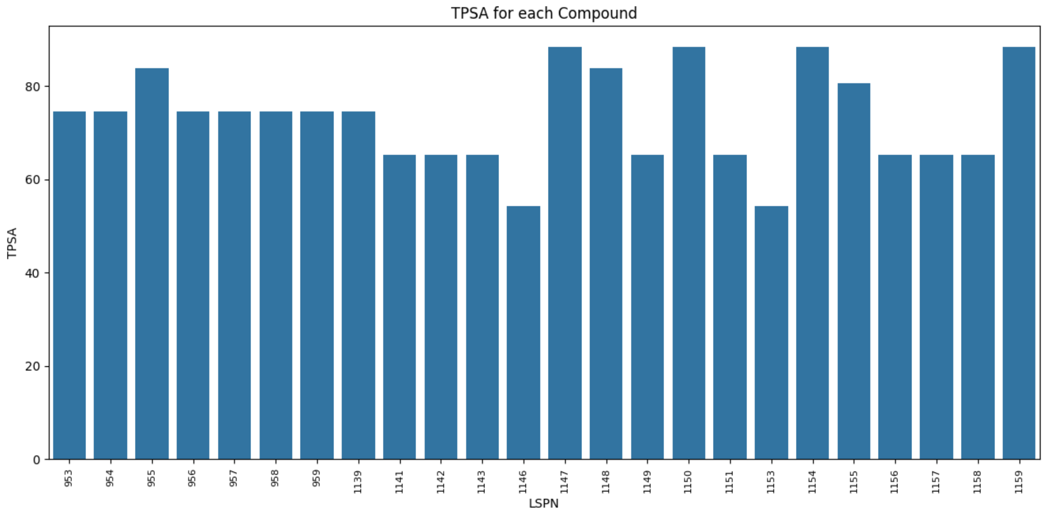

Figure S4. Topological Polar Surface Area (TPSA) in Å² values for the compounds.

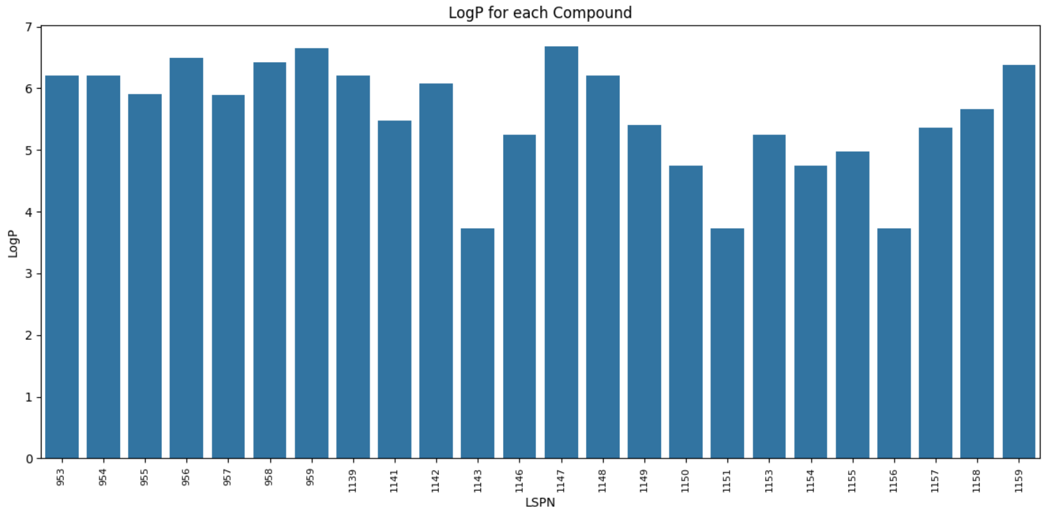

Figure S5. Distribution of logP values for the analyzed compounds.

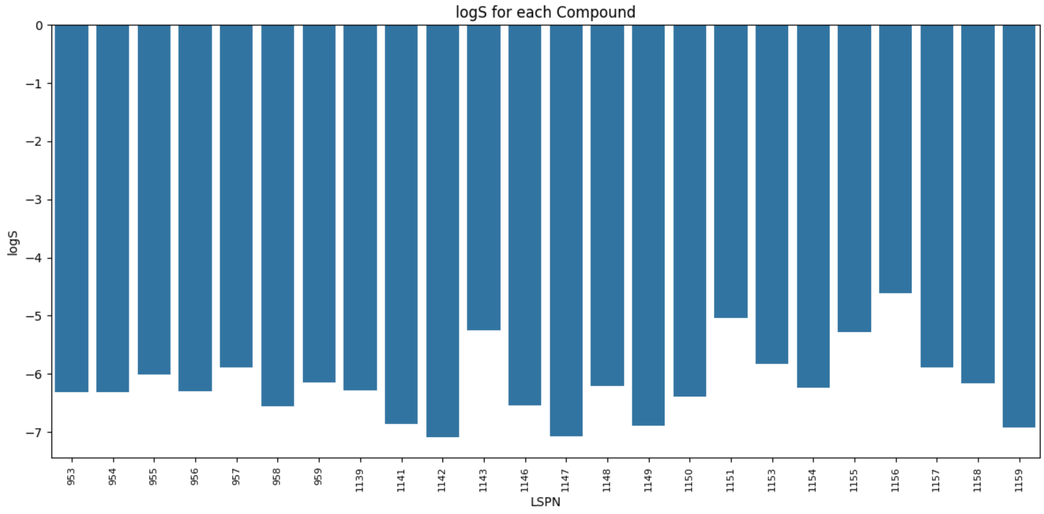

Figure S6. Distribution of logS values for the analyzed compounds.

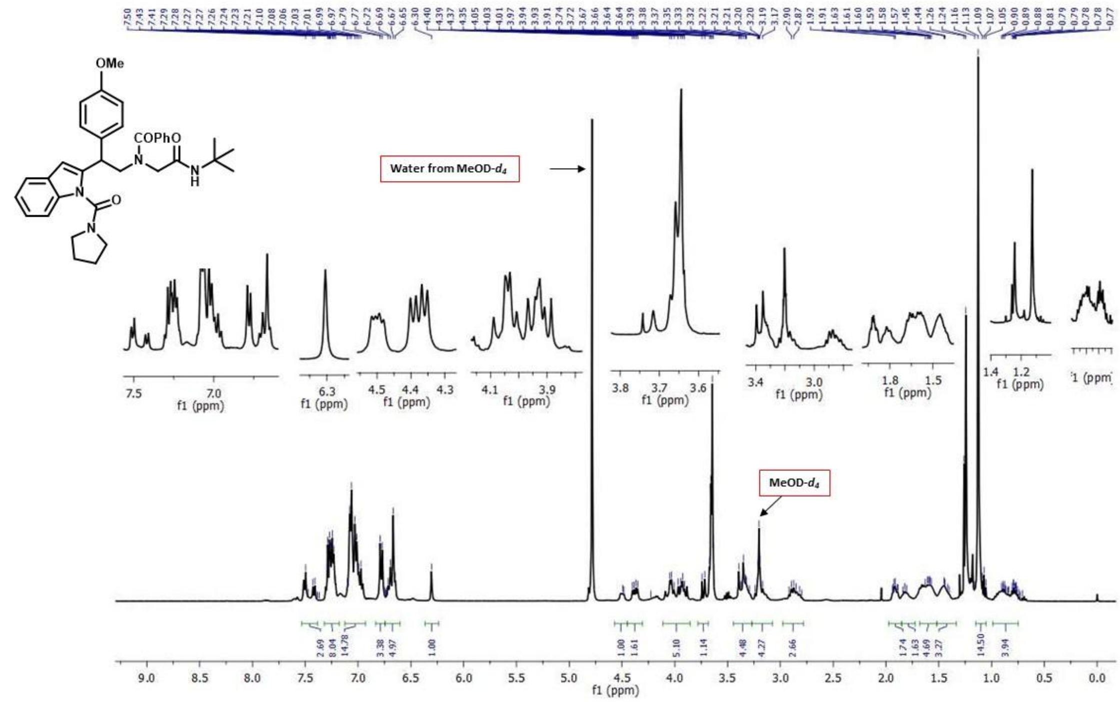

Figure S7. ^{1}H NMR of **4j** (MeOD-d_4,400 MHz, mixture of rotamers).

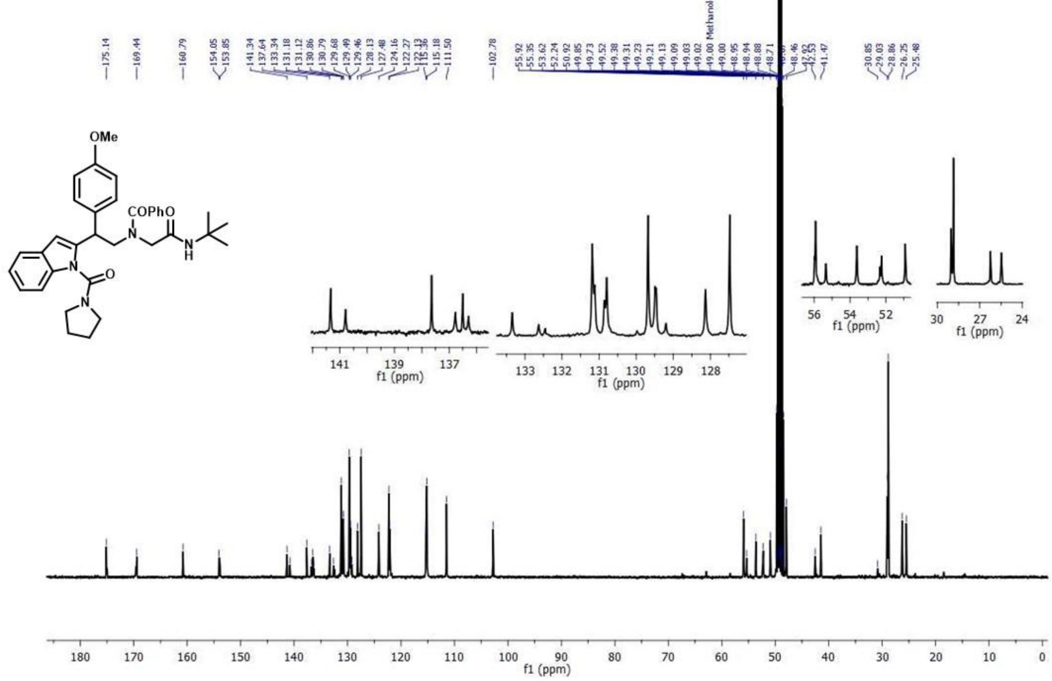

Figure S8. ^{13}C NMR of **4j** (MeOD-d_4,400 MHz, mixture of rotamers).

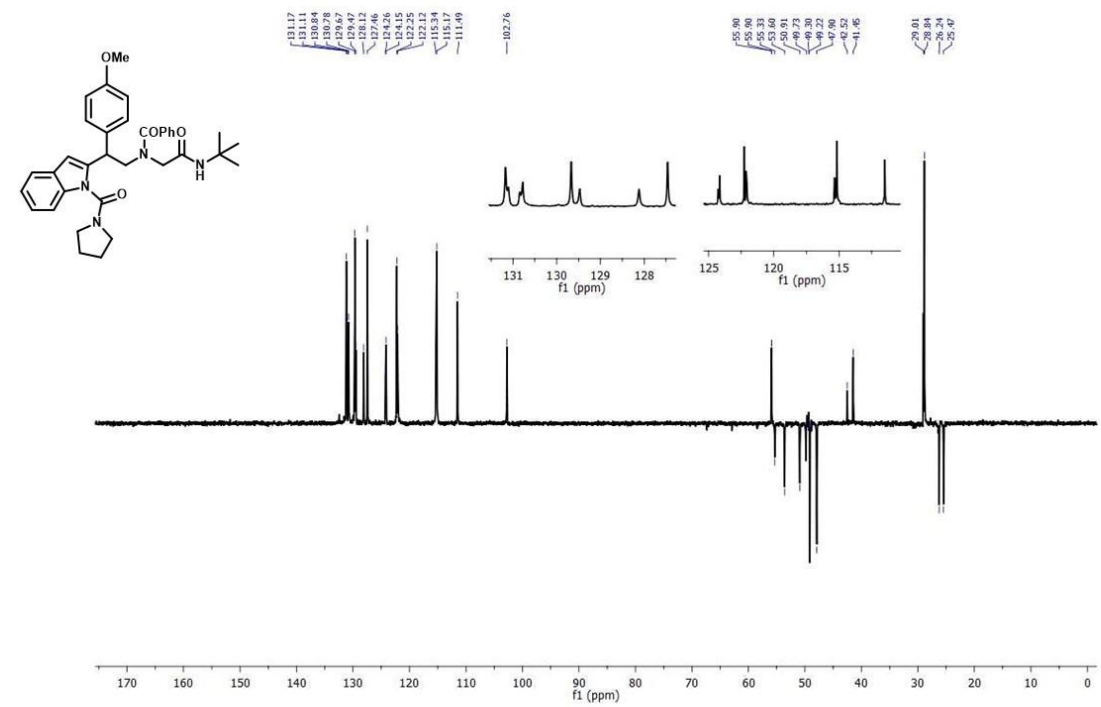

Figure S9. DEPT135 NMR of **4j** (MeOD-d_4,400 MHz, mixture of rotamers).

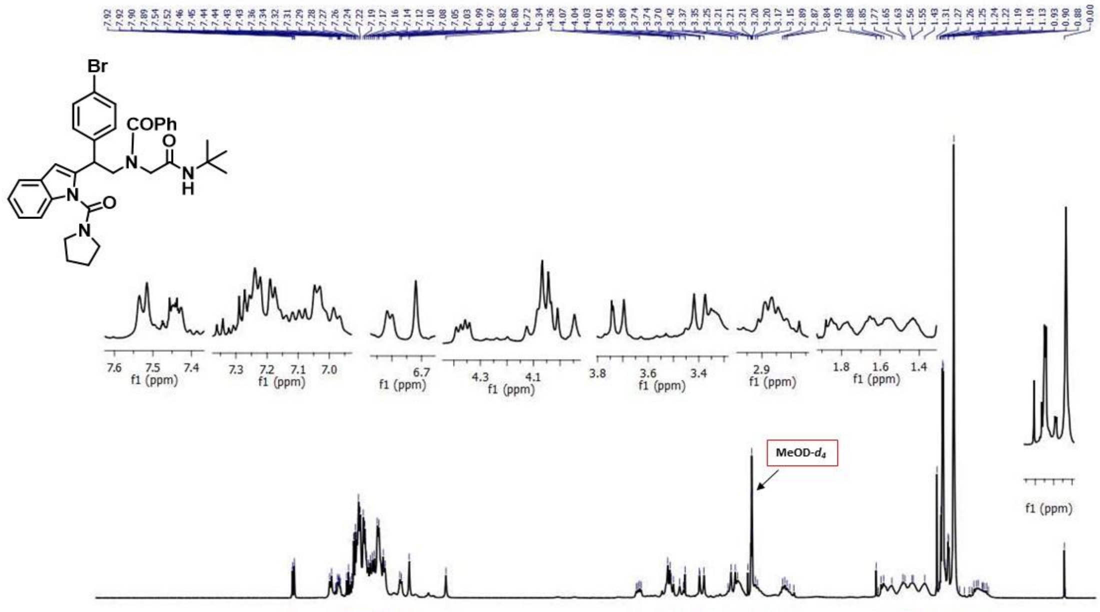

Figure S10. ^{1}H NMR of **4k** (MeOD-d_4,400 MHz, mixture of rotamers) with water signal suppression.

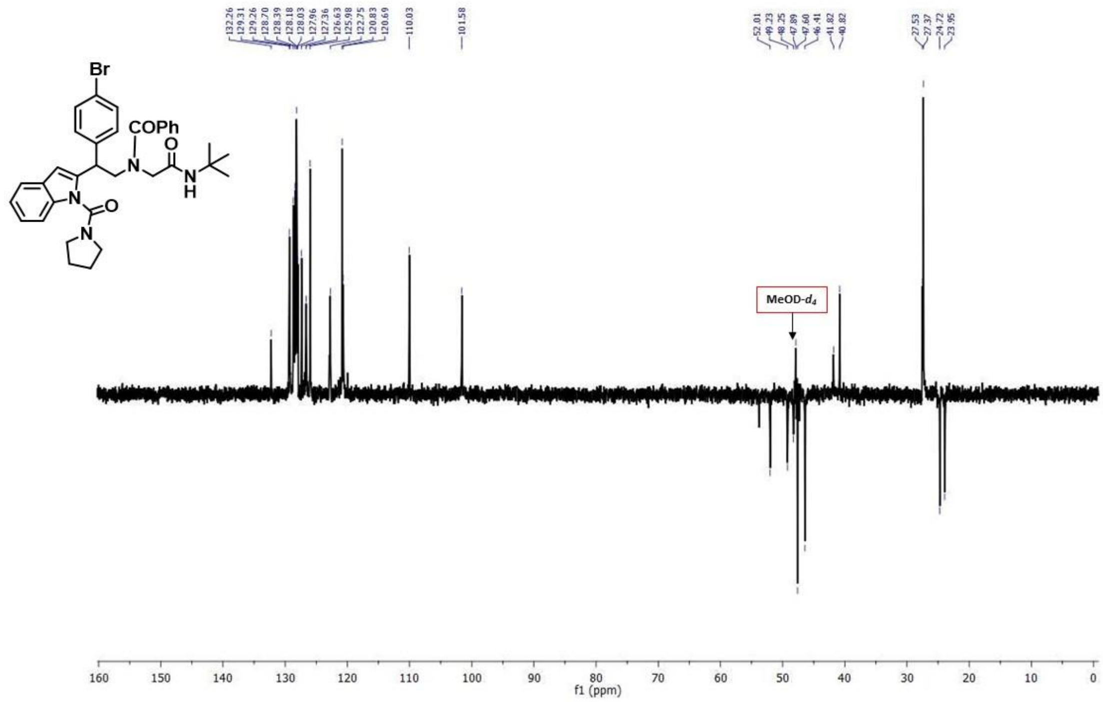

Figure S11. DEPT135 NMR of **4k** (MeOD-d_4.100 MHz, mixture of rotamers).

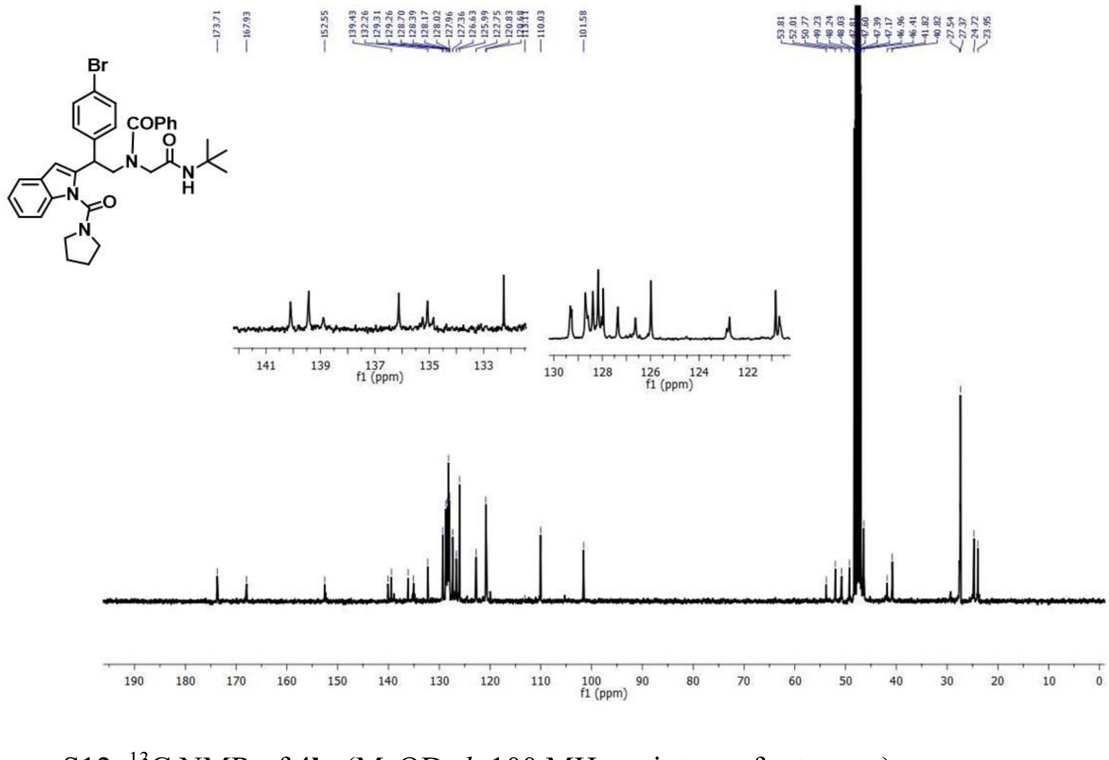

Figure S12. ^{13}C NMR of **4k** (MeOD-d_4.100 MHz, mixture of rotamers).

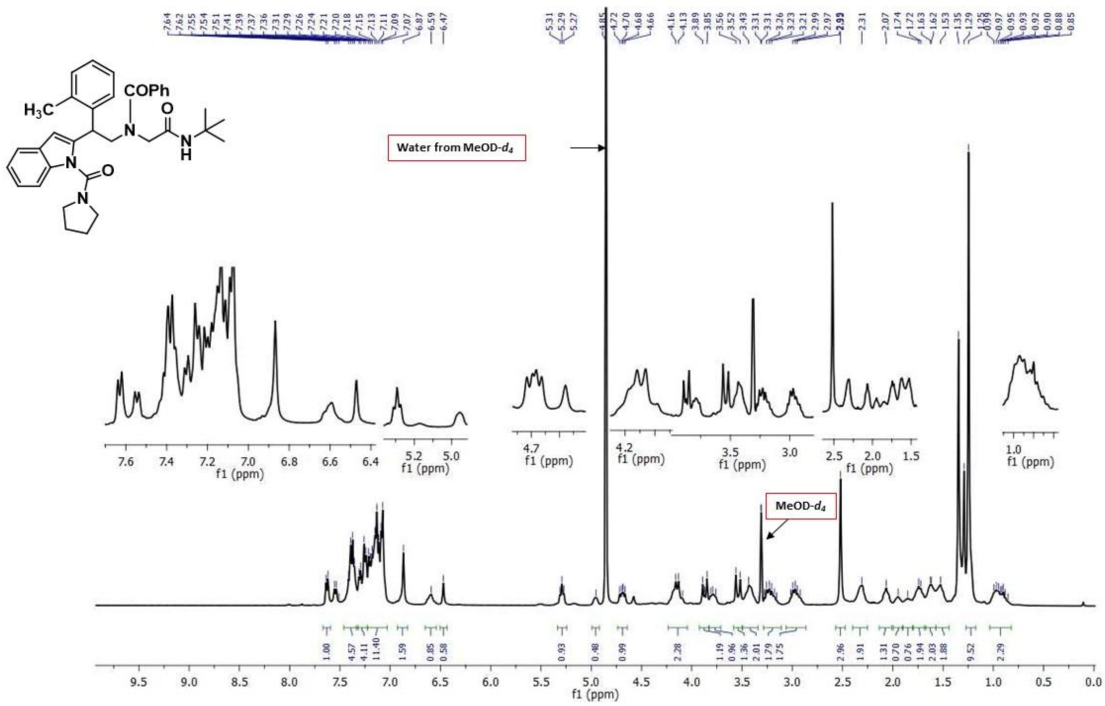

Figure S13. ^{1}H NMR of **4m** (MeOD-d_4, 400 MHz, mixture of rotamers).

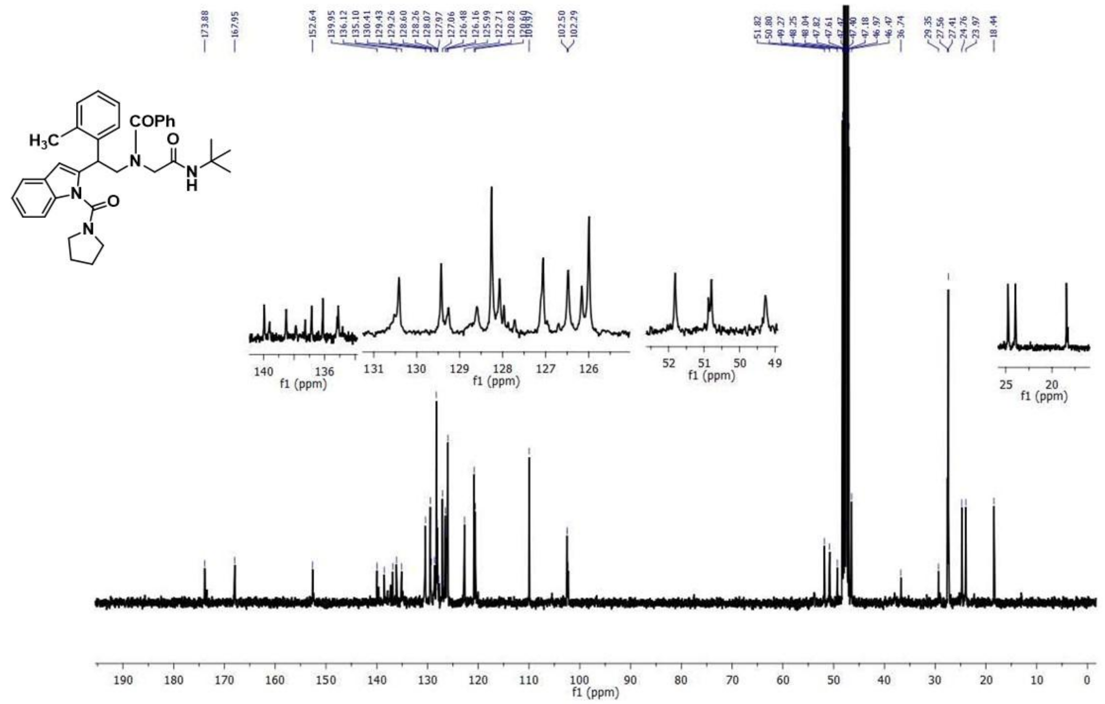

Figure S14. ^{13}C NMR of **4m** (MeOD-d_4, 400 MHz, mixture of rotamers).

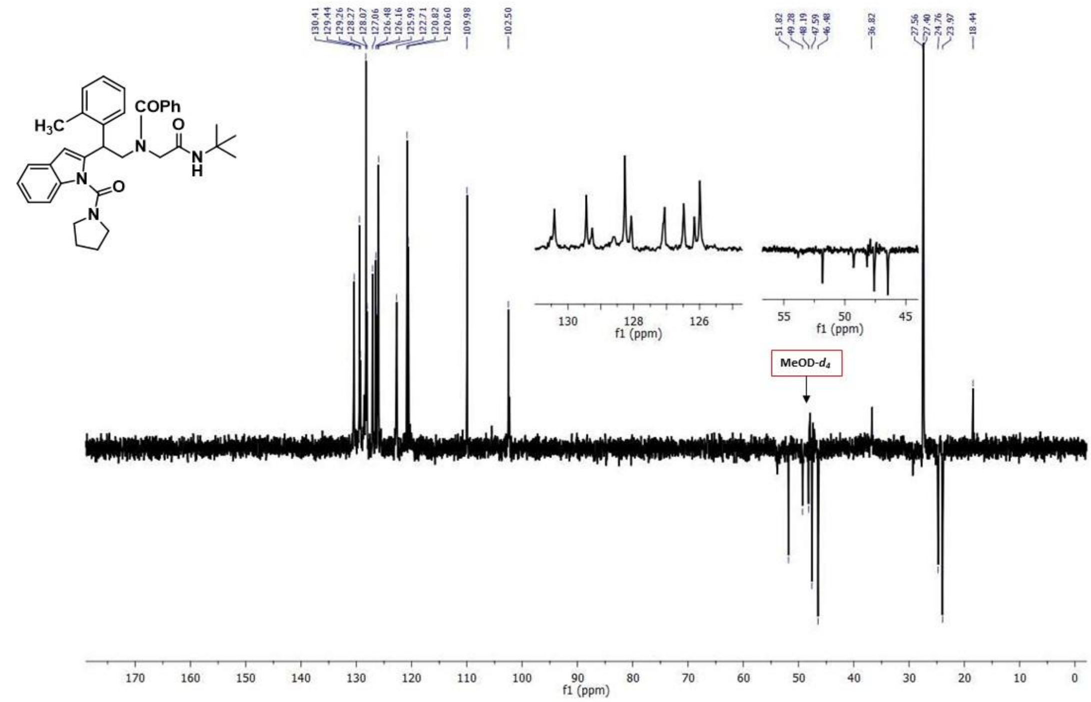

Figure S15. DEPT135 NMR of **4m** (MeOD-d_4, 400 MHz, mixture of rotamers).

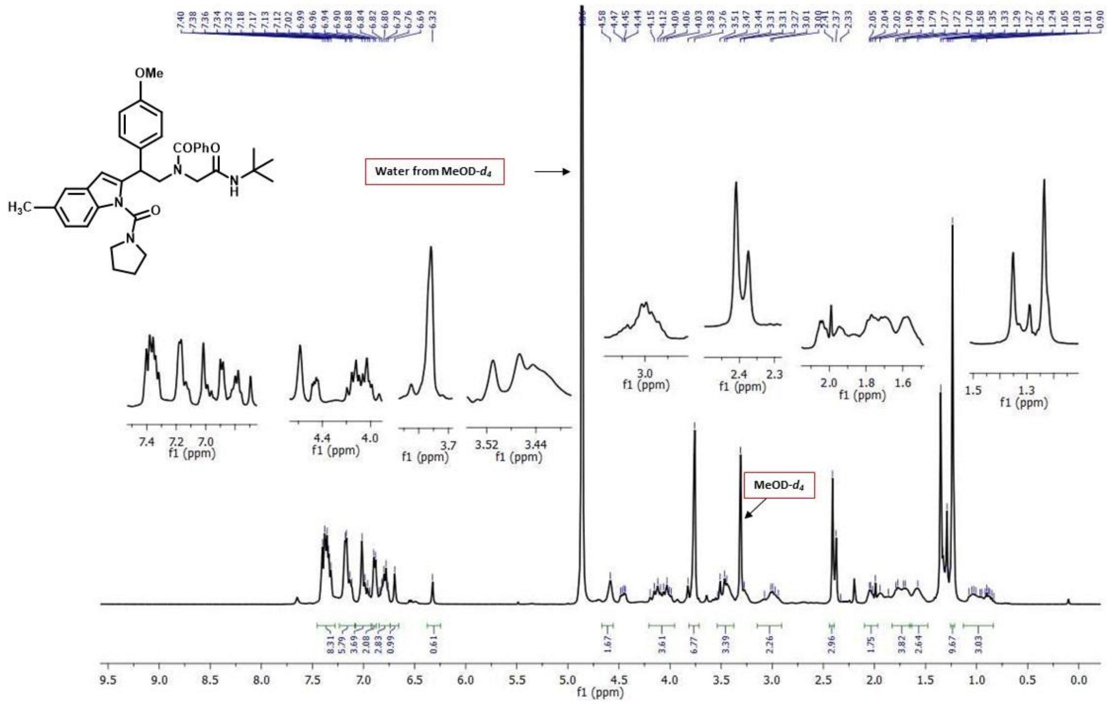

Figure S16. ^{1}H NMR of **4l** (MeOD-d_4,400 MHz, mixture of rotamers).

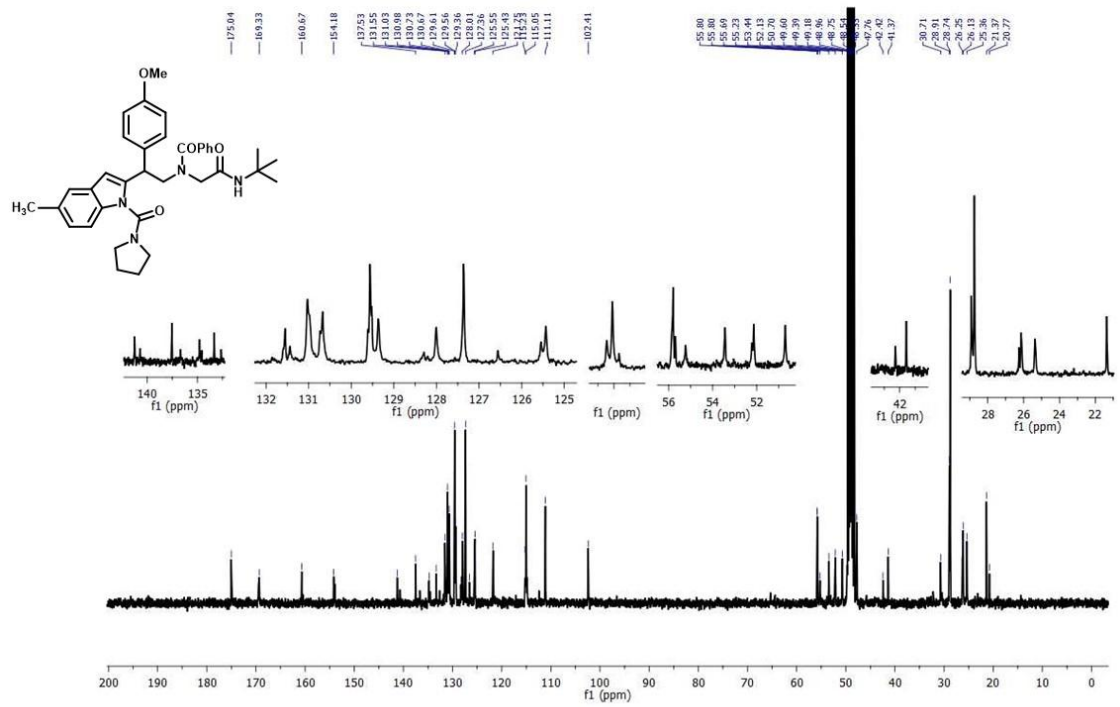

Figure S17. ^{13}C NMR of **4l** (MeOD-d_4, 100 MHz, mixture of rotamers).

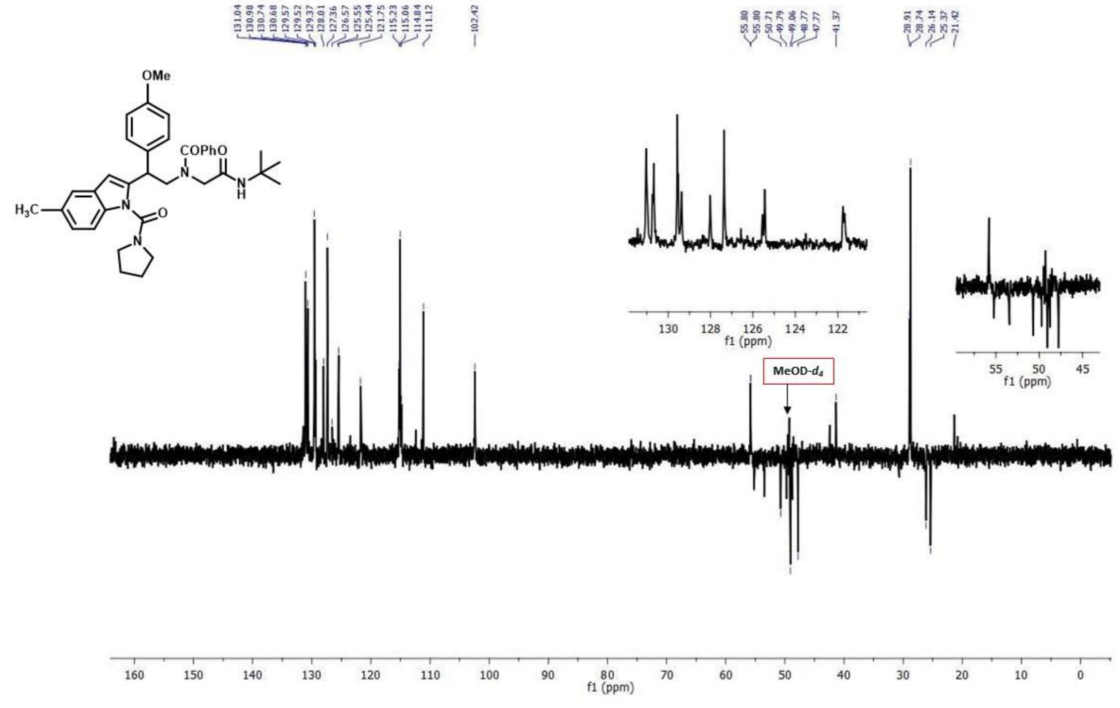

Figure S18. DEPT135 NMR of **4l** (MeOD-d_4.100 MHz, mixture of rotamers).

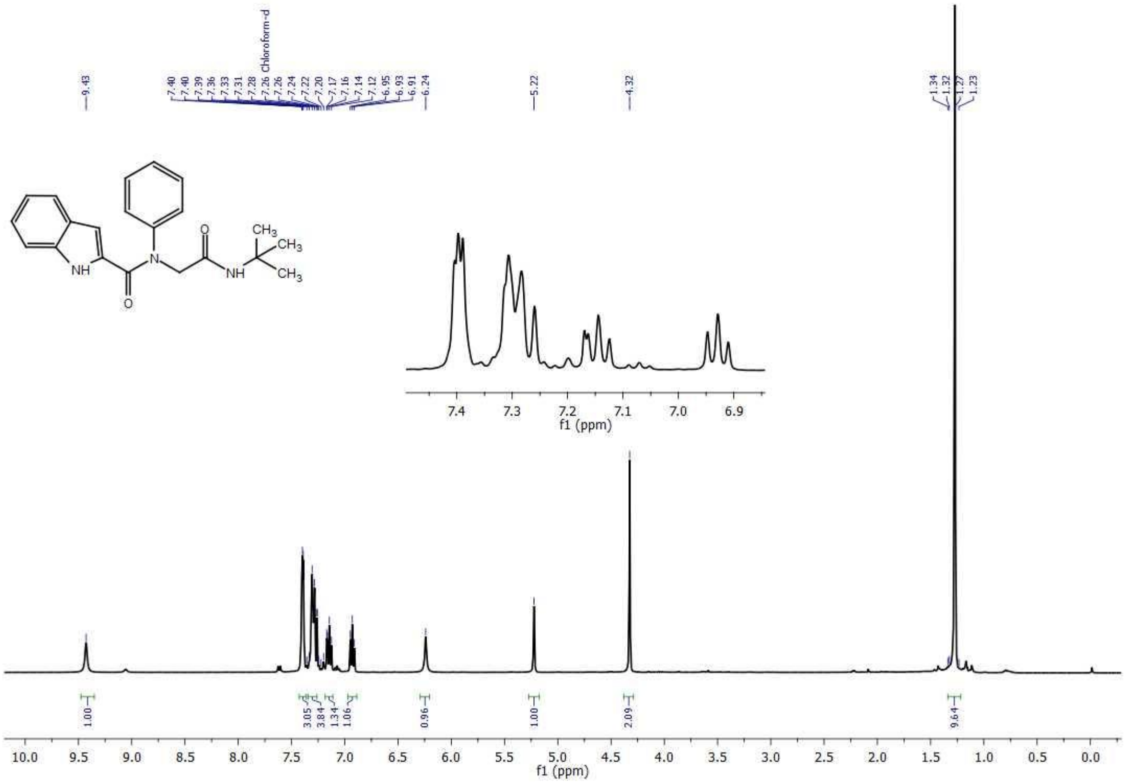

Figure S19. ^{1}H NMR of **6a** ($CDCl_3$, 400 MHz).

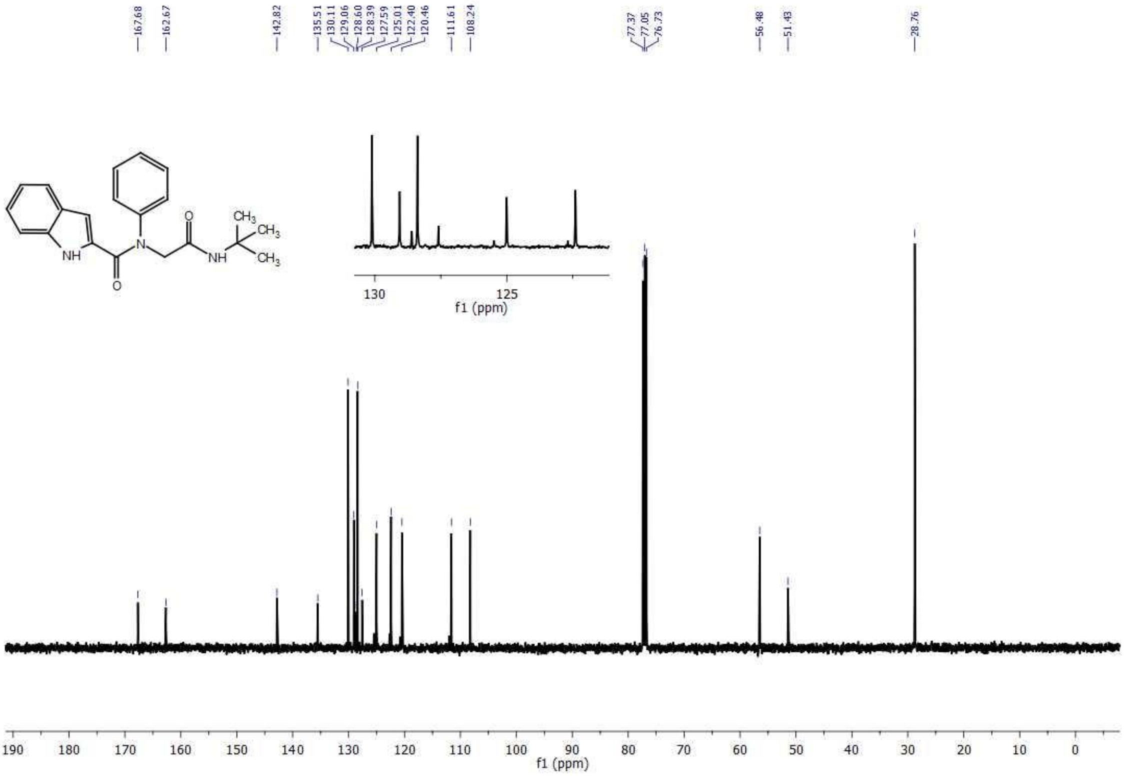

Figure S20. ^{13}C NMR of **6a** ($CDCl_3$, 100 MHz).

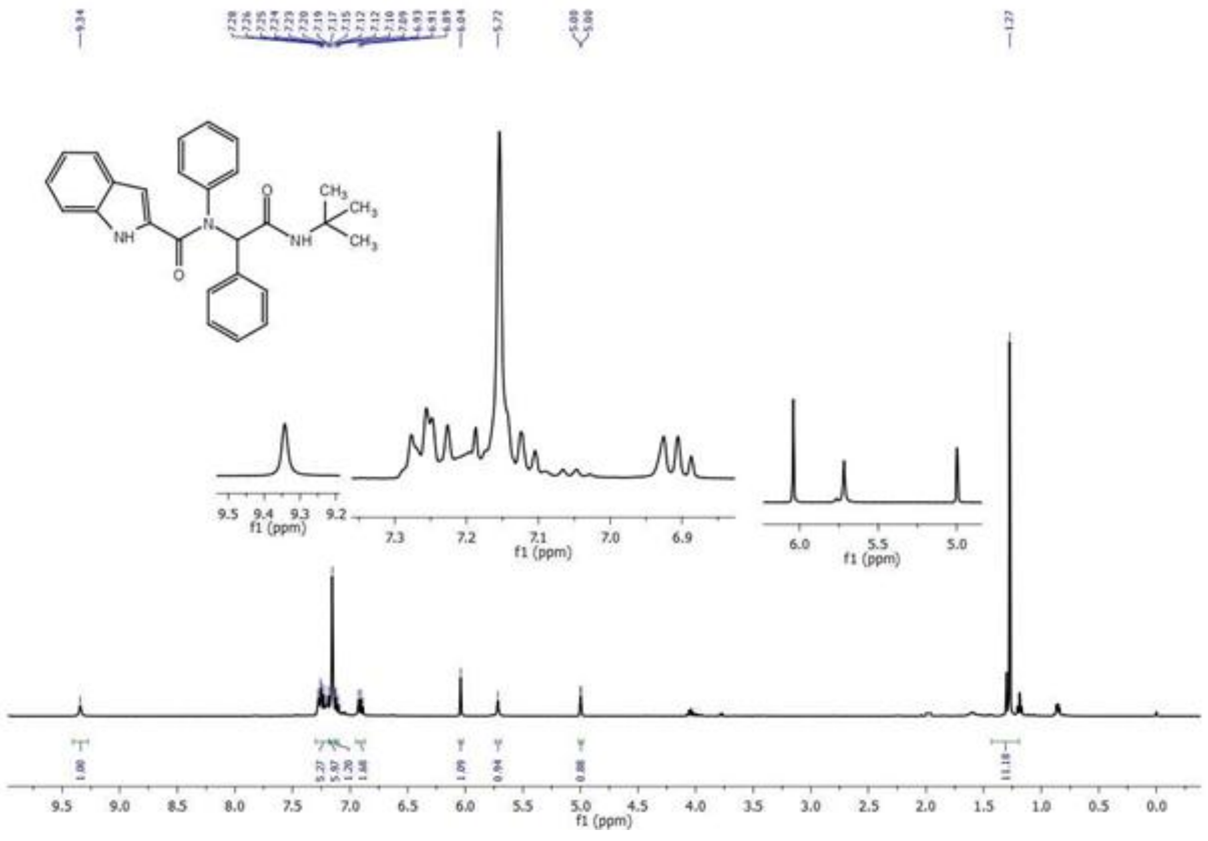

Figure S21. [13]H NMR of **6b** ($CDCl_3$, 400 MHz, *presence of rotamers*).

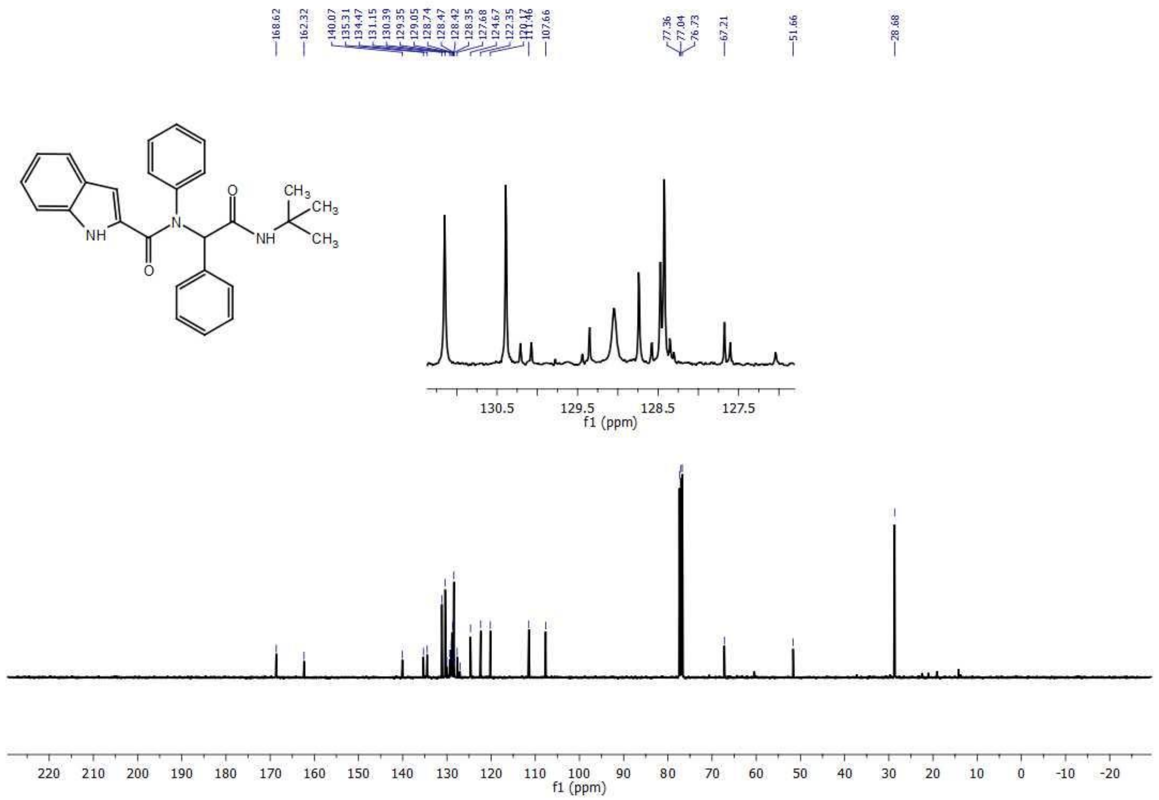

Figure S22. ^{13}C NMR of **6b** ($CDCl_3$,100 MHz, *presence of rotamers*).

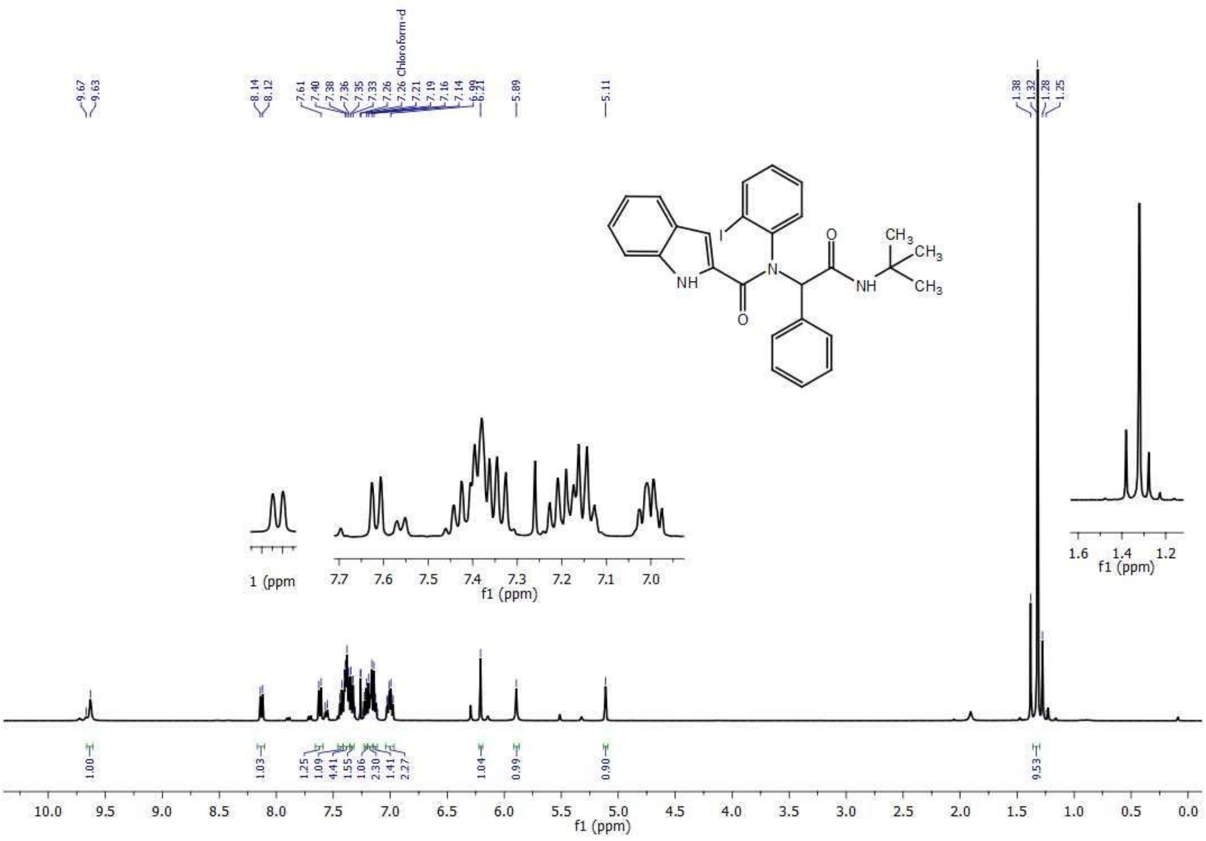

Figure S23. ^{13}H NMR of **6c** ($CDCl_3$, 400 MHz, mixture of rotamers).

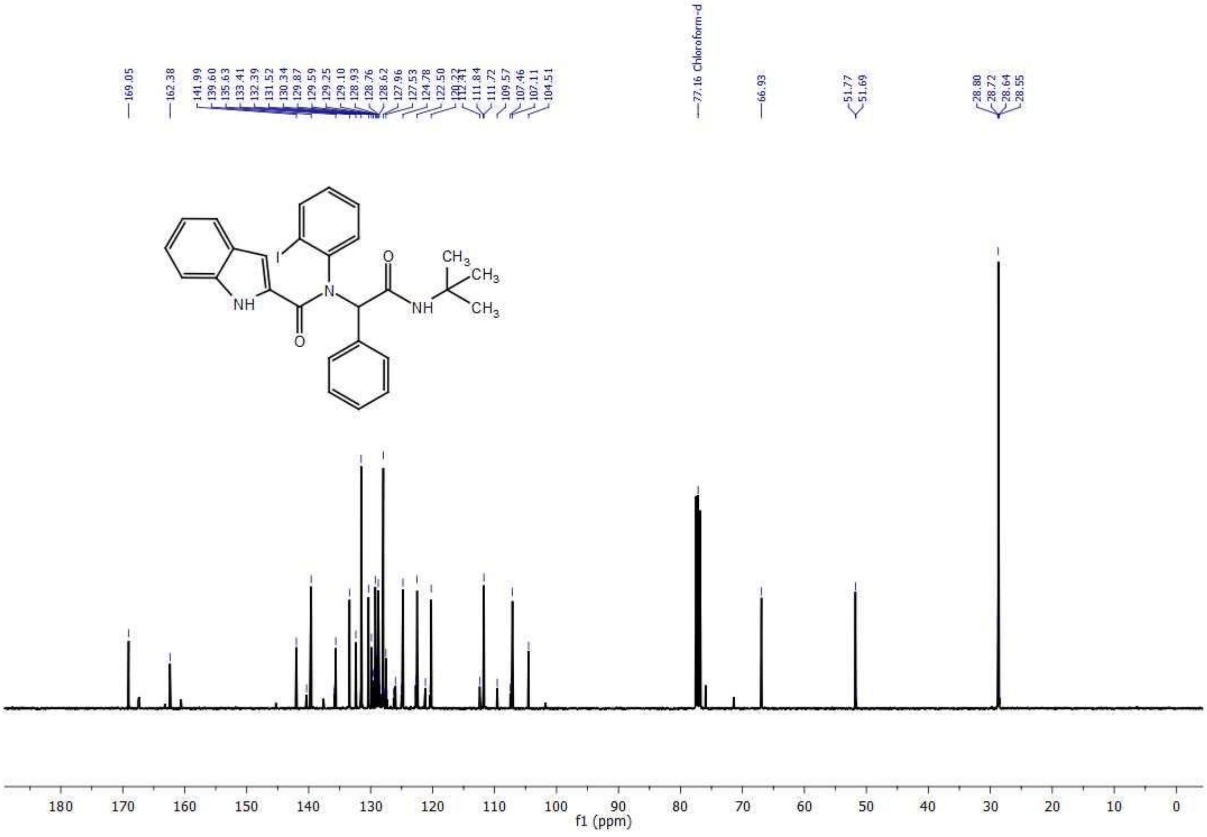

Figure S24. ^{13}C NMR of **6c** ($CDCl_3$, 100 MHz, mixture of rotamers).

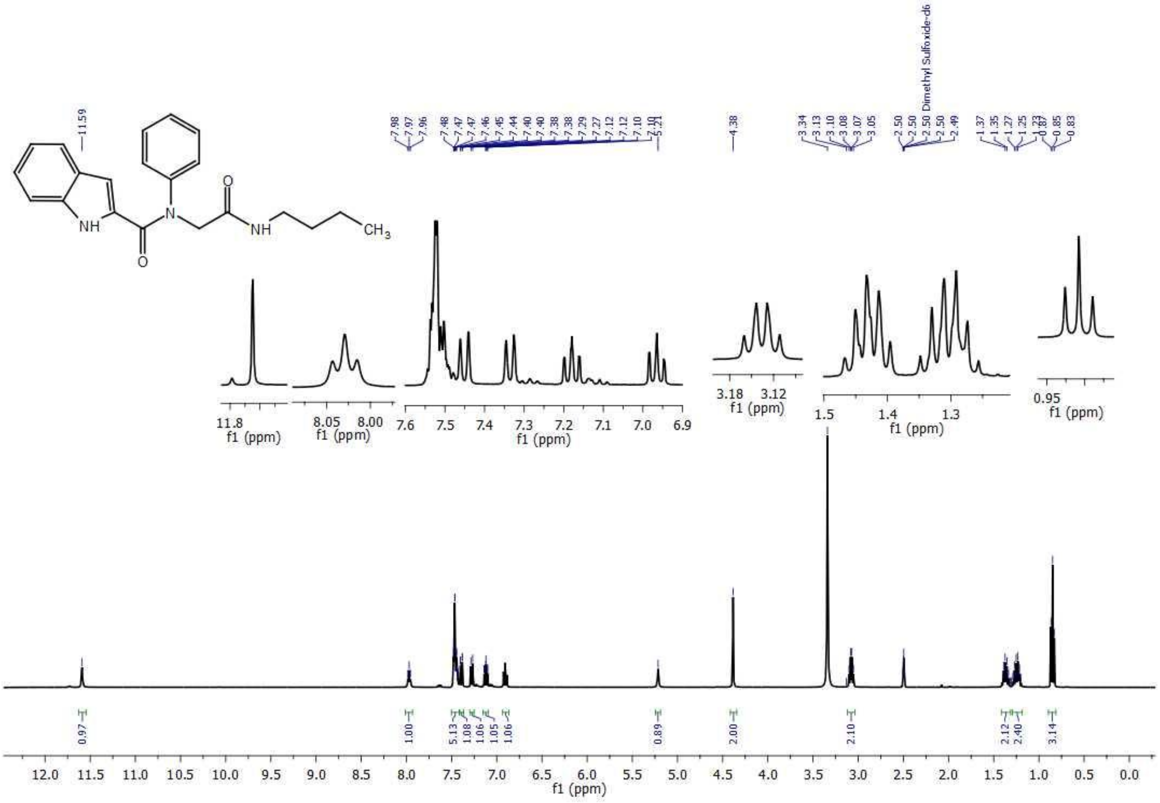

Figure S25. ^{1}H NMR of **6d** (DMSO-d_6, 400 MHz, *presence of rotamers*).

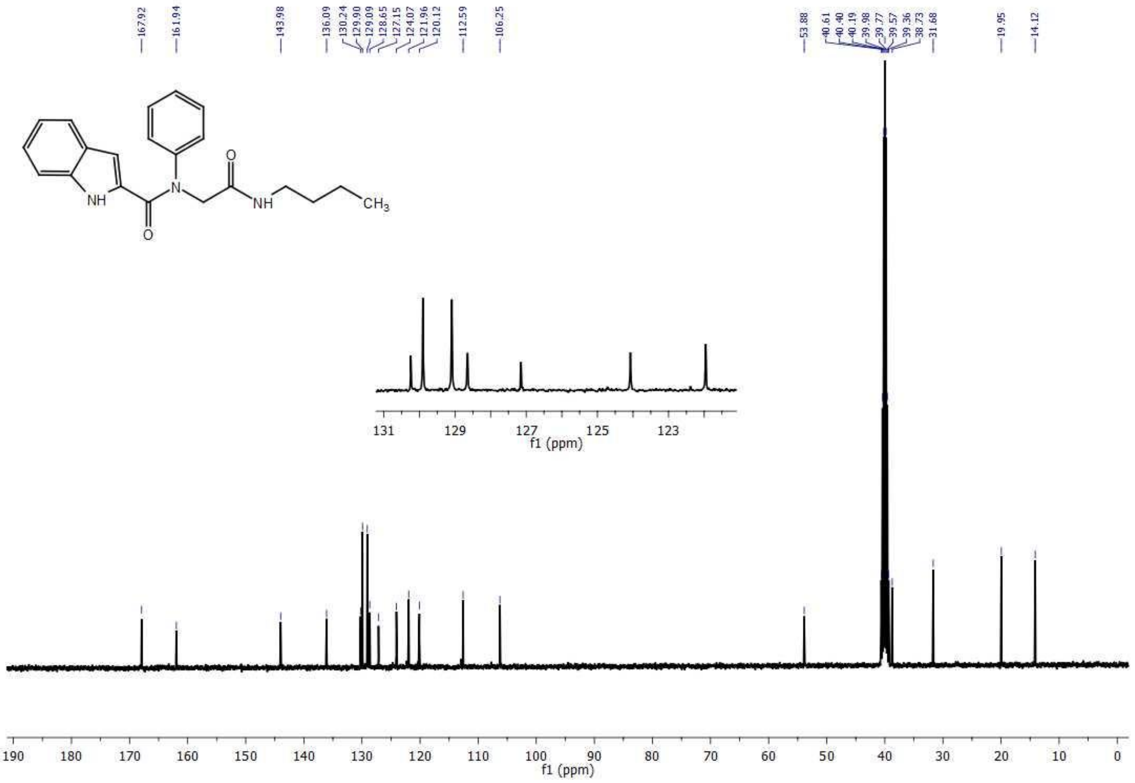

Figure S26. ^{13}C NMR of **6d** (DMSO-d_6,100 MHz, *presence of rotamers*).

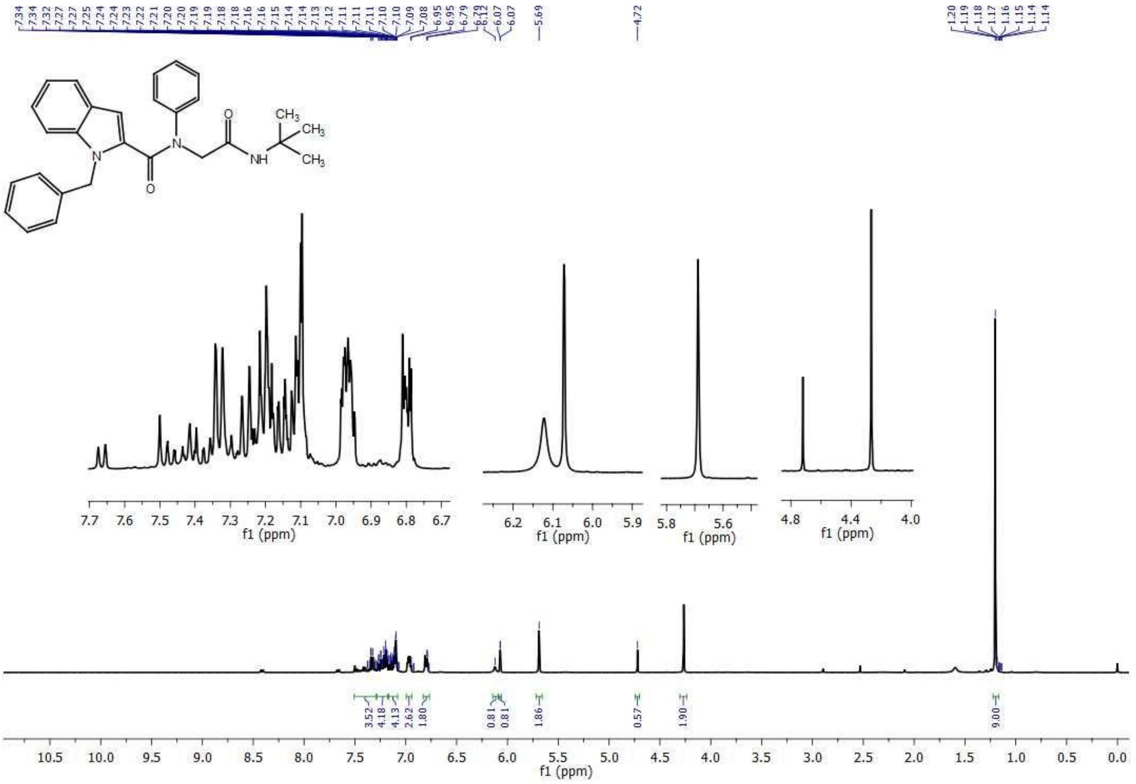

Figure S27. ^{1}H NMR of **6e** ($CDCl_3$, 400 MHz, mixture of rotamers).

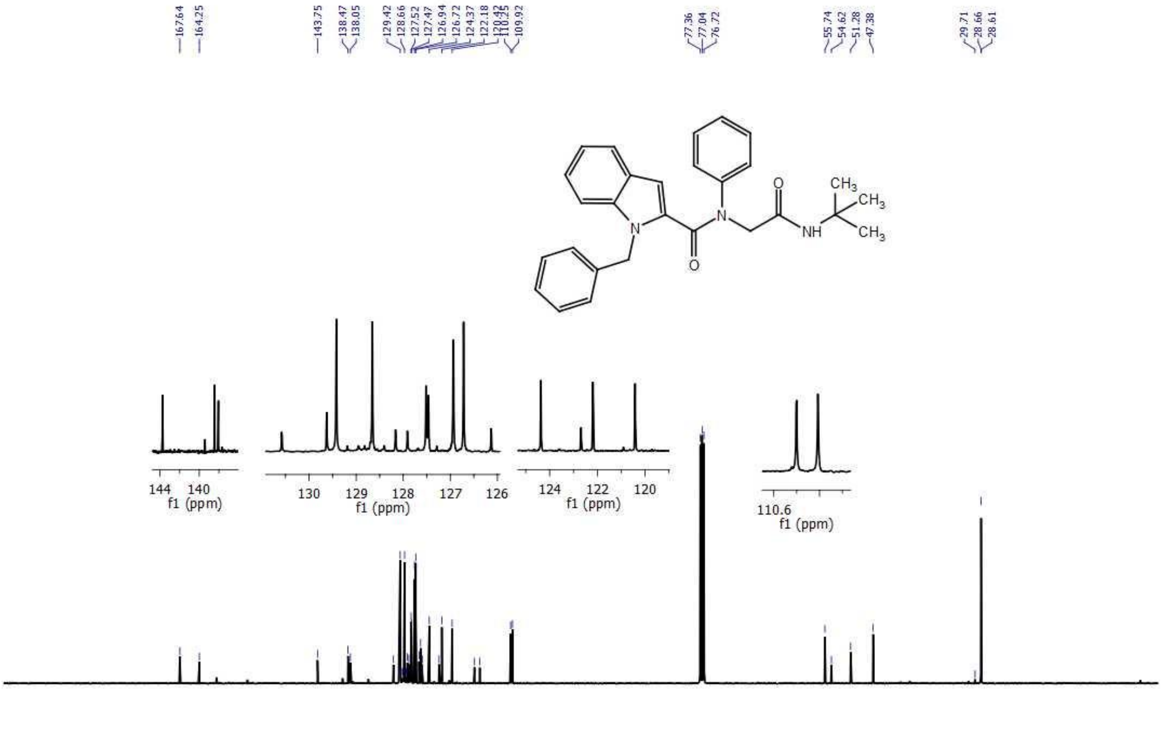

Figure S28. ^{13}C NMR of **6e** ($CDCl_3$, 100 MHz, mixture of rotamers).

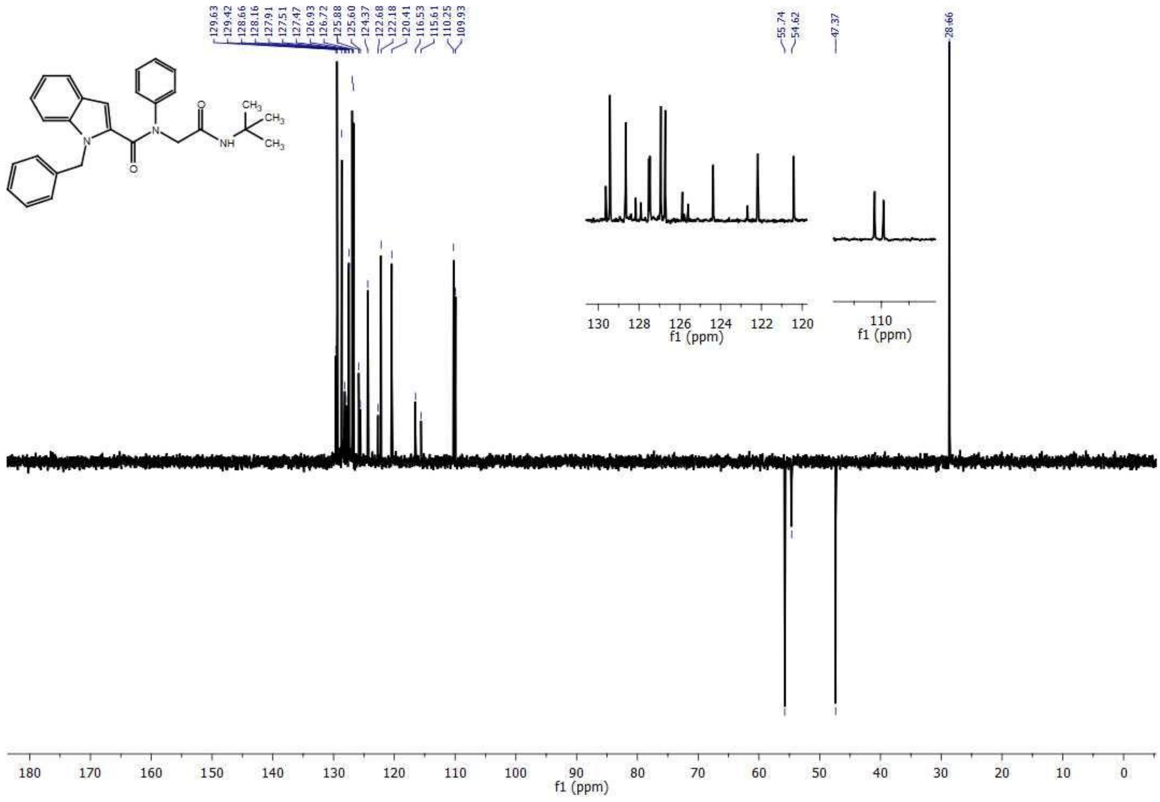

Figure S29. ^{13}C NMR of **6e** ($CDCl_3$, 100 MHz, *presence of rotamers*).

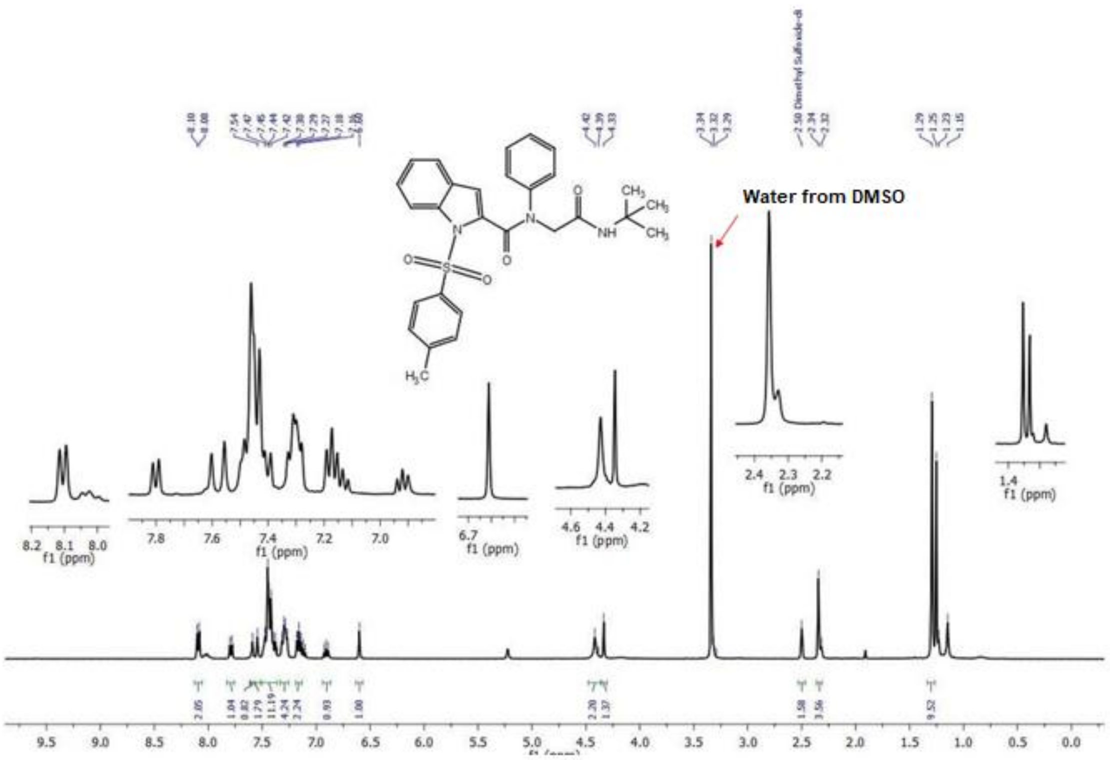

Figure S30. ^{1}H NMR of **6f** (DMSO-d_6, 400 MHz, *presence of rotamers*).

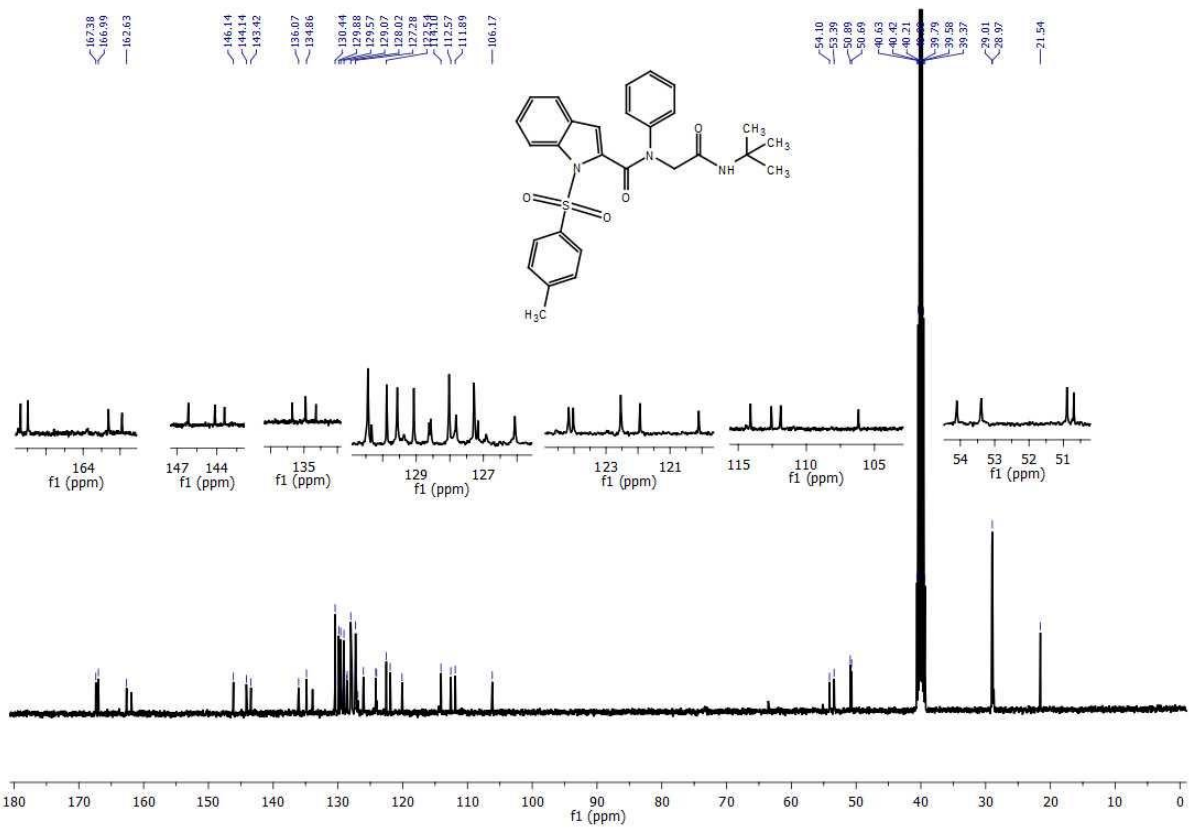

Figure S31. ^{13}C NMR of **6f** (DMSO-d_6, 100 MHz, *presence of rotamers*).

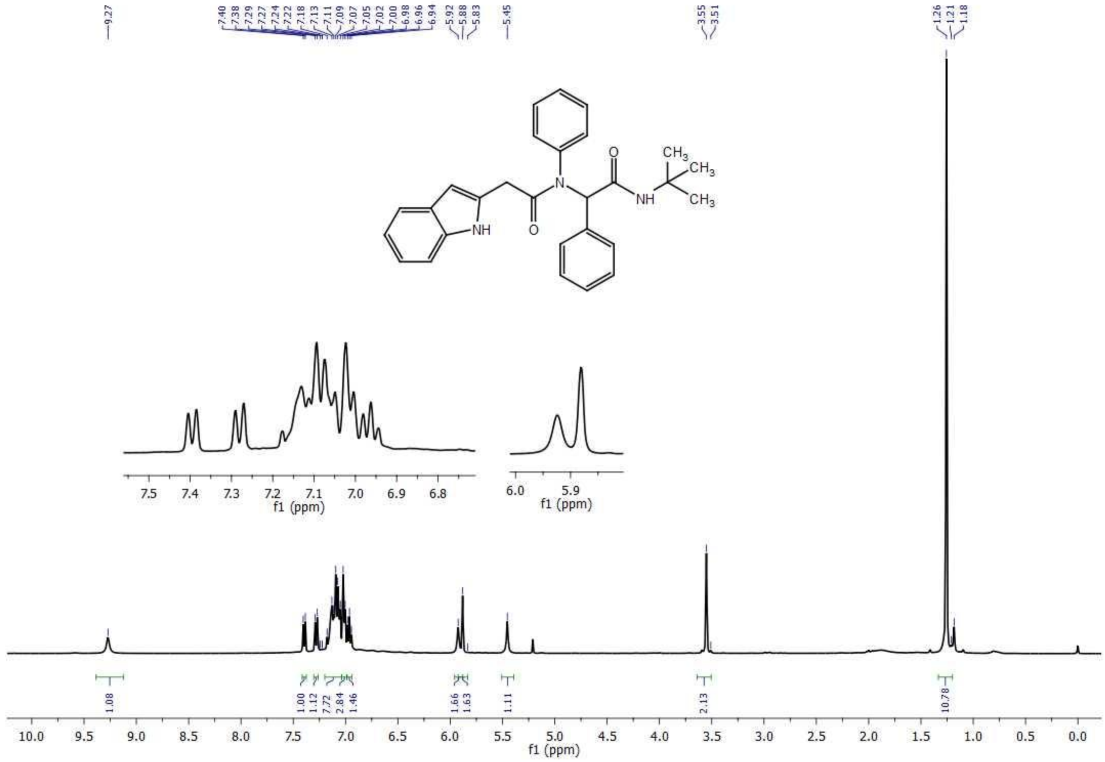

Figure S32. ^{1}H NMR of **6g** ($CDCl_3$, at 400 MHz, *presence of rotamers*).

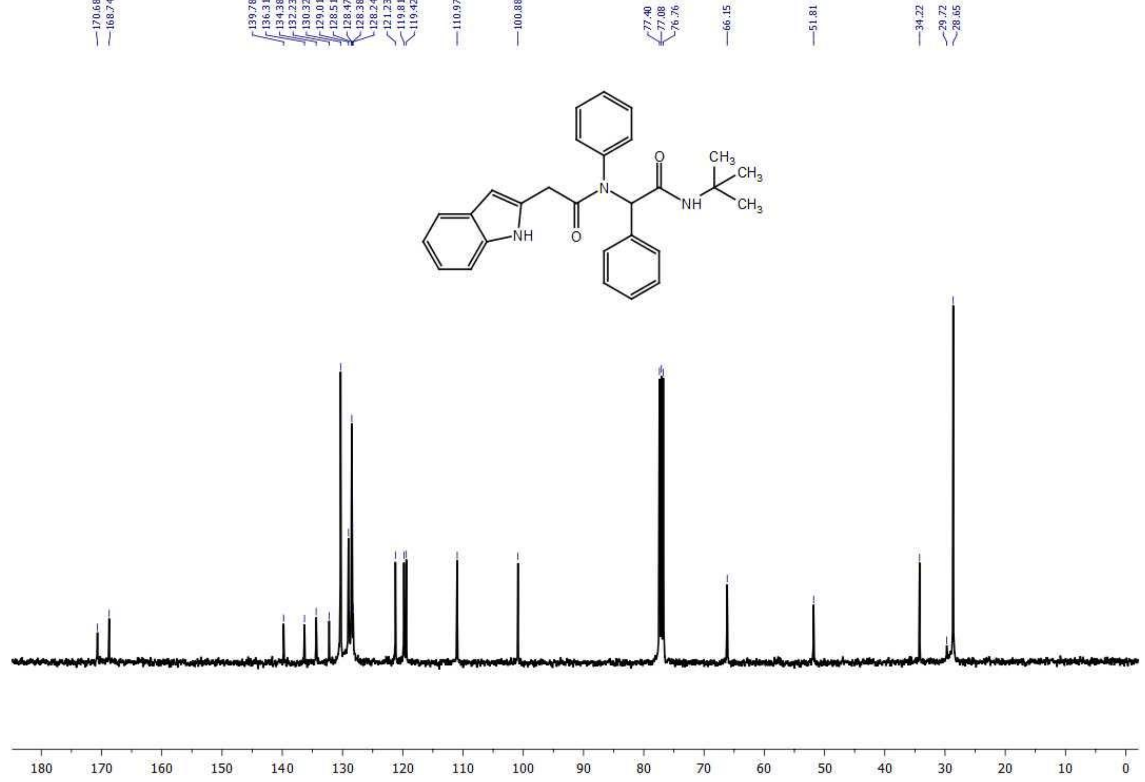

Figure S33. ^{1}H NMR of **6g** in $CDCl_3$ (400 MHz, *presence of rotamers*).

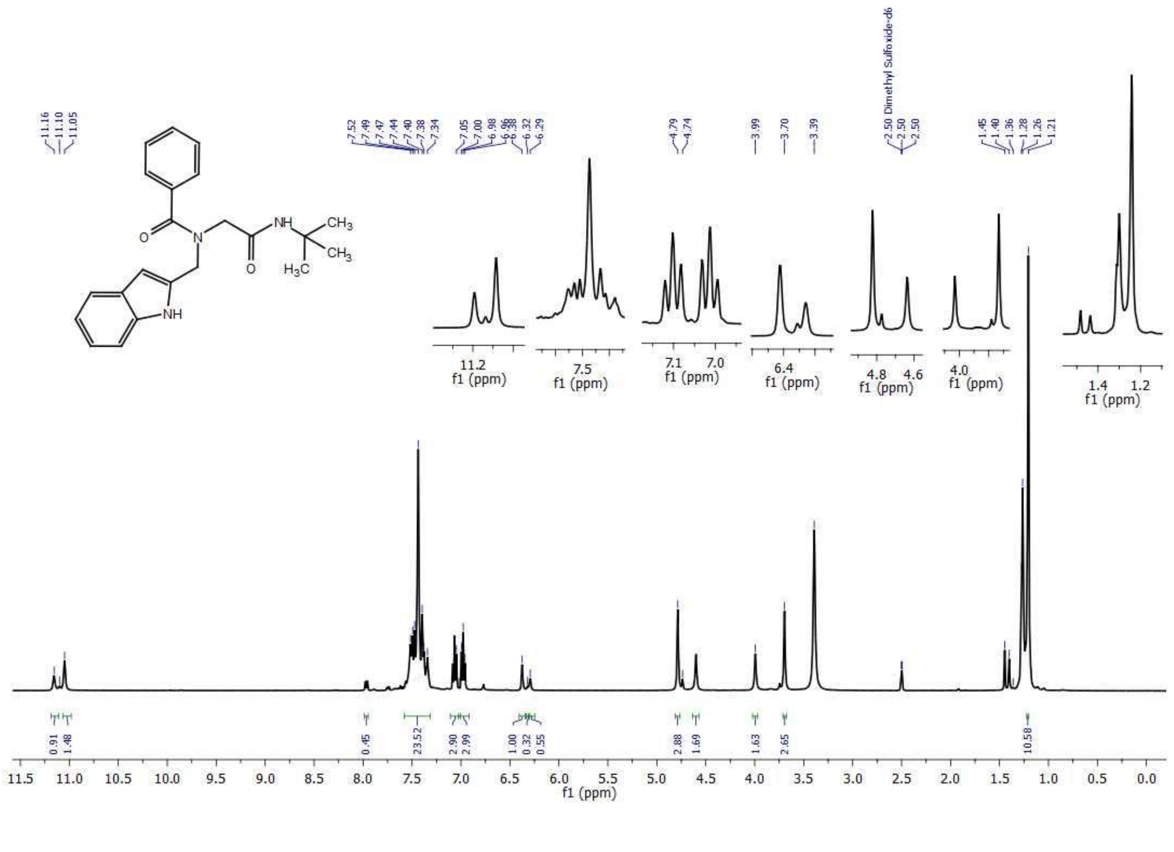

Figure S34. ^{1}H NMR of **9a** (DMSO-d_6, 400 MHz, *presence of rotamers*).

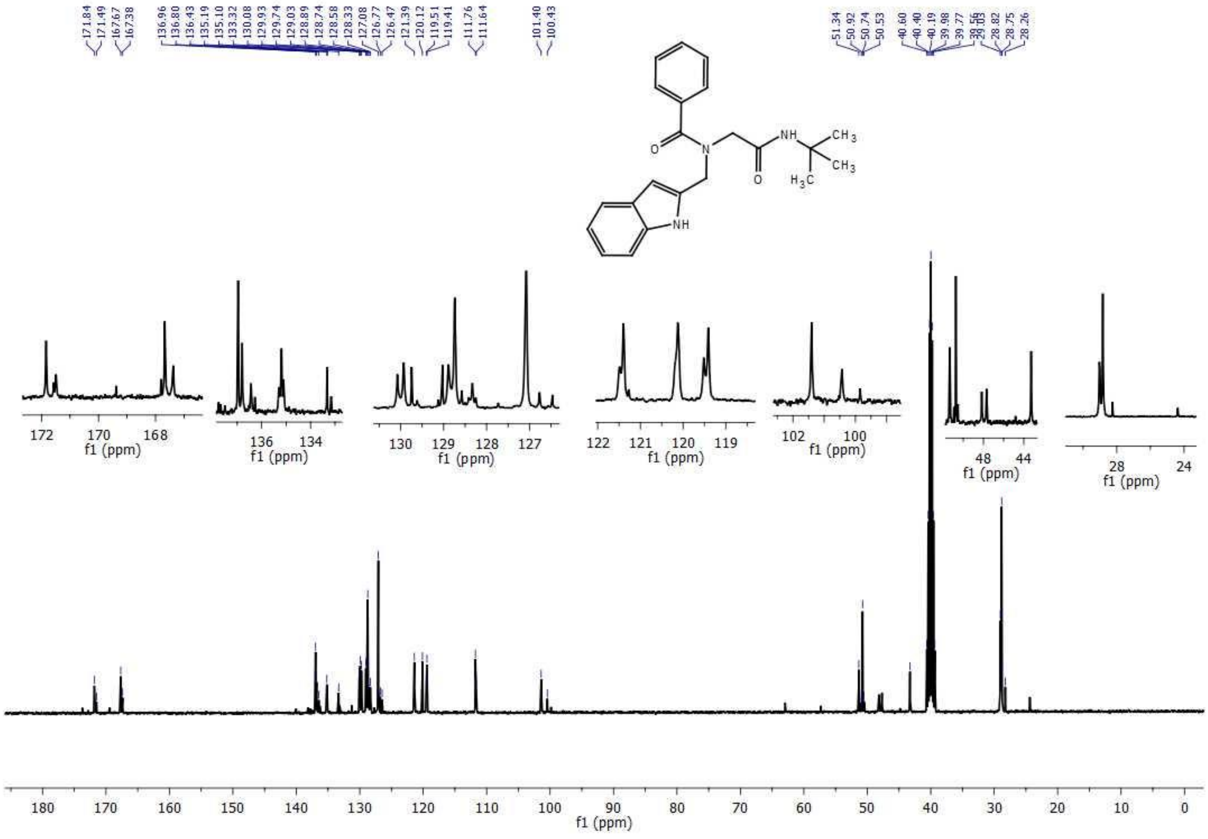

Figure S35. ^{13}C NMR of **9a** (DMSO-d_6, 100 MHz, mixture of rotamers).

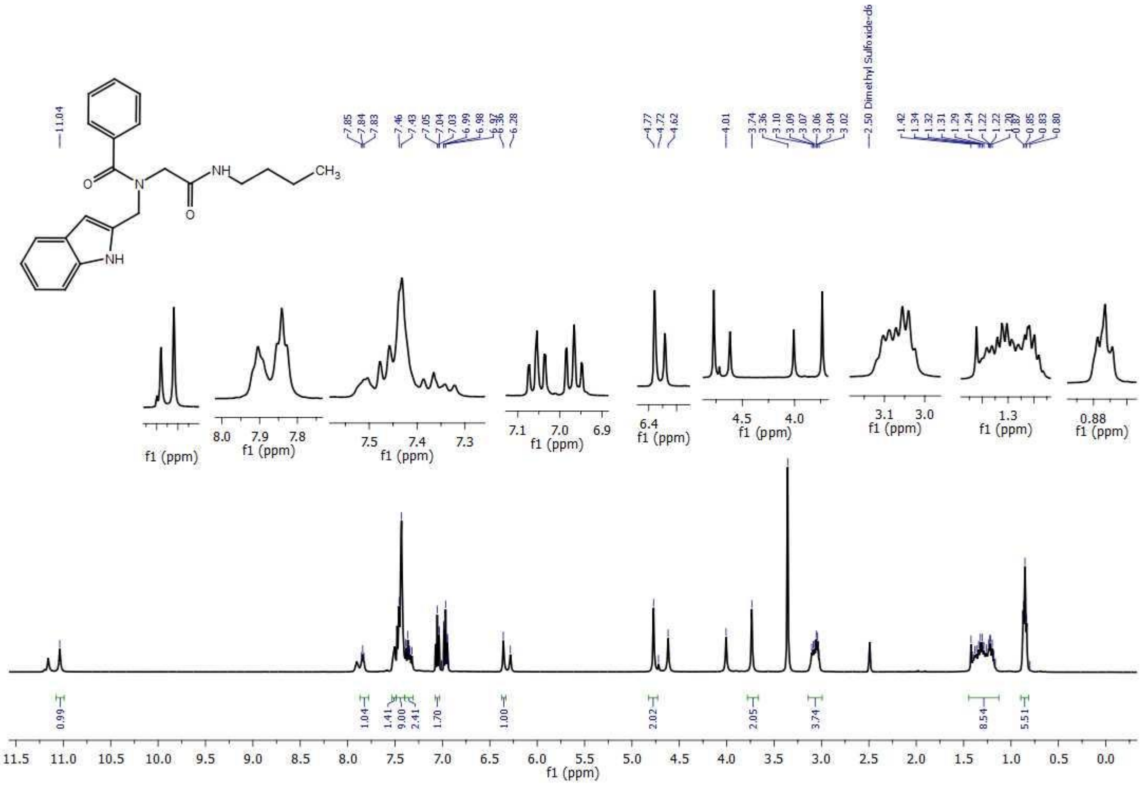

Figure S36. ^{1}H NMR of **9d** in DMSO-d_6, 400 MHz, mixture of rotamers).

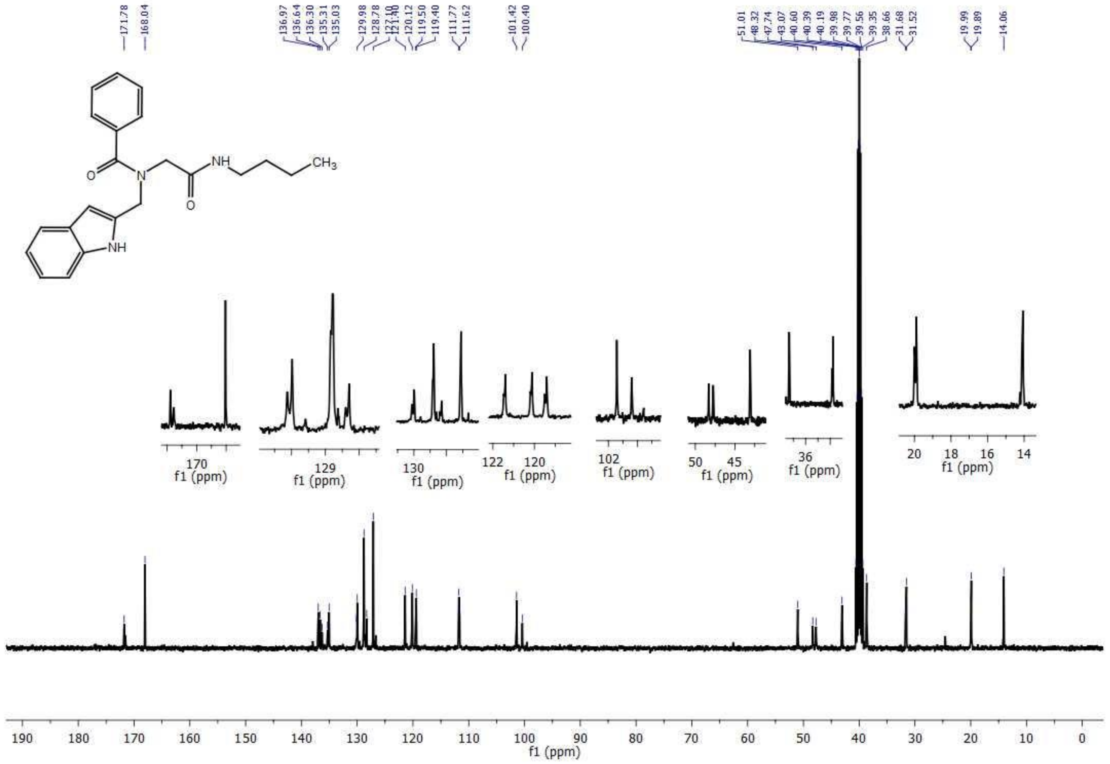

Figure S37. ^{13}C NMR of **9d** (DMSO-d_6, 100 MHz, mixture of rotamers).

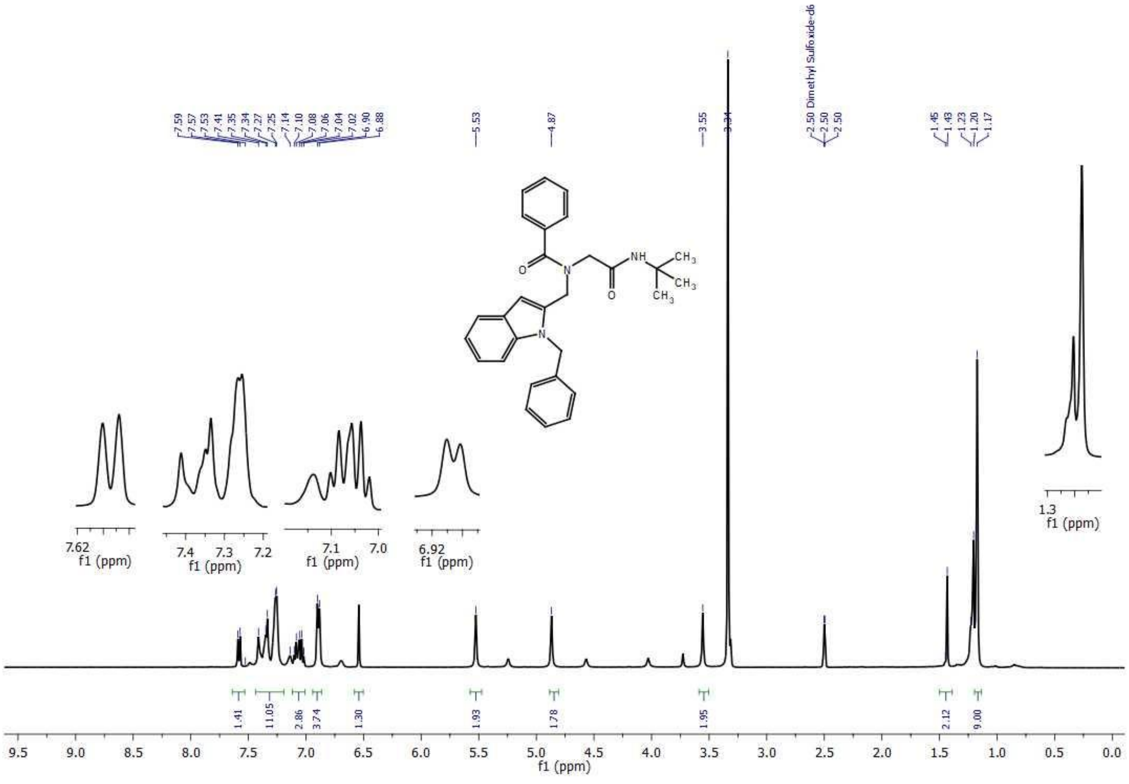

Figure S38. ^{1}H NMR of **9b** (DMSO-d_6, 400 MHz, mixture of rotamers).

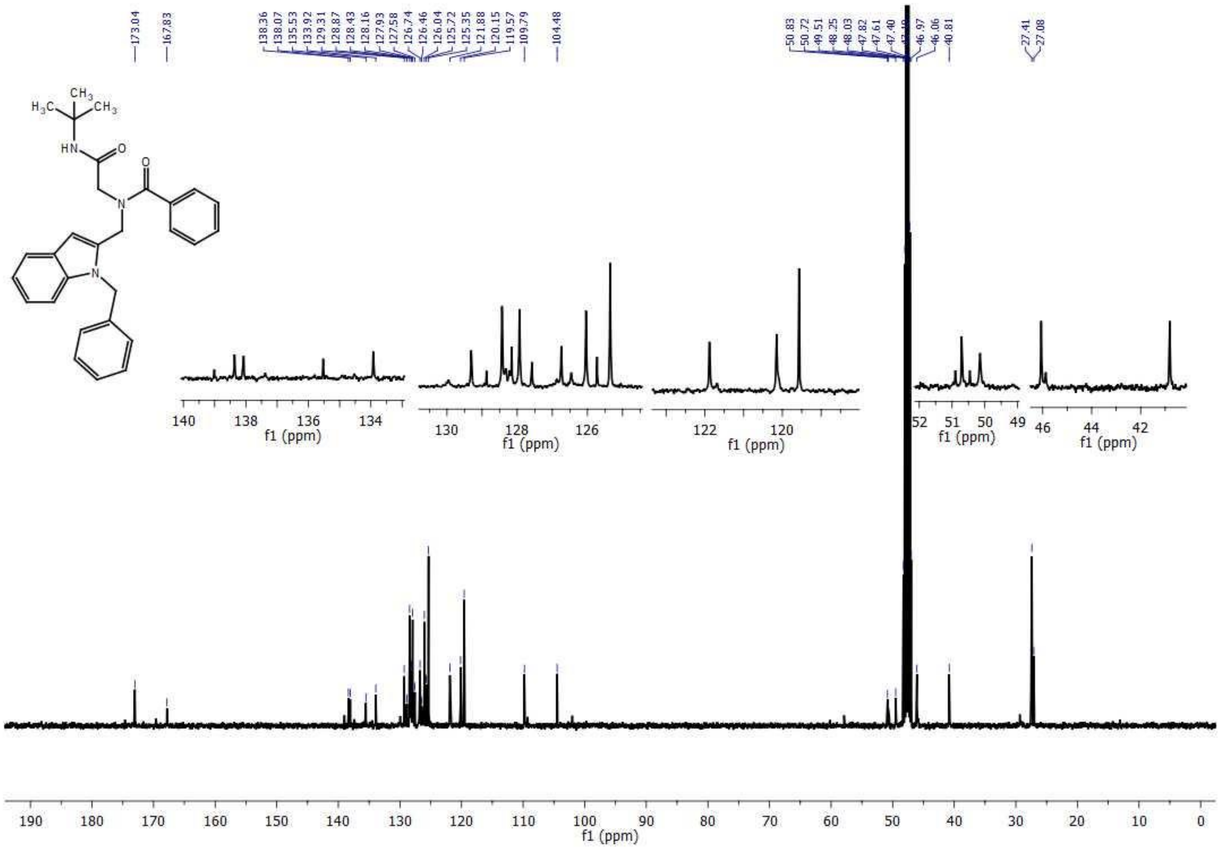

Figure S39. ^{1}H NMR of **9b** (DMSO-*d*$_6$, 100 MHz, mixture of rotamers).

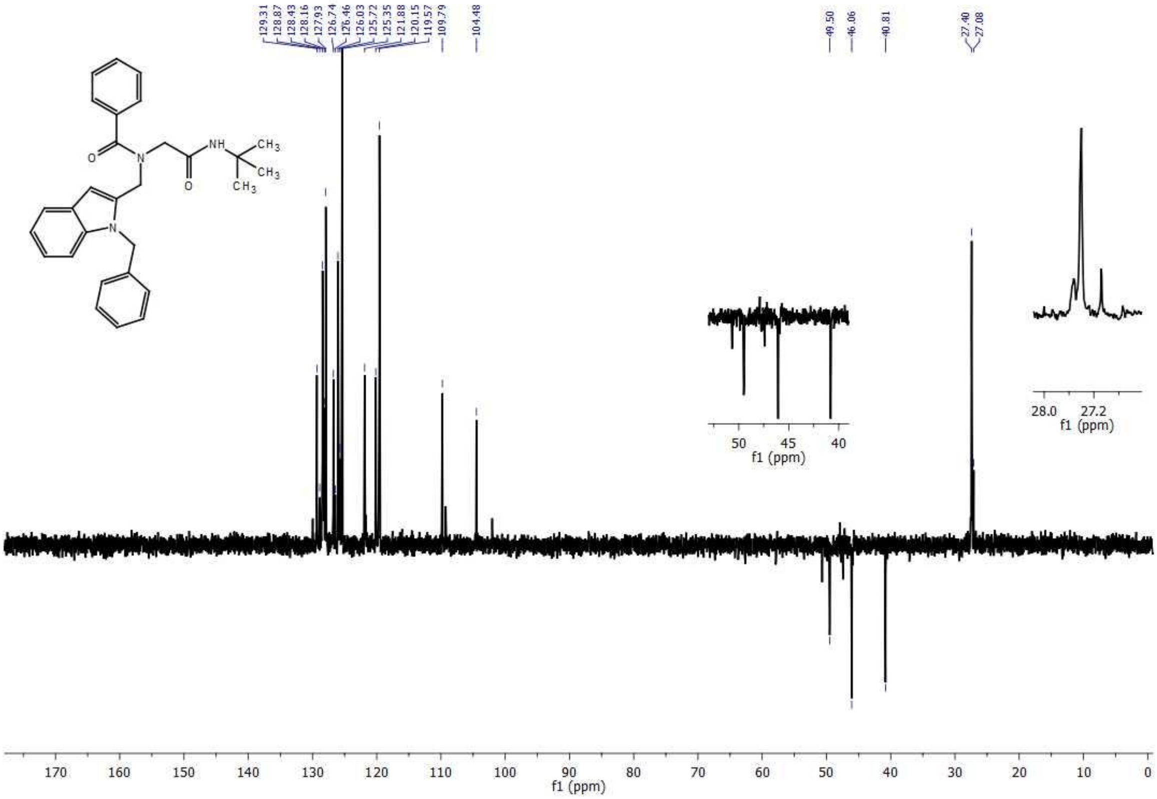

Figure S40. DEPT135 NMR of **9b** (DMSO-*d*$_6$, 100 MHz, mixture of rotamers).

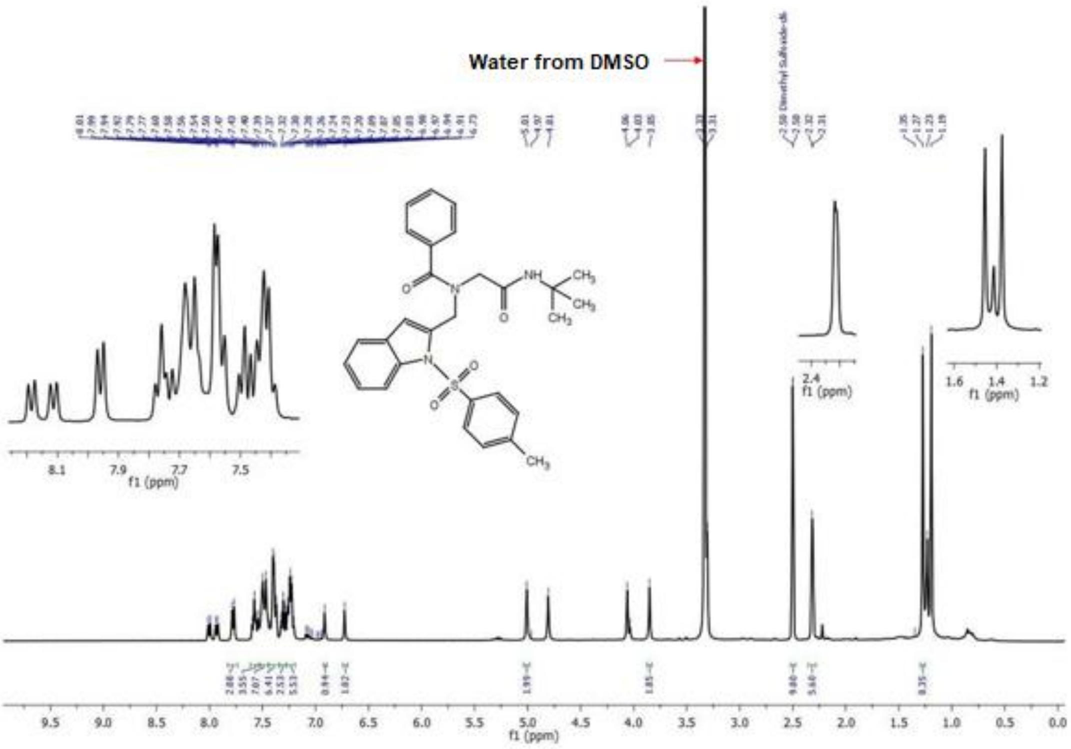

Figure S41. ^{1}H NMR of **9c** (DMSO-*d*$_6$, 400 MHz, *presence of rotamers*).

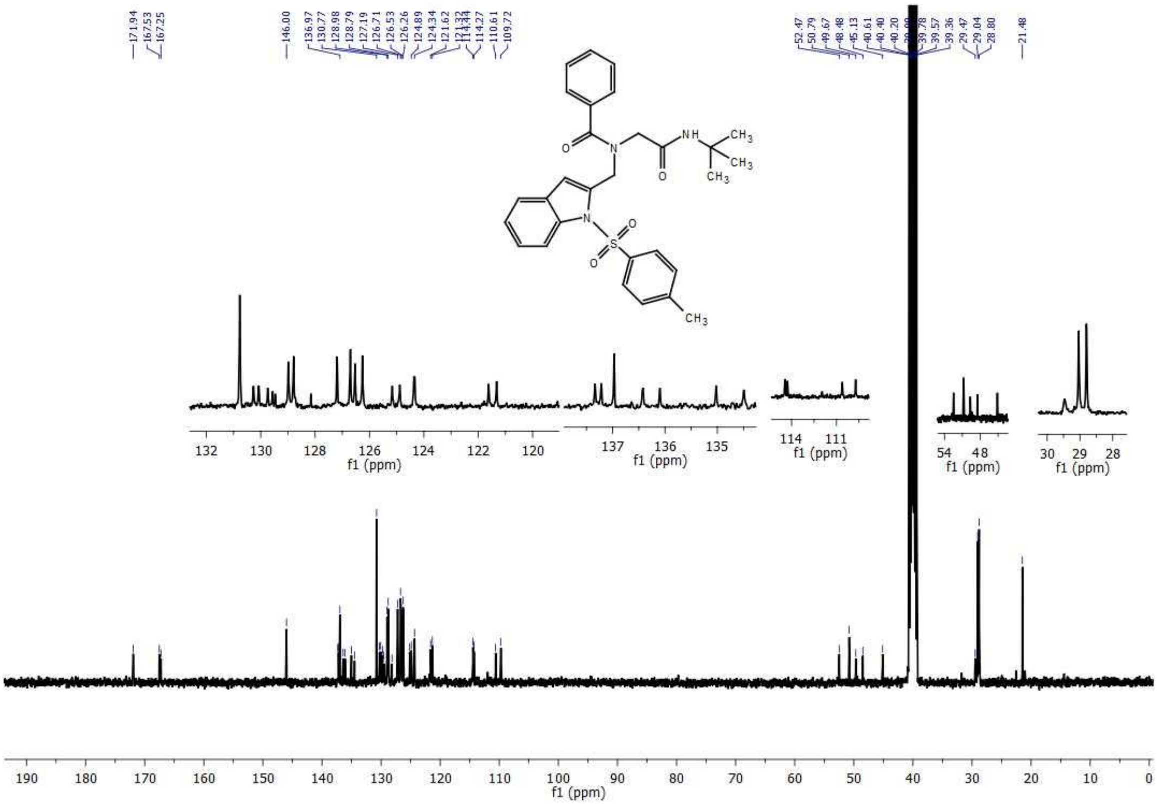

Figure S42. ^{13}C NMR of **9c** (DMSO-*d*$_6$, 100 MHz, mixture of rotamers).

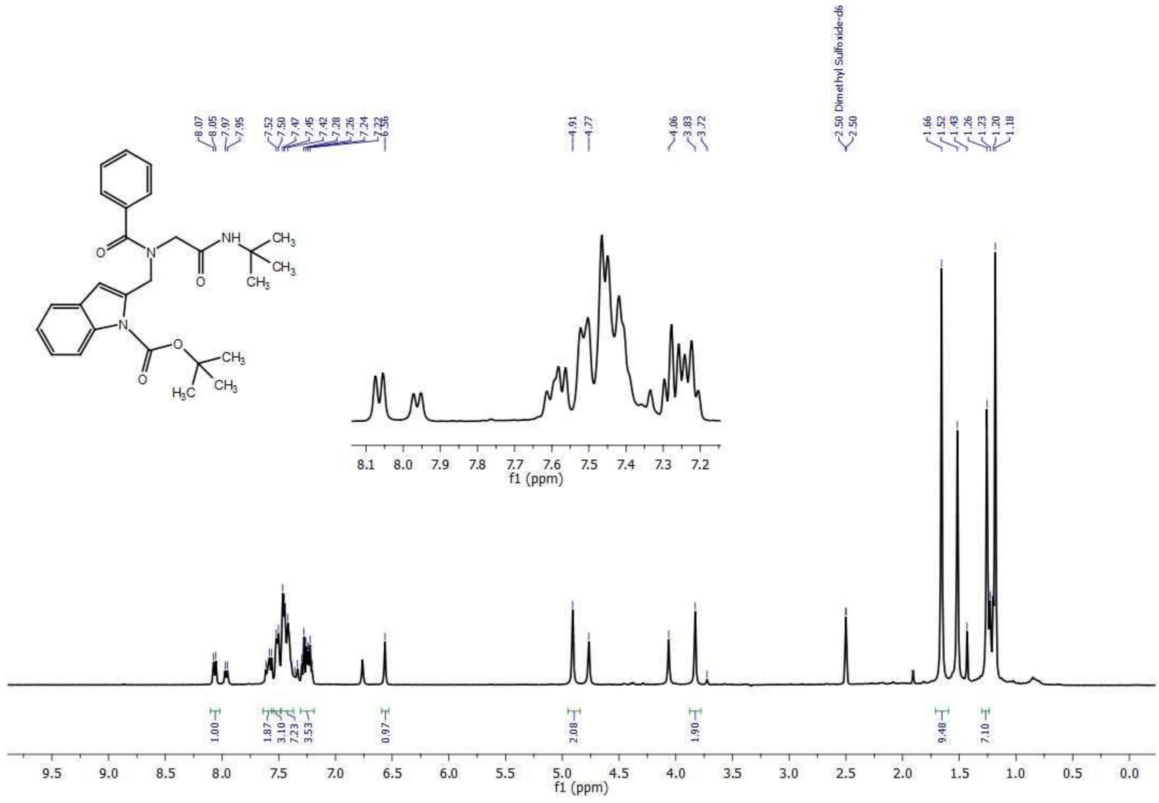

Figure S43. ^{1}H NMR of **9e** (DMSO-d_6, 400 MHz, mixture of amide and Boc rotamers) with water signal suppression.

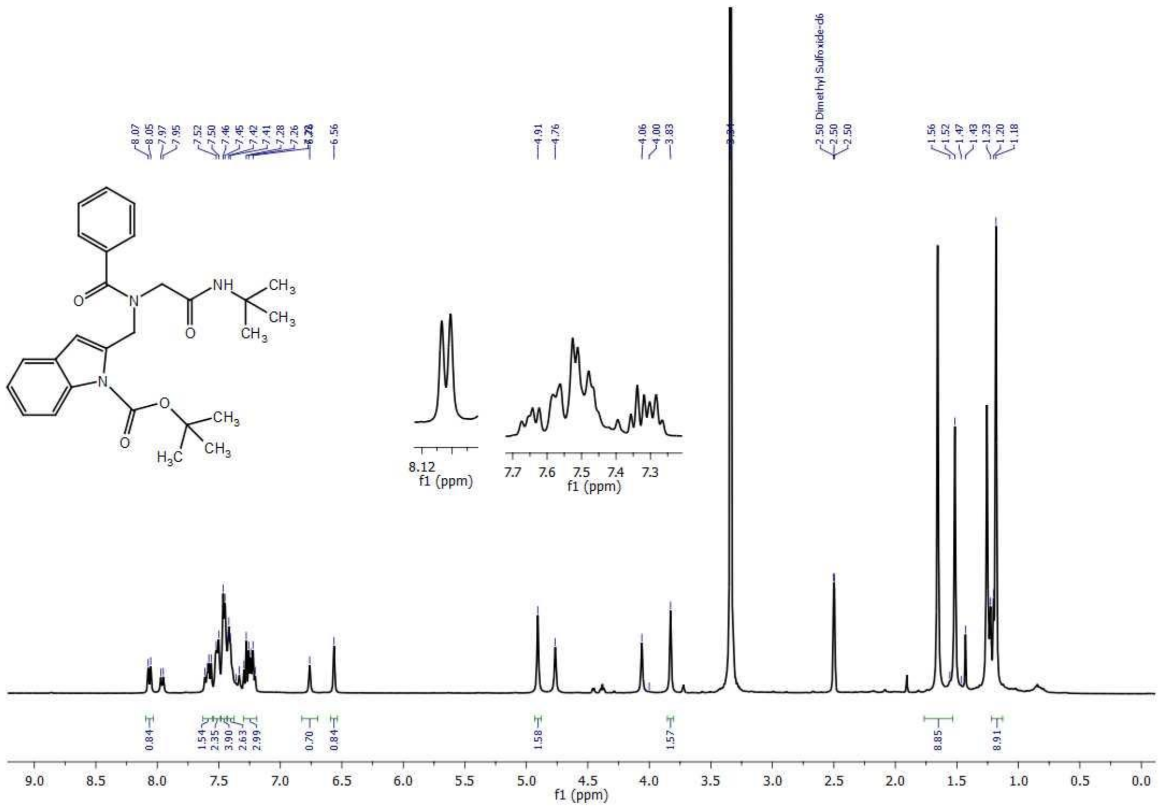

Figure S44. ^{1}H NMR of **9e** (DMSO-d_6, 400 MHz, mixture of amide and Boc rotamers).

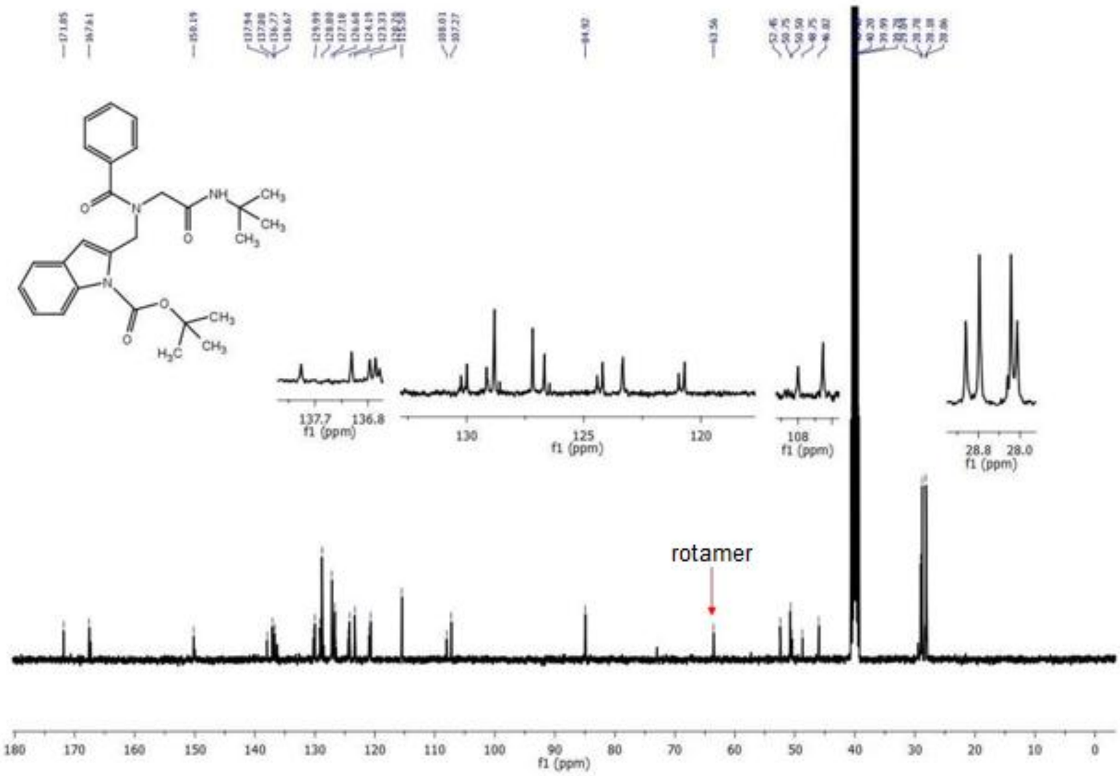

Figure S45. ^{13}C NMR of **9e** (DMSO-d_6, 400 MHz, mixture of amide and Boc rotamers).

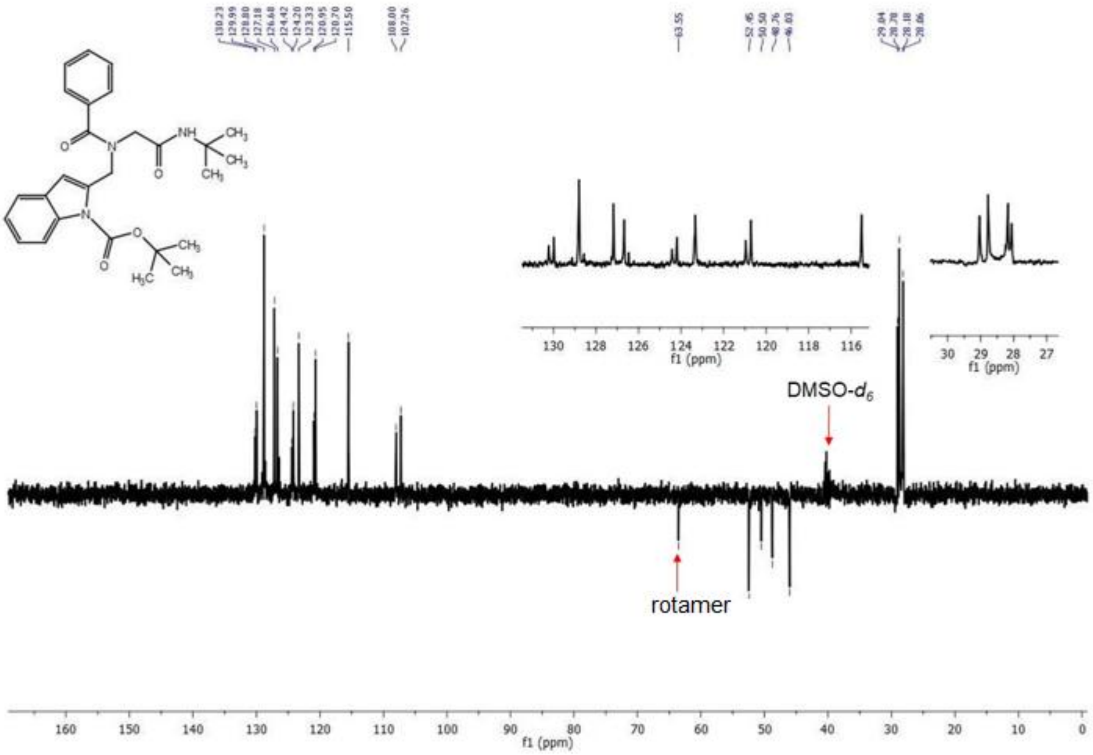

Figure S46. DEPT135 NMR of **9e** in DMSO-d_6 (400 MHz, mixture of amide and Boc rotamers).

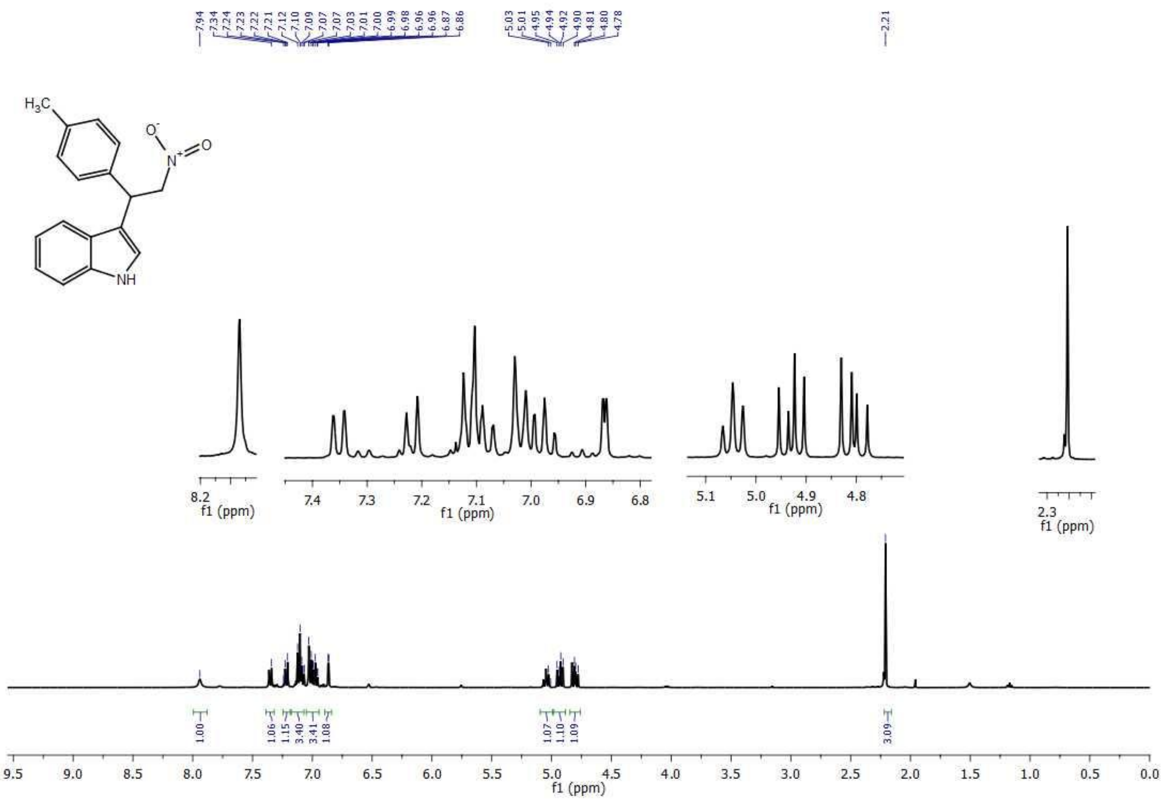

Figure S47. ^{1}H NMR of **10a** ($CDCl_3$, 400 MHz).

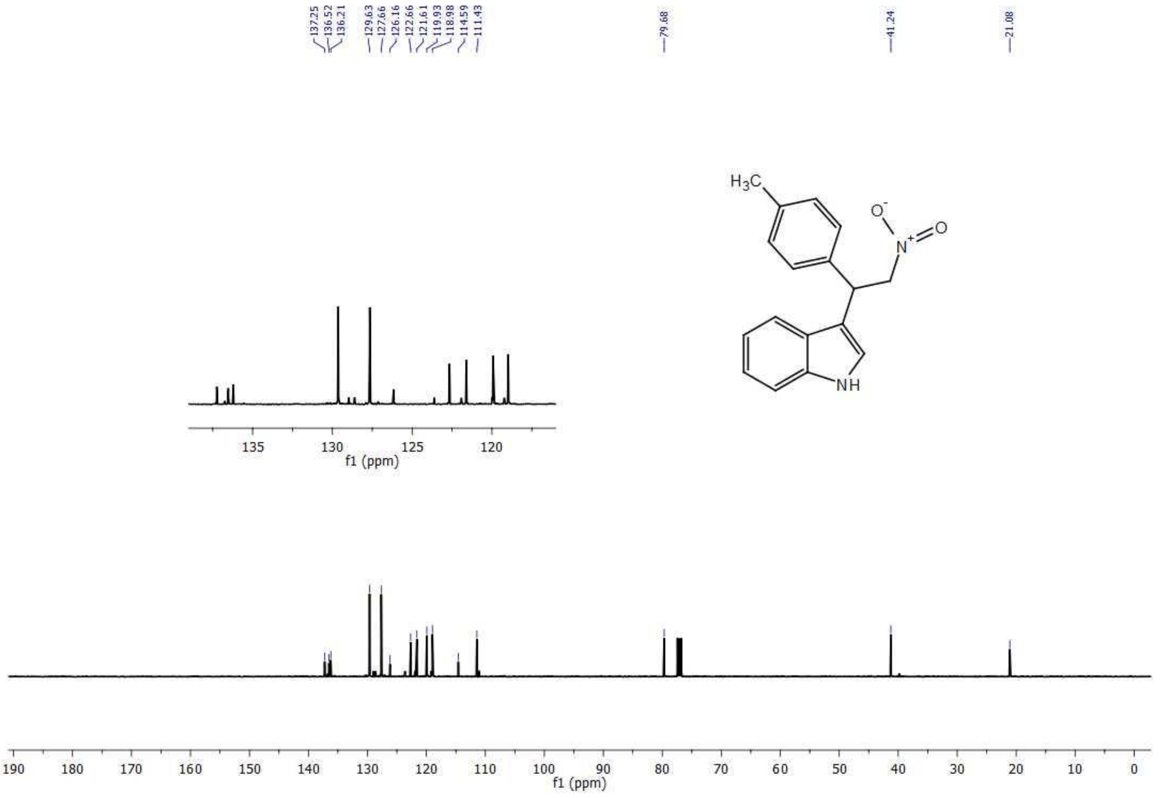

Figure S48. ^{13}C NMR of **10a** ($CDCl_3$, 100 MHz).

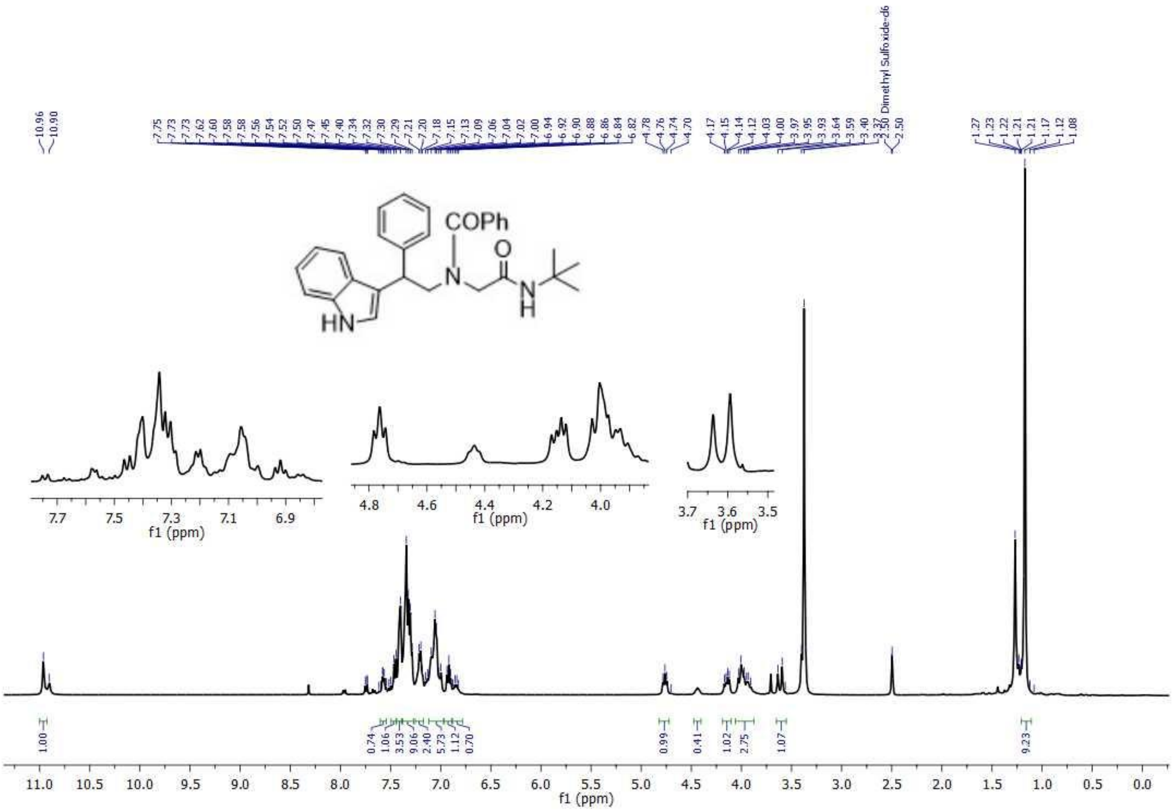

Figure S49. ^{1}H NMR of **12a** (DMSO-d_6, 400 MHz, presence of rotamers).

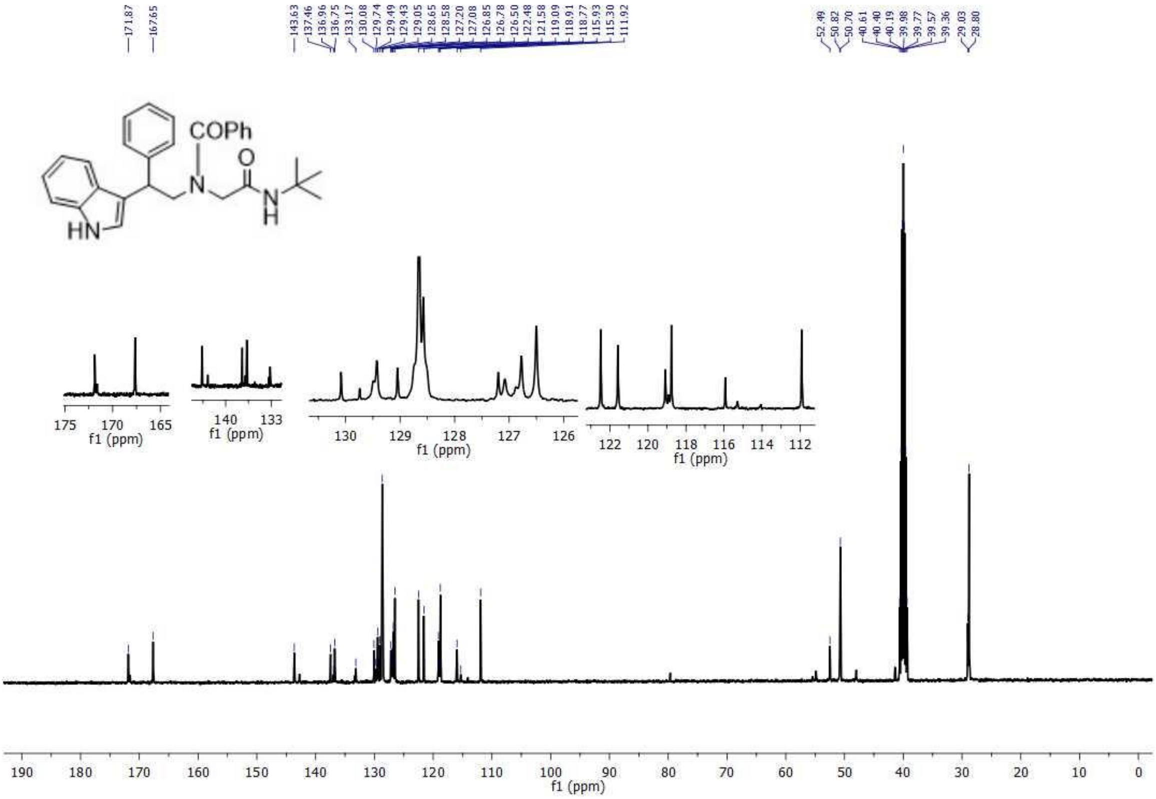

Figure S50. ^{13}C NMR of **12a** (DMSO-d_6, 400 MHz, presence of rotamers).

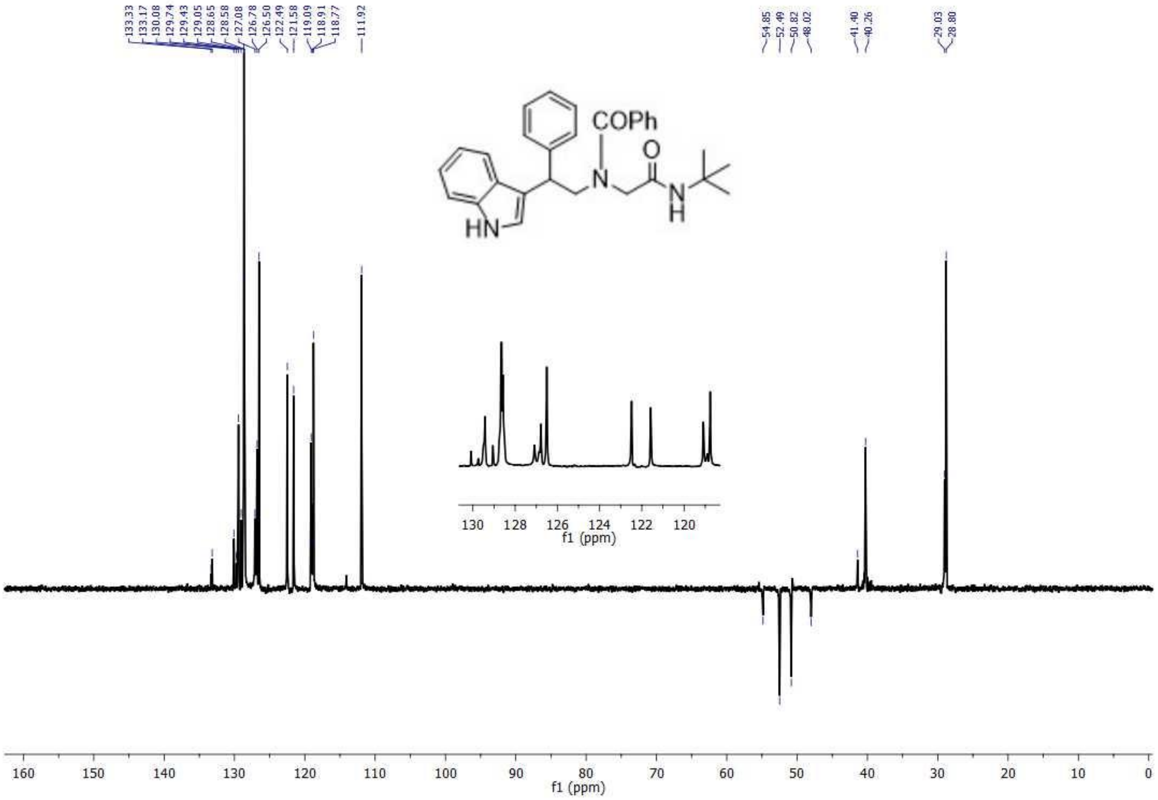

Figure S51. DEPT135 NMR of **12a** (DMSO-d_6, 400 MHz, mixture of rotamers).

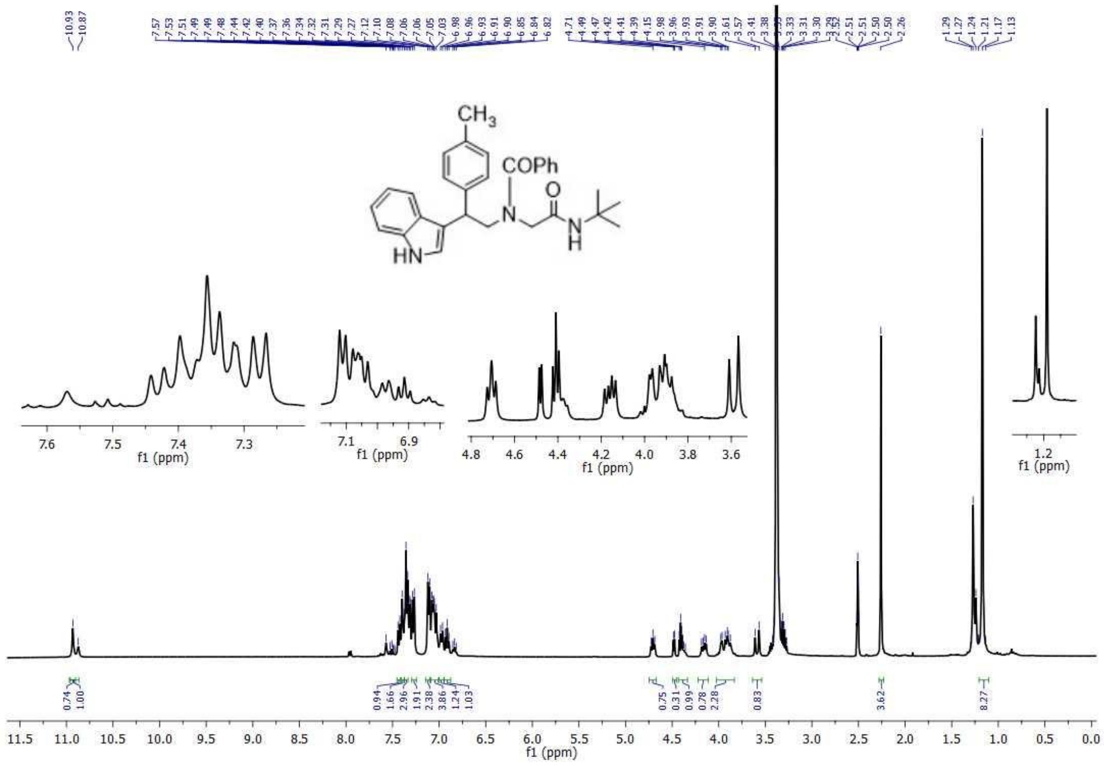

Figure S52. ^{1}H NMR of **12b** (DMSO-d_6, 400 MHz, mixture of rotamers).

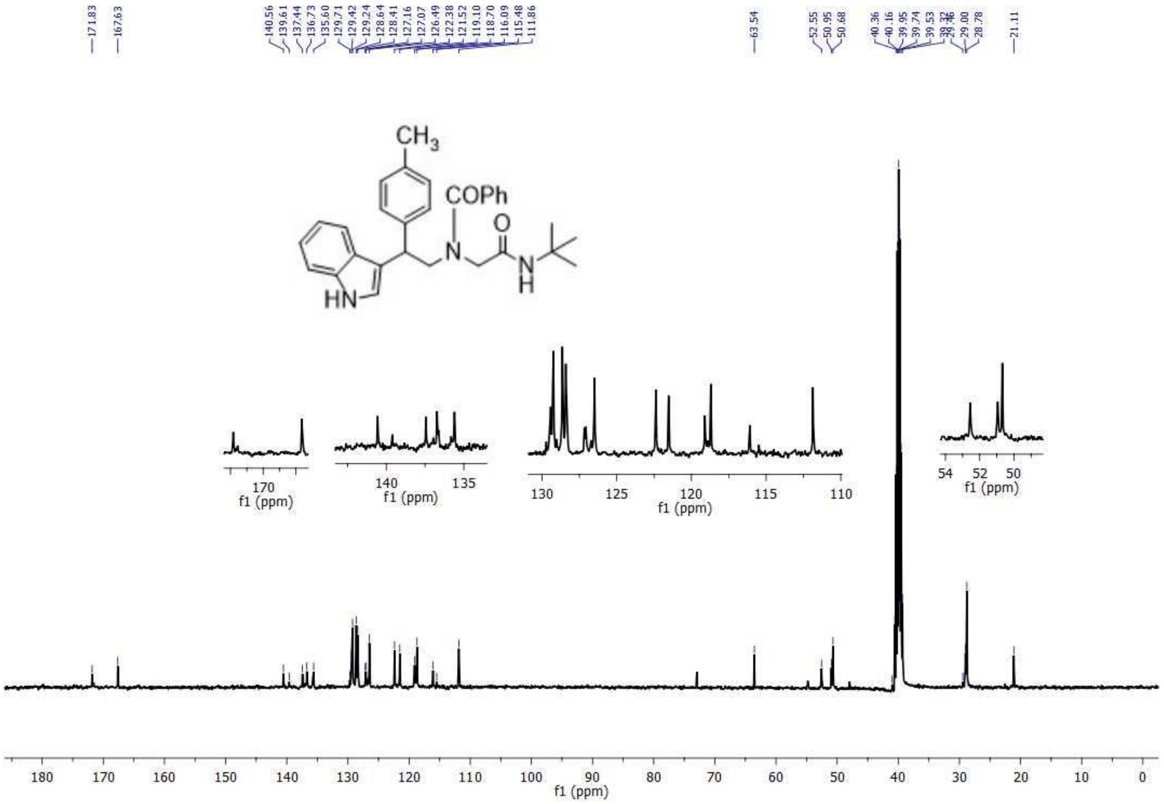

Figure S53. ^{13}C NMR of **12b** (DMSO-d_6, 400 MHz, mixture of rotamers).

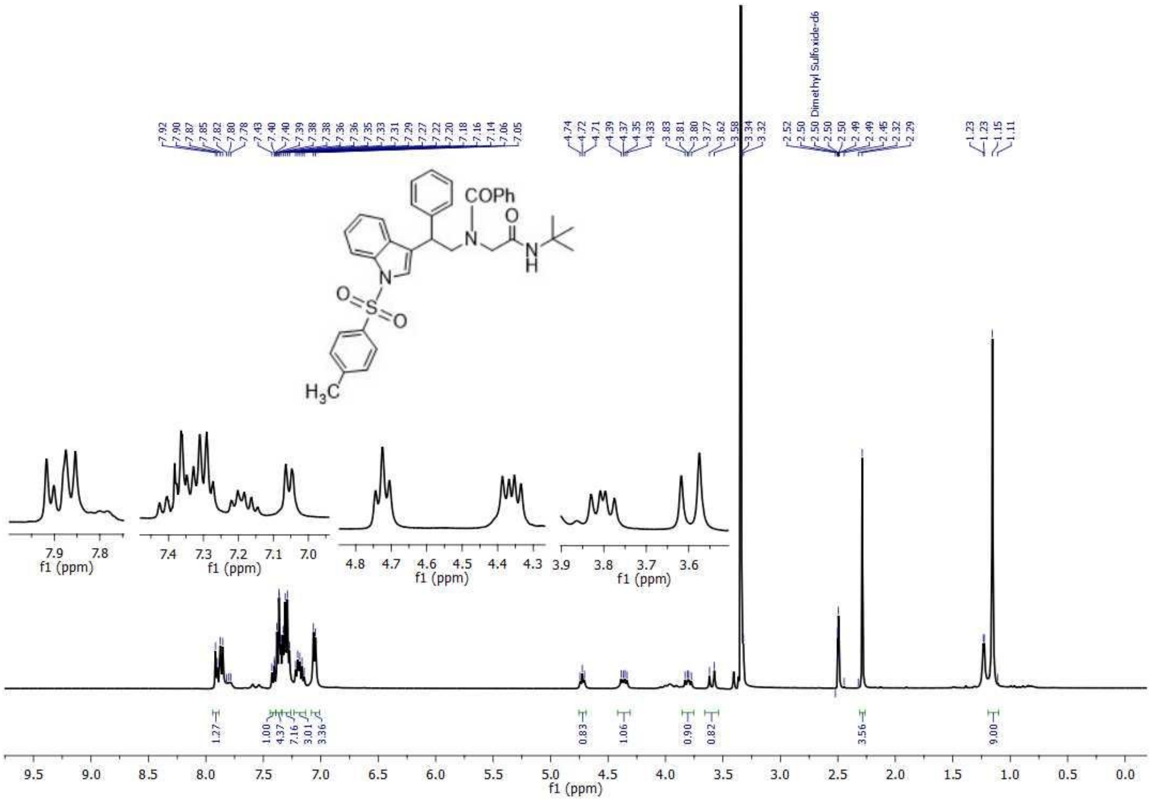

Figure S54. ^{1}H NMR **12c** (DMSO-d_6, 400 MHz, mixture of rotamers).

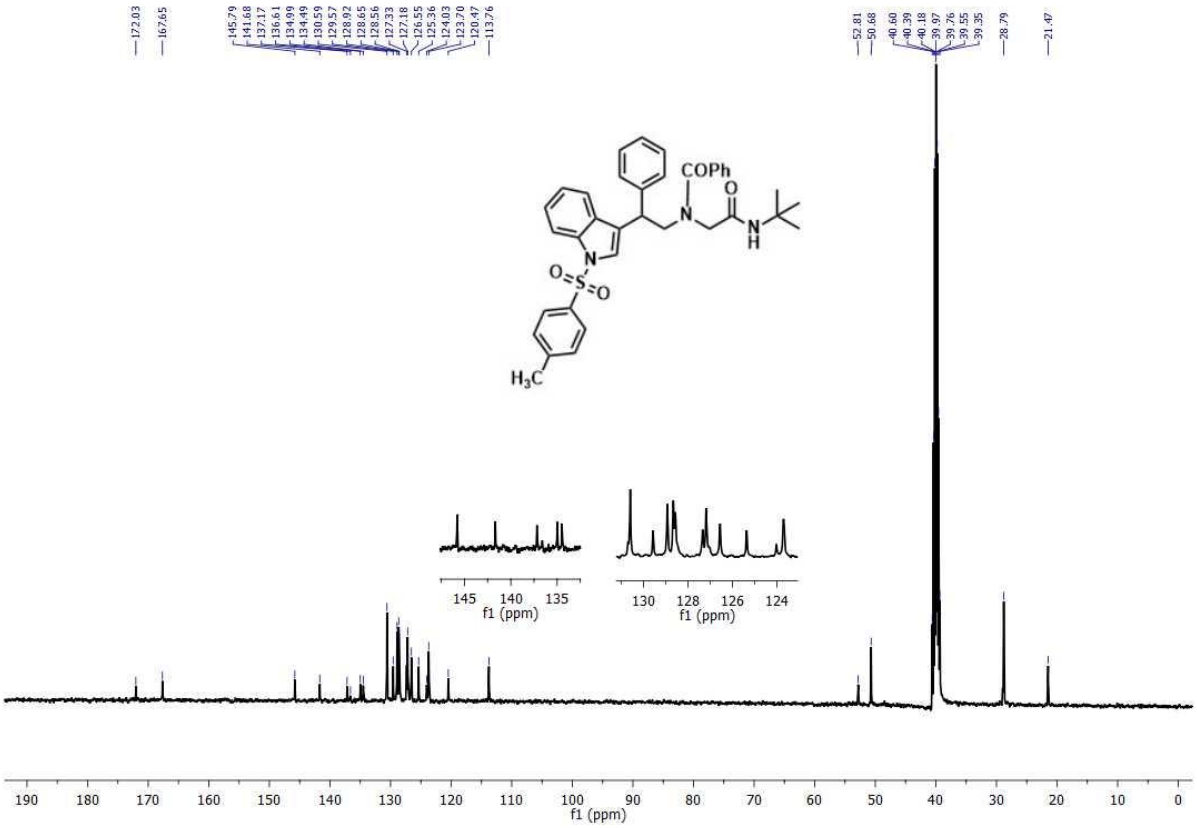

Figure S55. ^{13}C NMR of **12c** (DMSO-d_6, 400 MHz, mixture of rotamers).

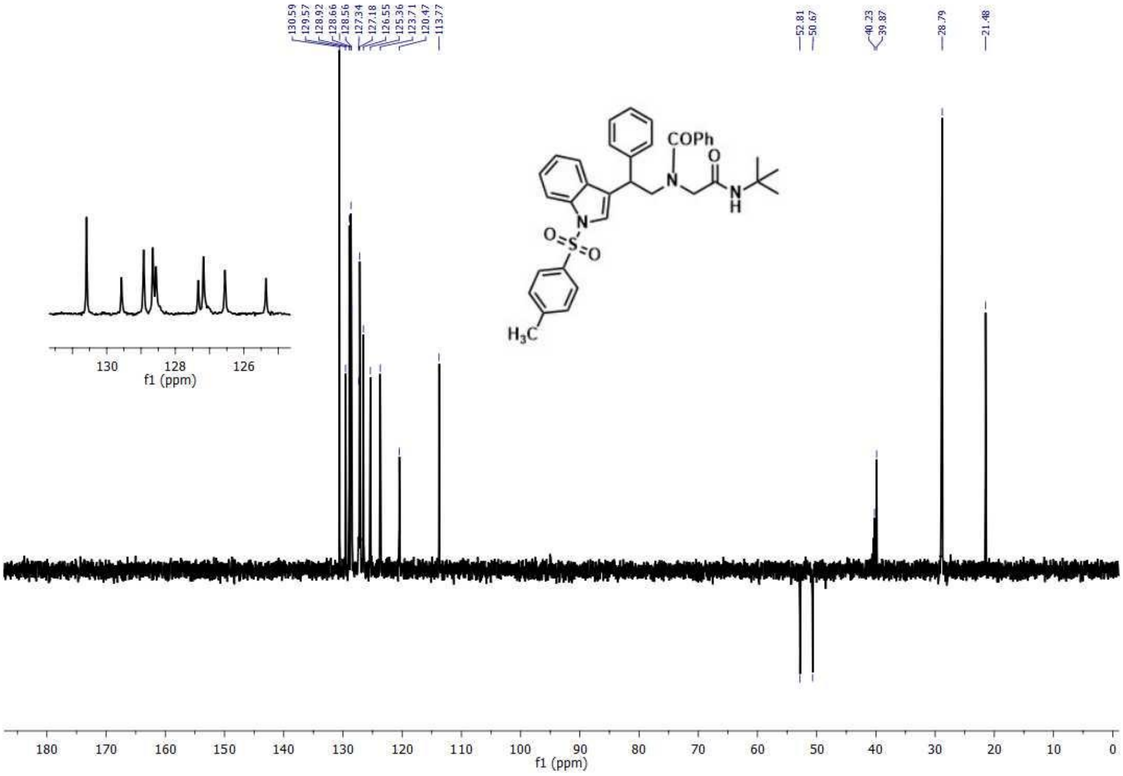

Figure S56. DEPT135 NMR of **12c** (DMSO-d_6, 400 MHz, mixture of rotamers).

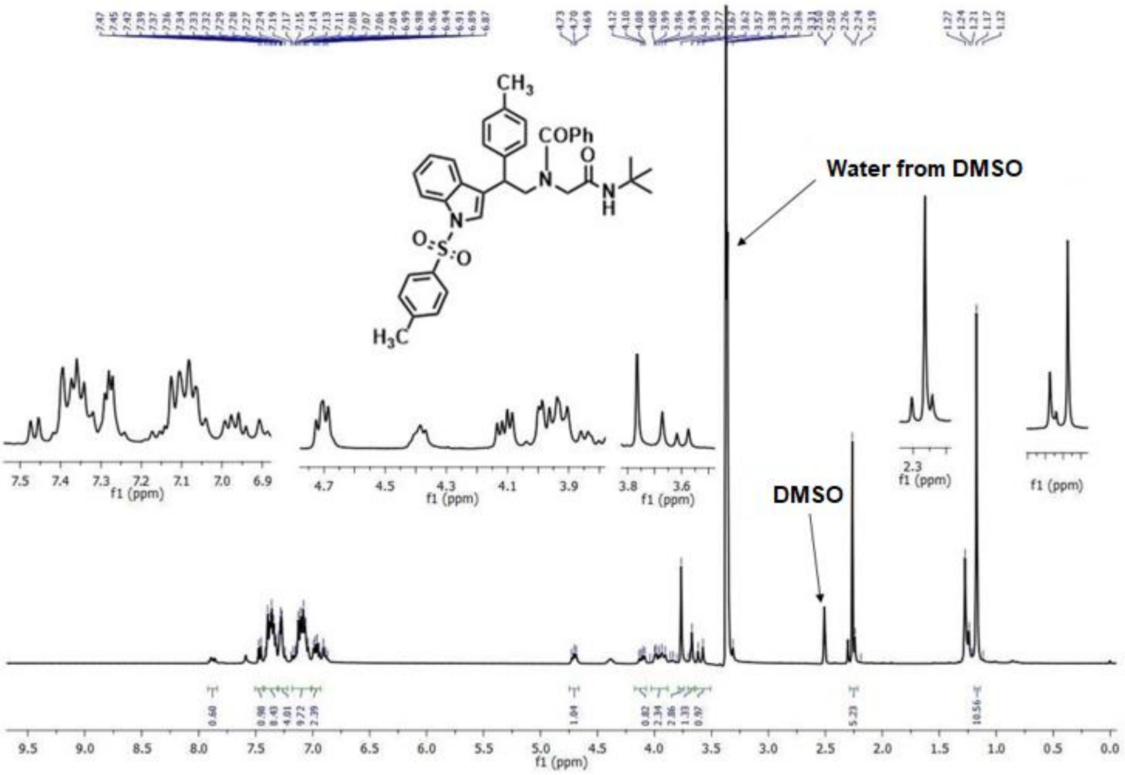

Figure S57. ^{1}H NMR of **12d** (DMSO-d_6, 400 MHz, mixture of rotamers).

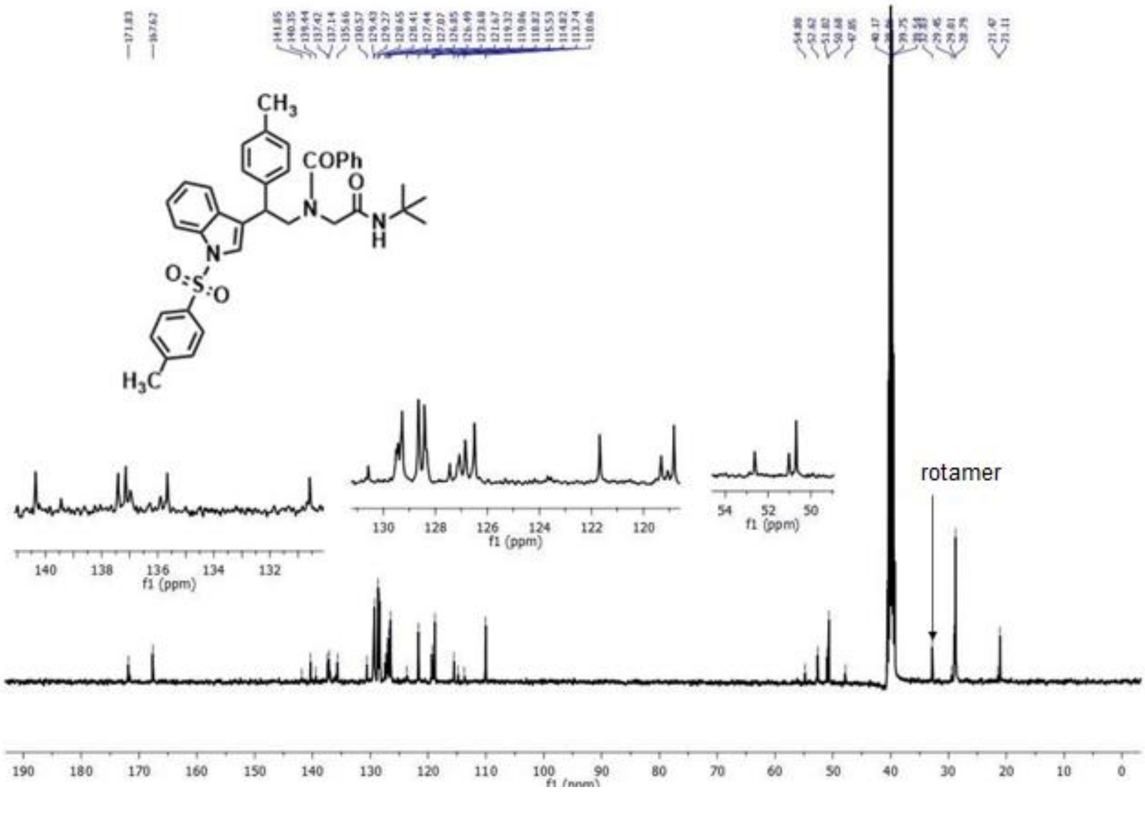

Figure S58. ^{13}C NMR of **12d** (DMSO-d_6,100 MHz, mixture of rotamers).

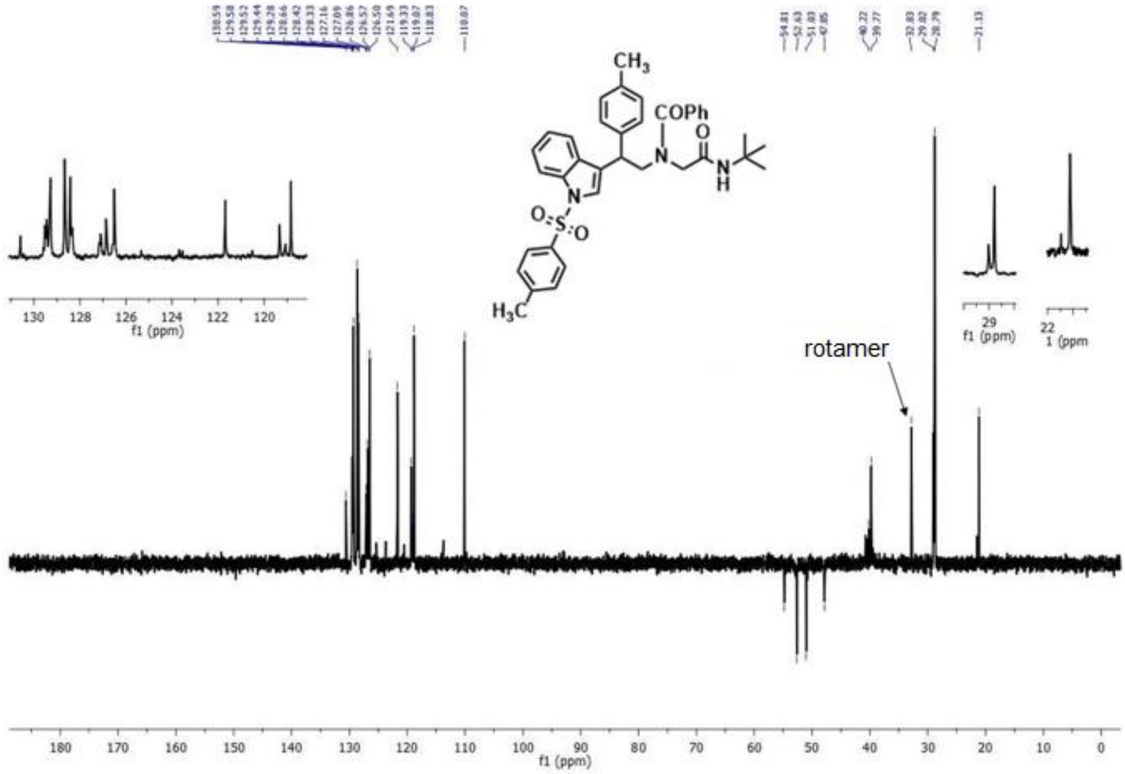

Figure S59. DEPT135 NMR of **12d** (DMSO-d_6,100 MHz, mixture of rotamers).

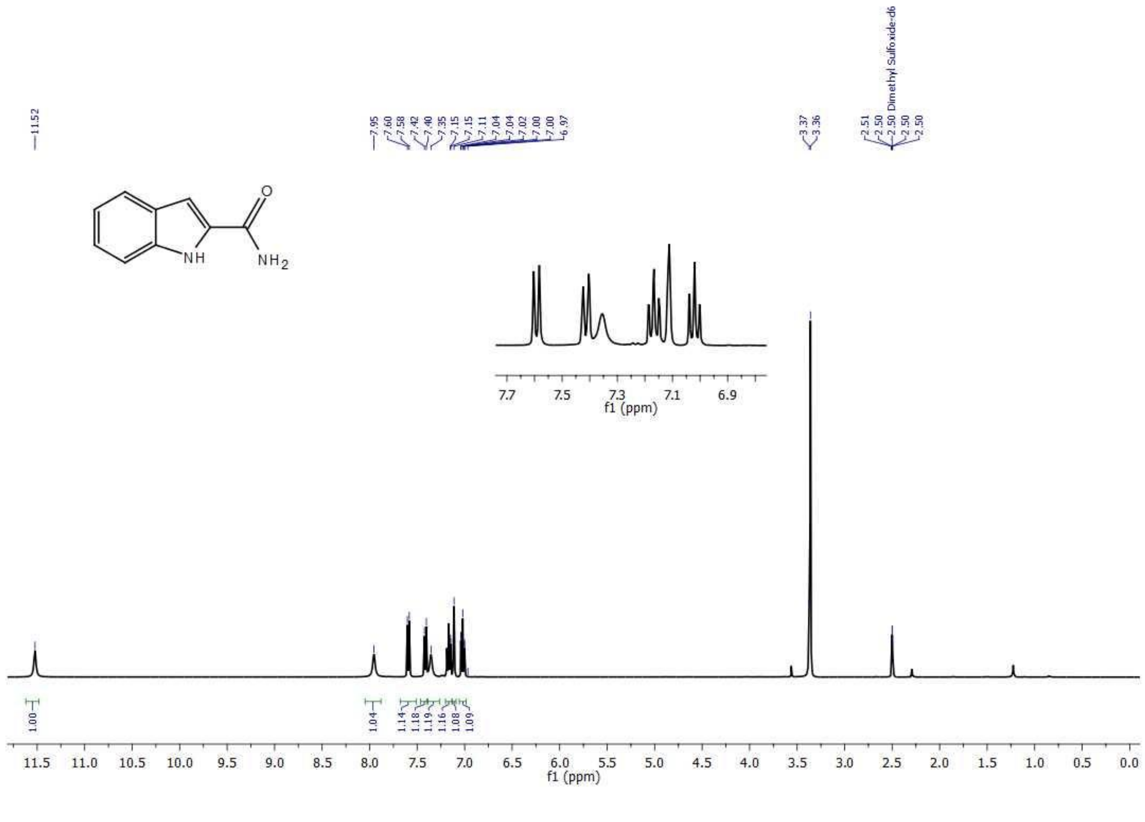

Figure S60. ^{1}H NMR **7** (DMSO-d_6, 400 MHz).

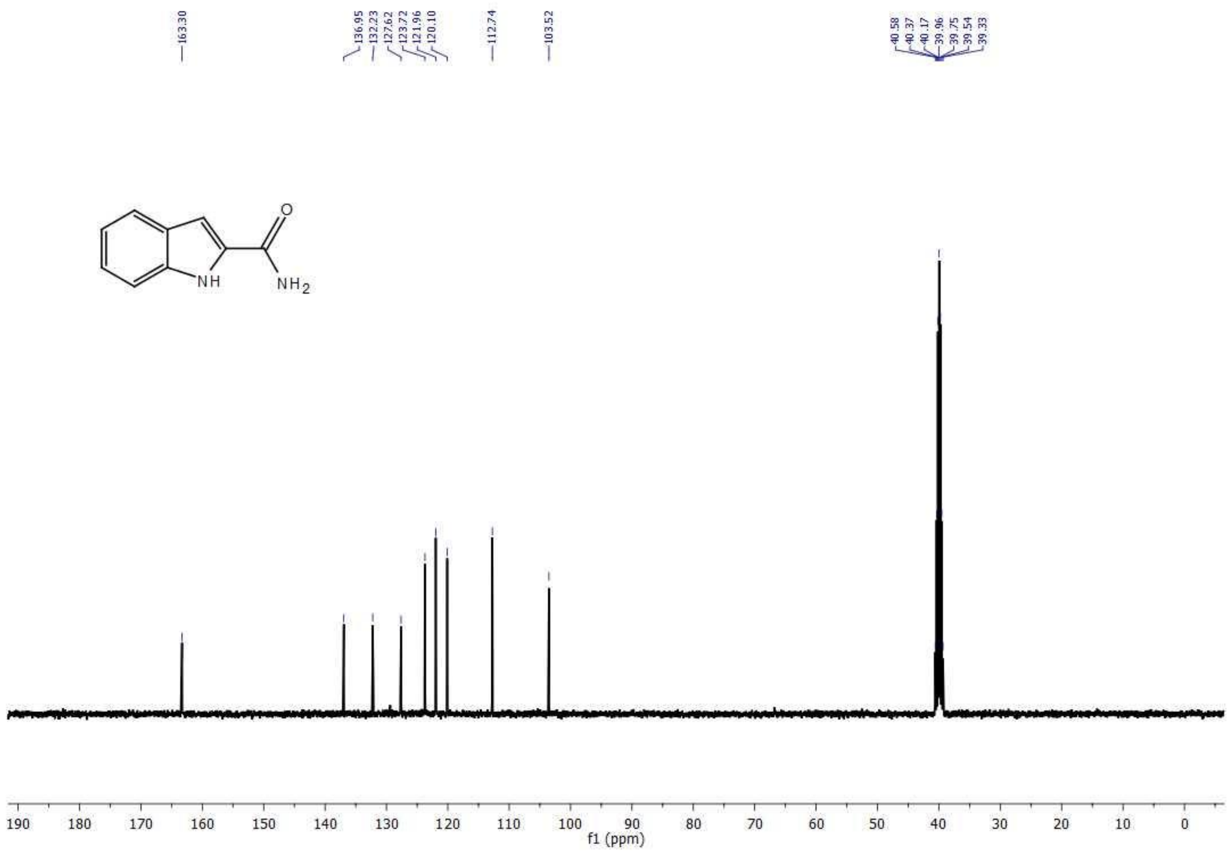

Figure S61. ^{13}C NMR of **7** (DMSO-d_6, 400 MHz).

UPCC analysis of peptidomimetics

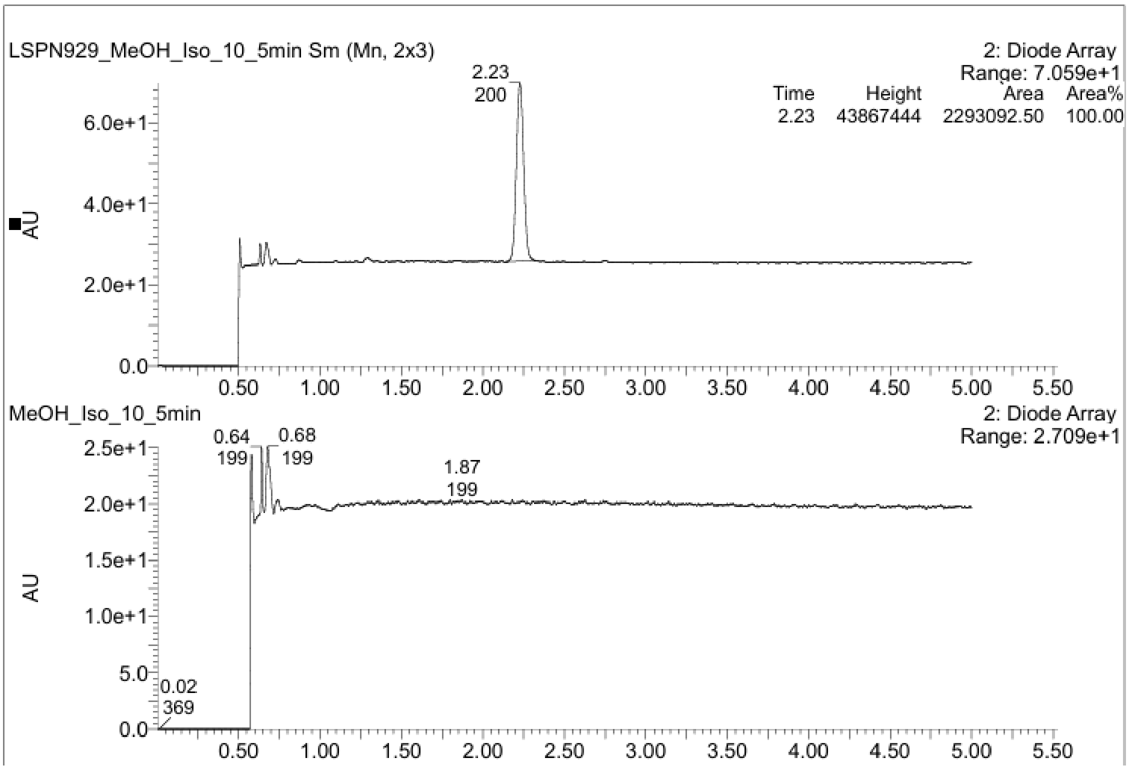

Figure S62. Chromatogram of compound **LSPN929.**

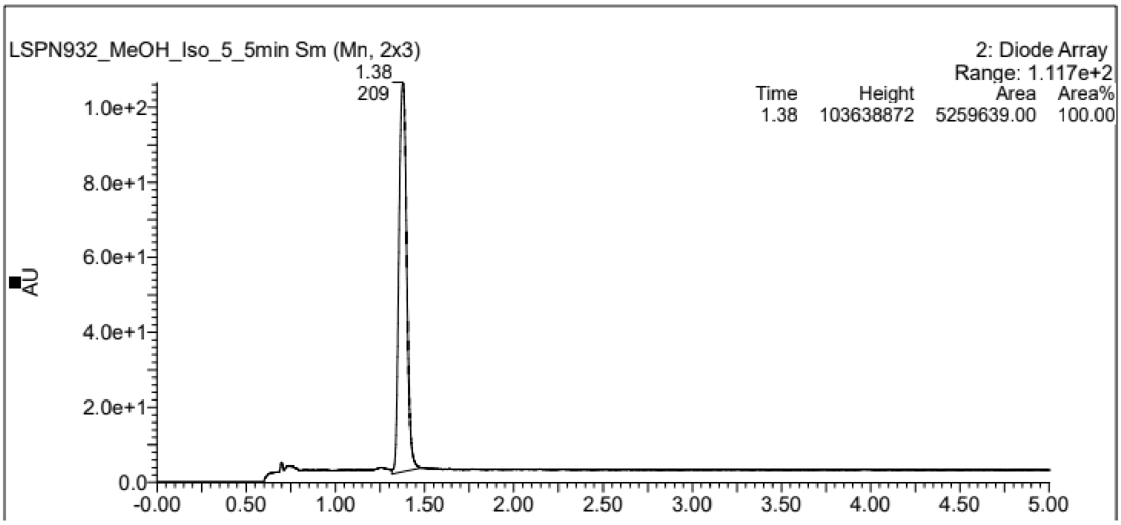

Figure S63. Chromatogram of compound **LSPN932.**

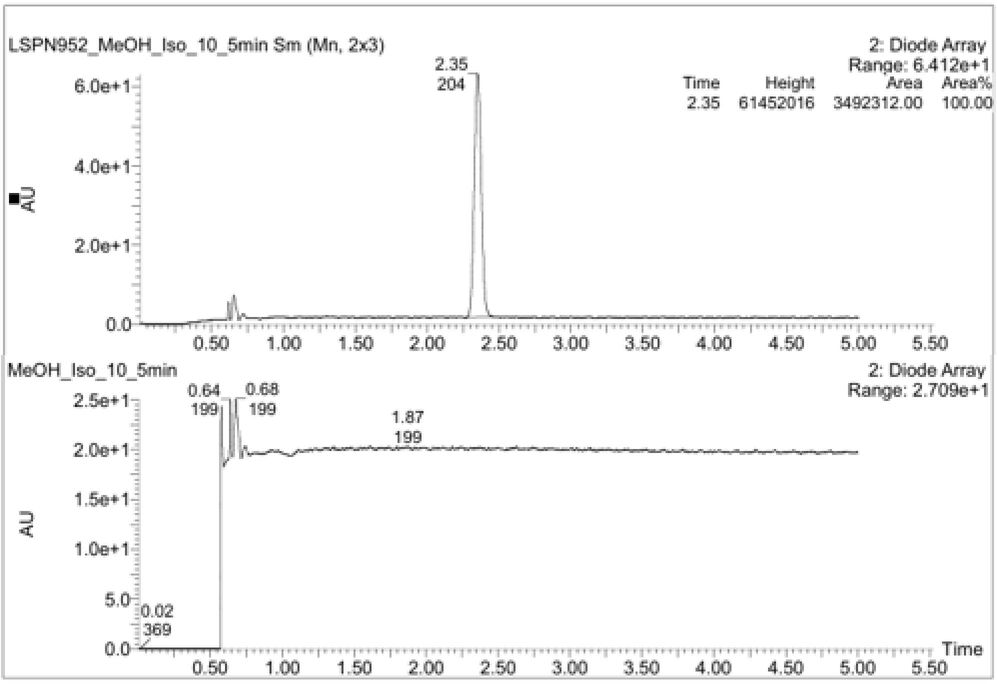

Figure S64. Chromatogram of compound **LSPN952.**

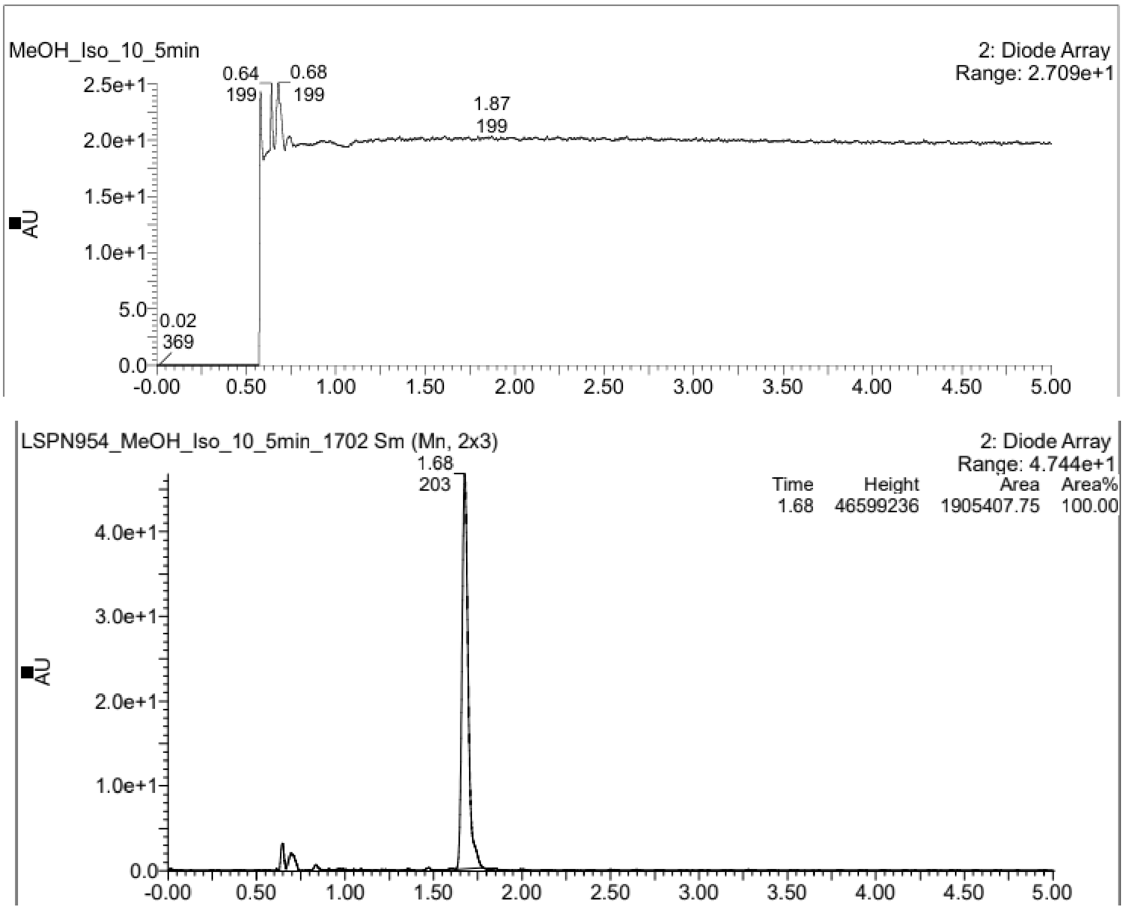

Figure S65. Chromatogram of compound **LSPN954.**

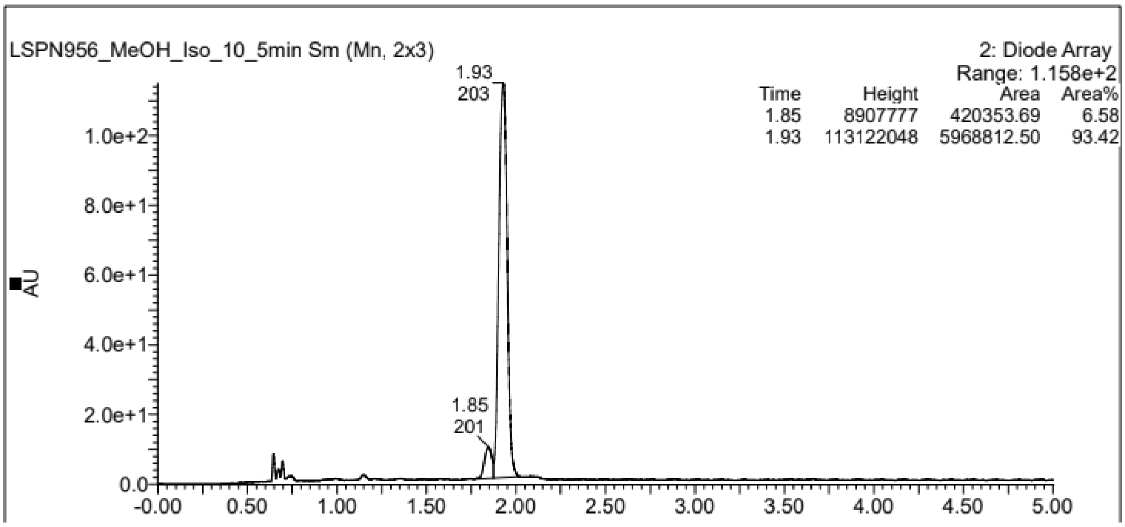

Figure S66. Chromatogram of compound **LSPN956.**

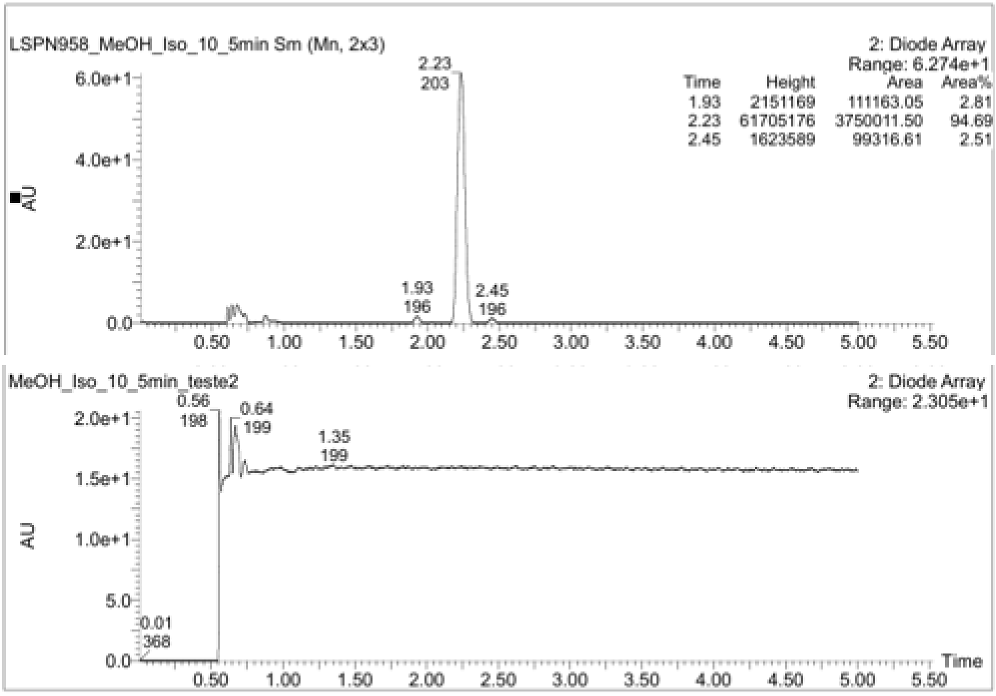

Figure S67. Chromatogram of compound **LSPN958.**

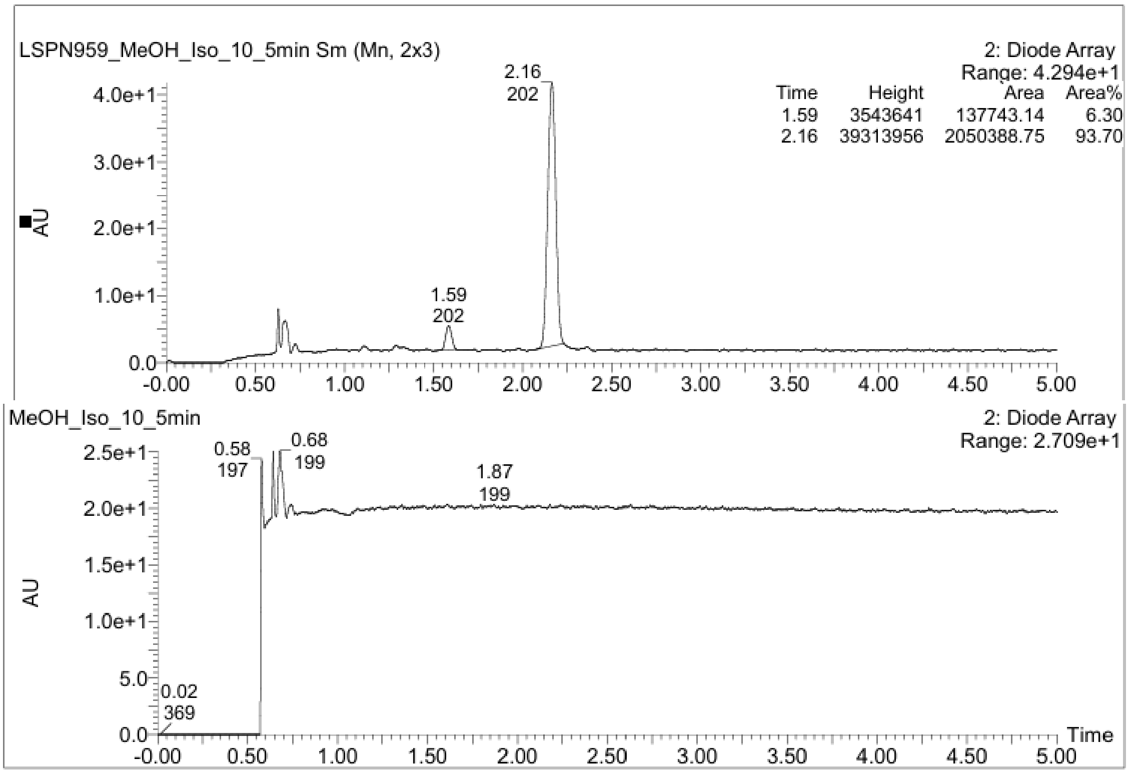

Figure S68. Chromatogram of compound **LSPN959.**

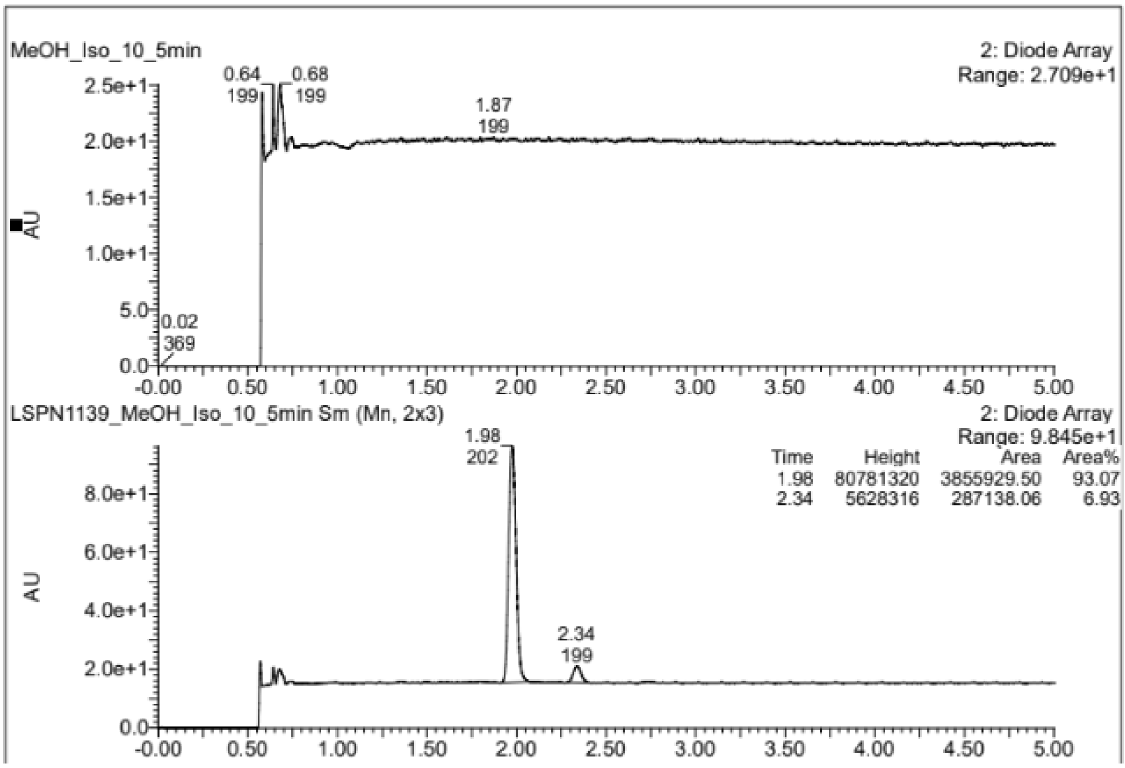

Figure S69. Chromatogram of compound **LSPN1139.**

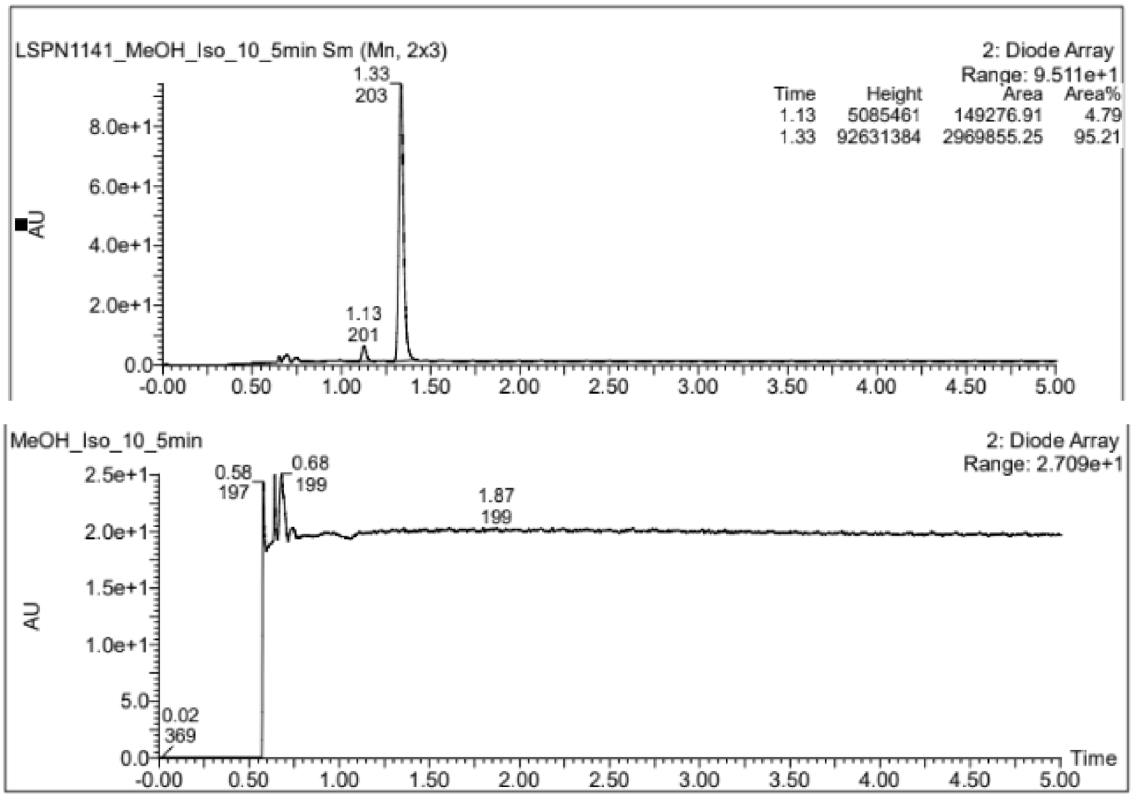

Figure S70. Chromatogram of compound **LSPN1141.**

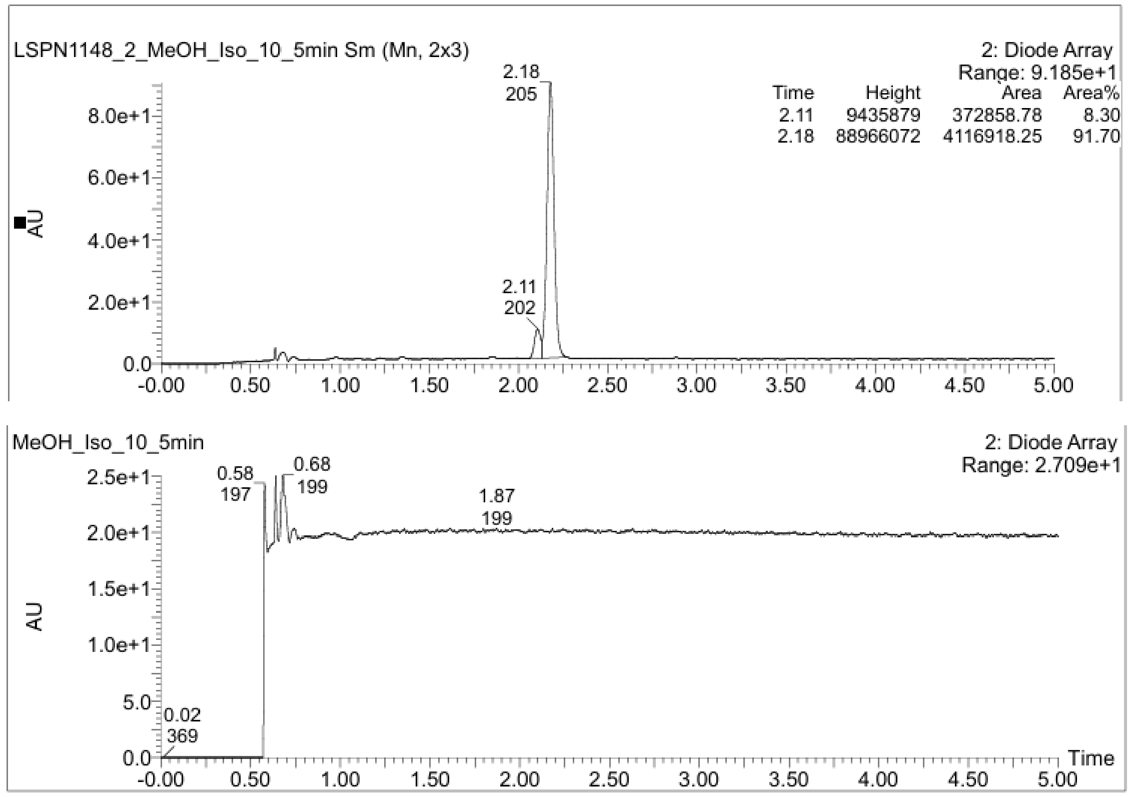

Figure S71. Chromatogram of compound **LSPN1148.**

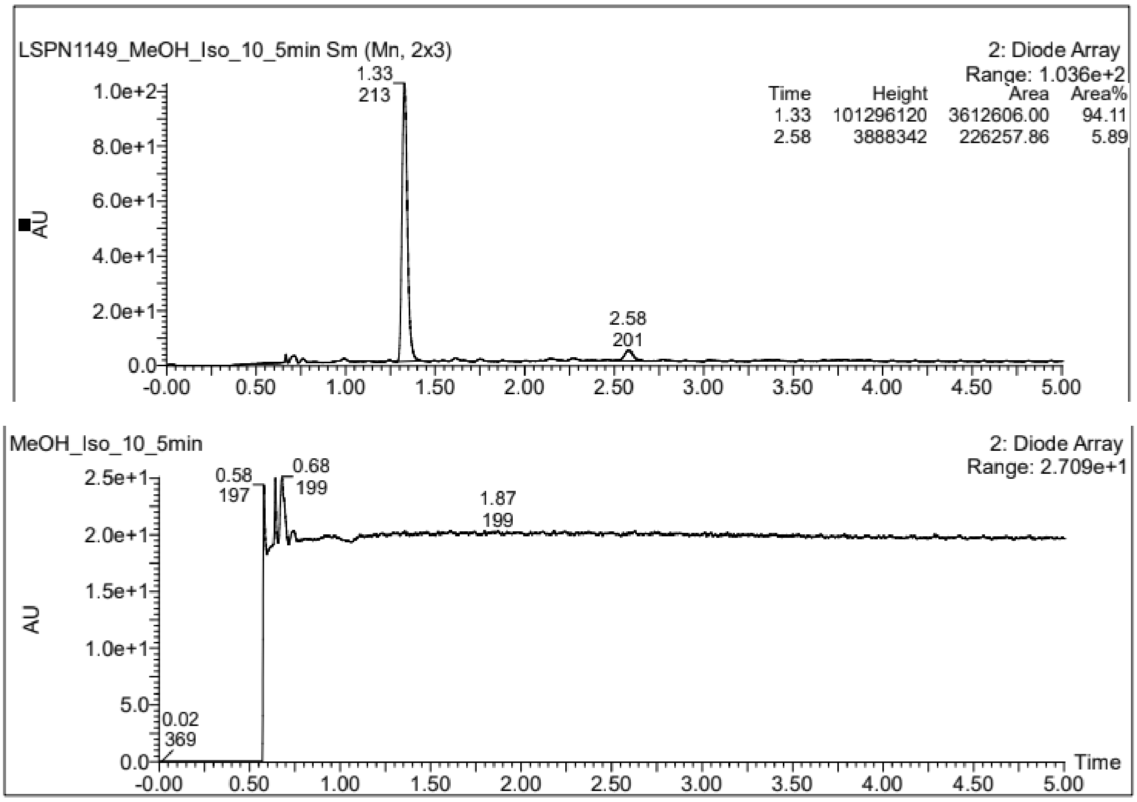

Figure S72. Chromatogram of compound **LSPN1149.**

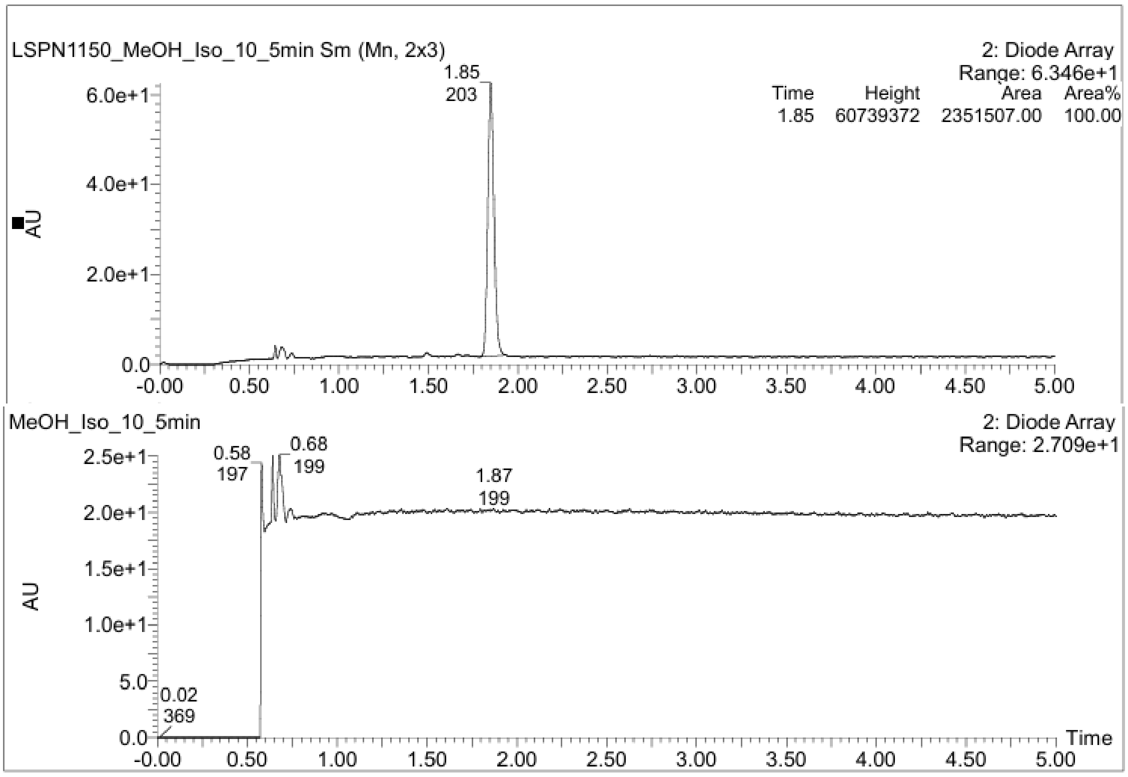

Figure S73. Chromatogram of compound **LSPN1150.**

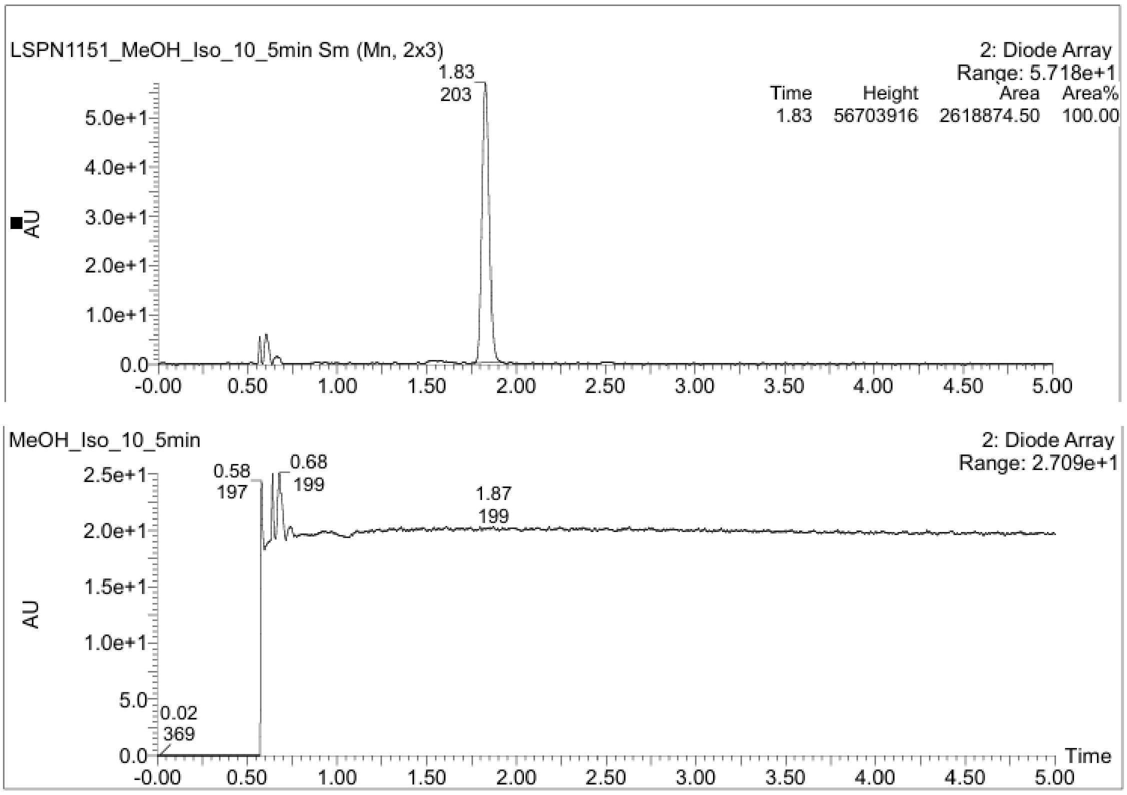

Figure S74. Chromatogram of compound **LSPN1151.**

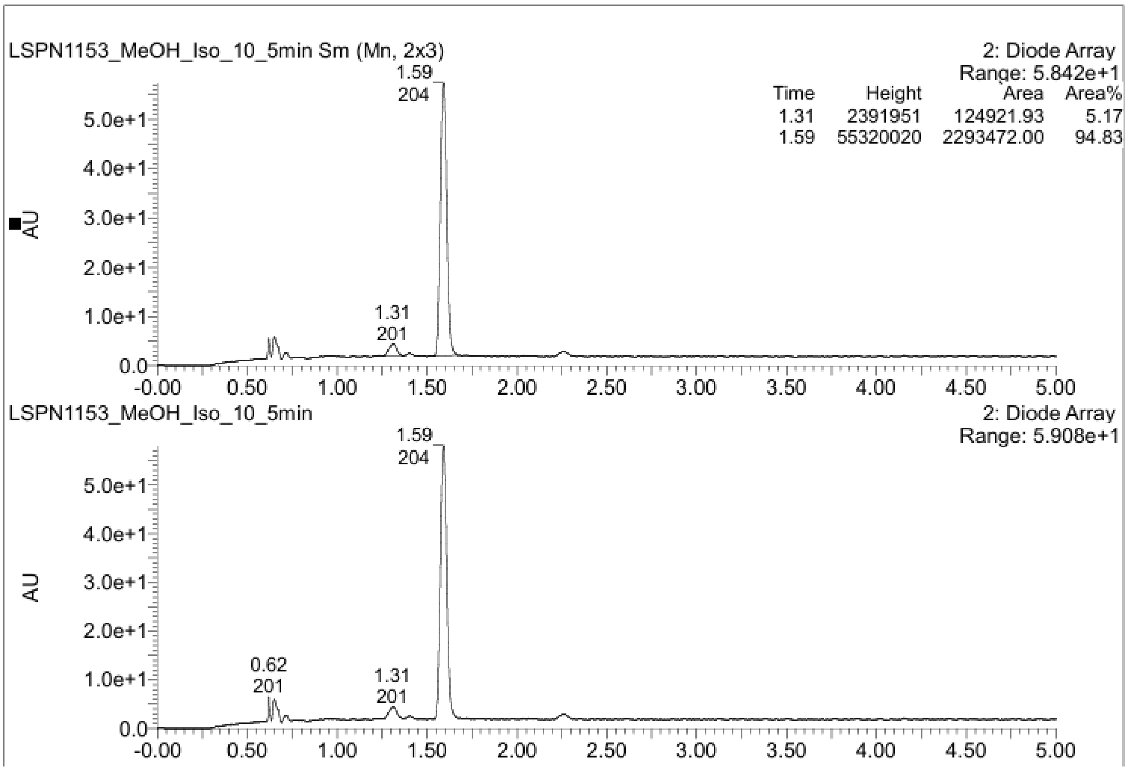

Figure S75. Chromatogram of compound **LSPN1153.**

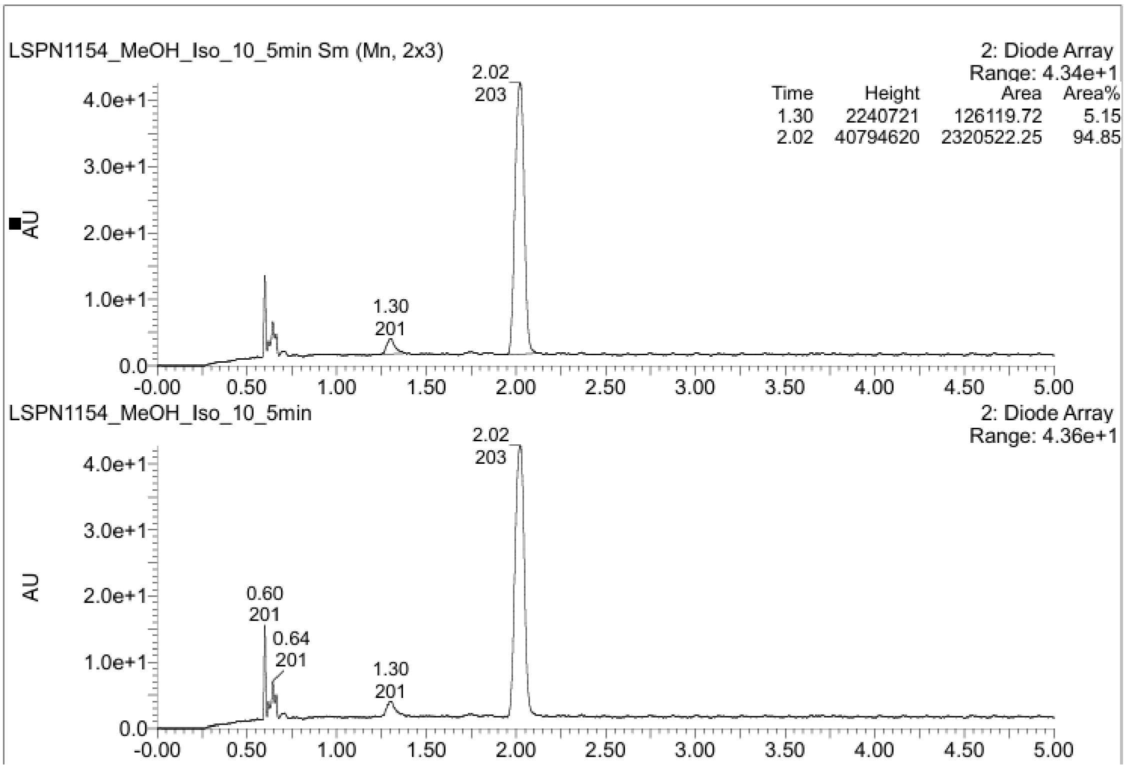

Figure S76. Chromatogram of compound **LSPN1154.**

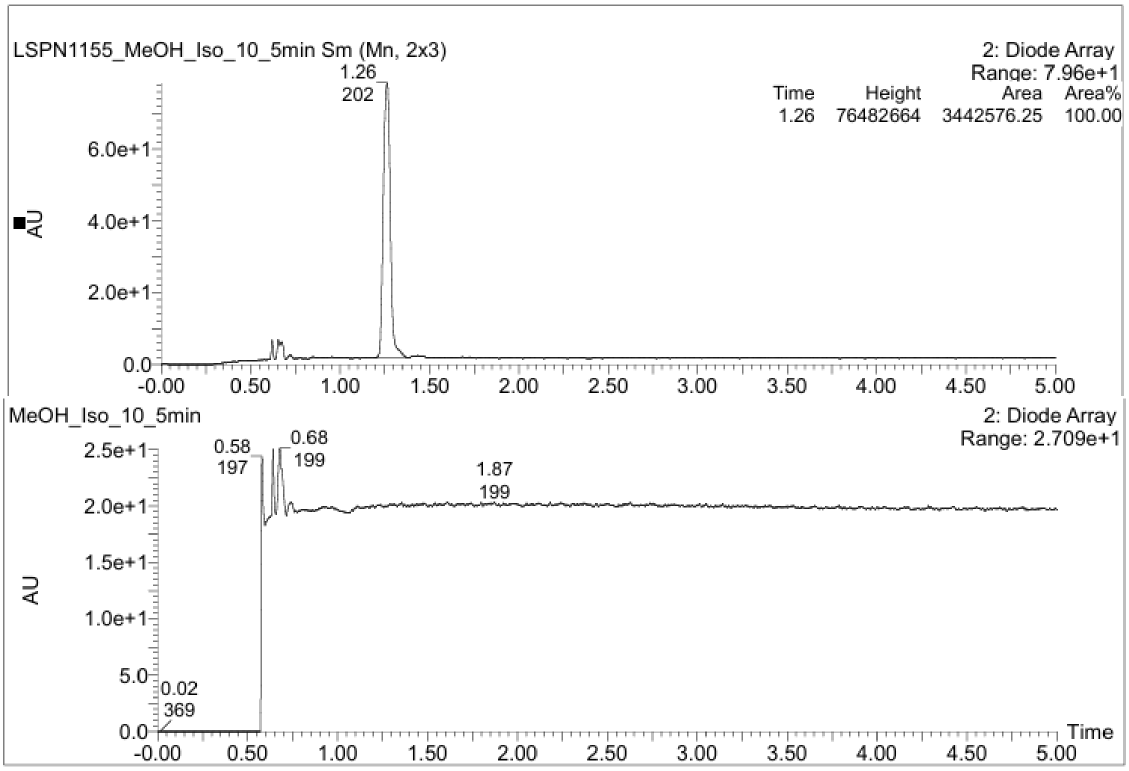

Figure S77. Chromatogram of compound **LSPN1155.**

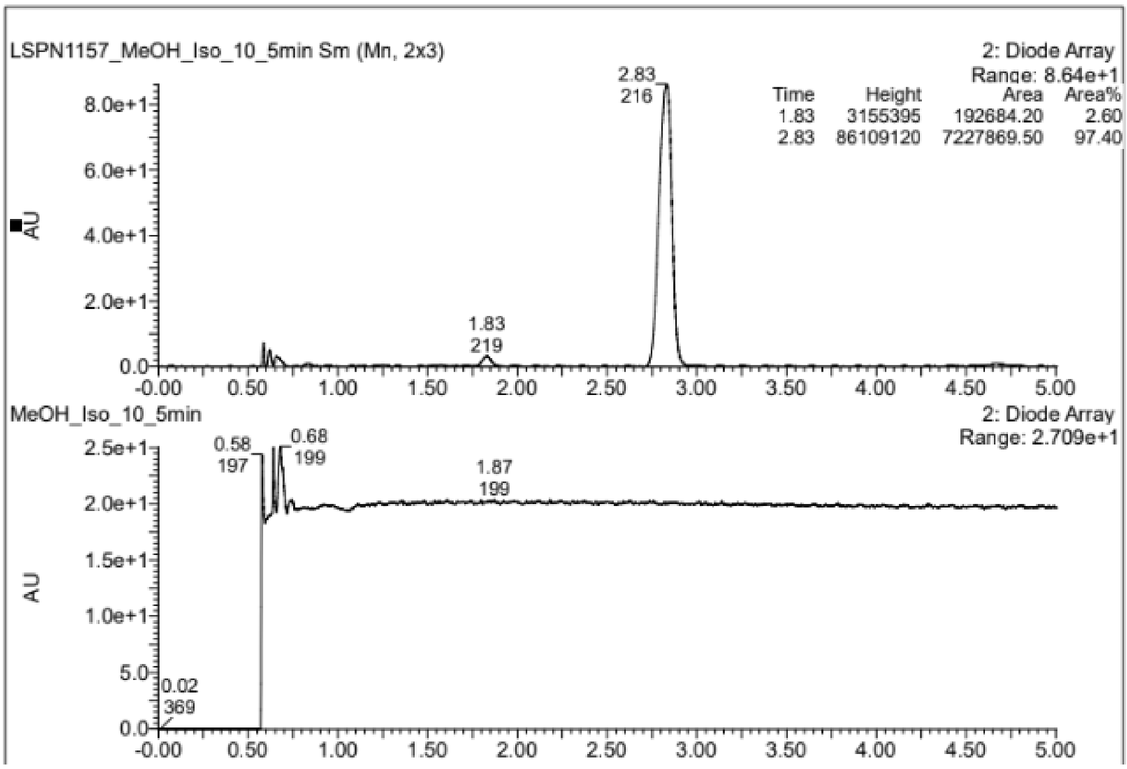

Figure S78. Chromatogram of compound **LSPN1157.**

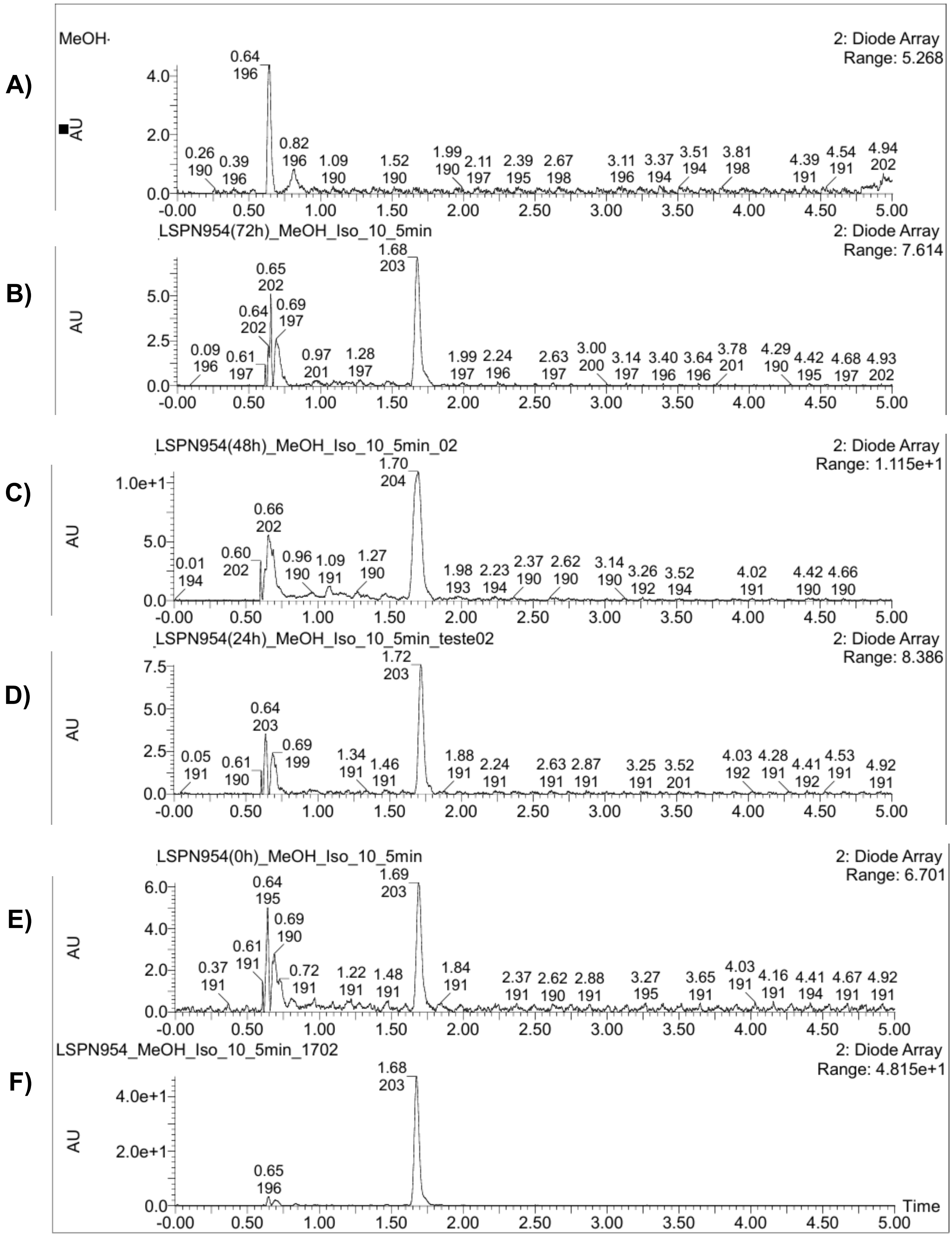

Figure S79. Stability test of compound **LSPN954**: A) MeOH, B) 72 h, C) 48 h, D) 24 h, E) 0 h, and F) compound **LSPN954** standard.

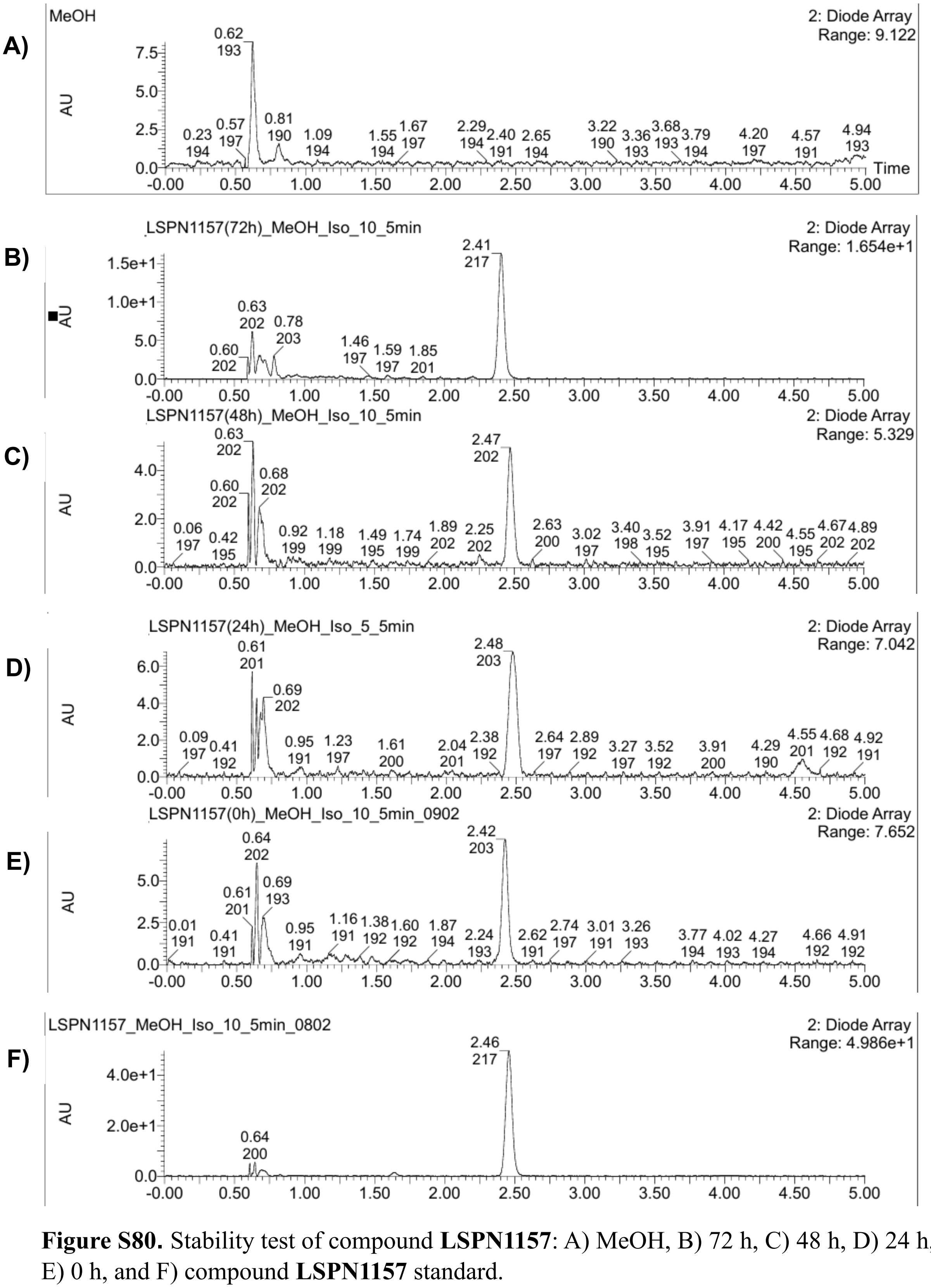

Figure S80. Stability test of compound **LSPN1157**: A) MeOH, B) 72 h, C) 48 h, D) 24 h, E) 0 h, and F) compound **LSPN1157** standard.

REFERENCES

(1) Yeh, E.; DeRisi, J. L. Chemical Rescue of Malaria Parasites Lacking an Apicoplast Defines Organelle Function in Blood-Stage *Plasmodium falciparum*. *PLoS Biol.* **2011**, *9* (8), e1001138. https://doi.org/10.1371/journal.pbio.1001138.
